# Synthesis of disparlure and monachalure enantiomers from 2,3-butanediacetals

**DOI:** 10.3762/bjoc.16.57

**Published:** 2020-04-03

**Authors:** Adam Drop, Hubert Wojtasek, Bożena Frąckowiak-Wojtasek

**Affiliations:** 1 Institute of Chemistry, Opole University, ul. Oleska 48, 45-052 Opole, Poland; 2 ZWP EMITOR S.C., ul. Olimpijska 6, 45-681 Opole, Poland

**Keywords:** 2,3-butanediacetal, *cis*-epoxide, (−)-disparlure, (+)-disparlure, (−)-monachalure, (+)-monachalure

## Abstract

2,3-Butanediacetal derivatives were used for the stereoselective synthesis of unsymmetrically substituted *cis*-epoxides. The procedure was applied for the preparation of both enantiomers of disparlure and monachalure, the components of the sex pheromones of the gypsy moth (*Lymantria dispar*) and the nun moth (*Lymantria monacha*) using methyl (2*S*,3*R*,5*R*,6*R*)-3-ethylsulfanylcarbonyl-5,6-dimethoxy-5,6-dimethyl-1,4-dioxane-2-carboxylate as the starting material.

## Introduction

Compounds containing chiral epoxides display a wide range of biological activities and a number of them are semiochemicals. They have been frequently found as pheromones in lepidopteran species of the families Noctuidae, Arctiidae, Lymantridae, Geometridae and Erebidae [1]. (3*Z*,9*Z*)-6*S*,7*R*-Epoxyheneicosa-3,9-diene, a constituent of the female sex pheromone of the moth *Tetanolita mynesalis* (Erebidae) [2] is a particularly interesting example, because it is also a component of the luring mixture used by the bolas spider *Mastophora hutchinsoni* [3–4]. (6*R*,7*S,2E*)-6,7-Epoxy-2-nonenal has been identified as a sex-aggregation pheromone of the red-necked longhorn beetle (*Aromia bungii*) [5] and its synthesis has been recently developed by Mori and co-workers [6–7]. (2*E*)-*trans*-4,5-Epoxy-2-decenal is present in mammalian blood and may be used by predators to track their prey [8–9]. A classical example of *cis*-epoxides with semiochemical activity is the chemical communication system of the gypsy moth (*Lymantria dispar*) and the nun moth (*Lymantria monacha*). The sex pheromone of the gypsy moth contains (+)-disparlure (**1**, (7*R*,8*S*)-7,8-epoxy-2-methyloctadecane) [10]. The pheromone blend of the nun moth contains (−)-disparlure (**3**, (7*S*,8*R*)-7,8-epoxy-2-methyloctadecane), (+)-monachalure (**2**, (7*R*,8*S*)-*cis*-7,8-epoxyoctadecane), (−)-monachalure (**4**, (7*S*,8*R*)-*cis*-7,8-epoxyoctadecane) and their olefinic precursors in addition to (+)-disparlure (**1**) [11–12]. For the gypsy moth a high enantiomeric purity of (+)-disparlure (>98% ee) is required to evoke the male’s sexual response and even a small percentage of (−)-disparlure abolishes the biological activity, thus preventing cross-species attraction [11].

**Scheme 1:**
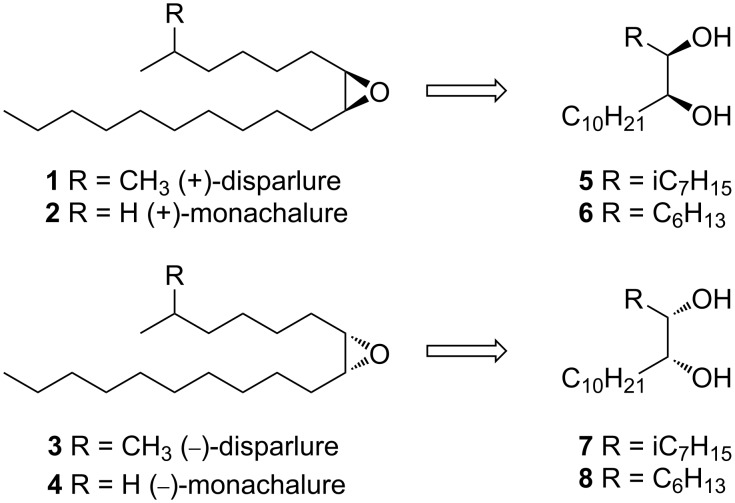
Retrosynthesis of (+)-disparlure (**1**), (−)-disparlure (**3**), (+)-monachalure (**2**), and (−)-monachalure (**4**) from diols **5**–**8**.

Due to the great economic damage caused by the gypsy moth and the nun moth, many synthetic routes for the preparation of (+)-disparlure (**1**) have been developed to monitor and control their populations. These methods use various approaches to ensure the required configuration of the asymmetric centers determining the biological activity of this compound. They include the use of naturally occurring chiral substrates, such as ʟ-glutamic acid [13], ʟ-tartaric acid [14–16], ᴅ-glucose [17], and sorbitol [18] as starting materials, as well as enantioselective reactions, such as the Sharpless epoxidation [19–24], asymmetric dihydroxylation [25–26], chloroallyloboronation [27], or iodolactonization [28]. Most recently a method using the asymmetric chlorination of dodecanal by LiCl in the presence of a chiral imidazolidinone catalyst has also been described [29]. However, many of these methods have some drawbacks – the most important one being the insufficient enantiomeric purity for biological and commercial applications [29]. (+)-Disparlure used in most commercial lures is prepared by the Sharpless epoxidation reaction, which gives the final product with ees not exceeding 95% [29], which is insufficient to be used as attractant for males of the Gypsy moth. In one of these methods the diols **5** and **7** were used as precursors in the synthesis of the *cis*-epoxides **1** and **3** (Scheme 1). A few methods for the synthesis of diols **5** and **7** have been described [26,30–31] and we decided to prepare them starting from 2,3-butanediacetal derivatives of ʟ-tartaric acid. This method uses cheap and easily available starting materials, does not require expensive chiral catalysts and offers the possibility of obtaining both enantiomers of disparlure and monachalure with high enantiomeric purity. It can also be adapted for preparation of other chiral *cis*-epoxides.

**Scheme 2:**
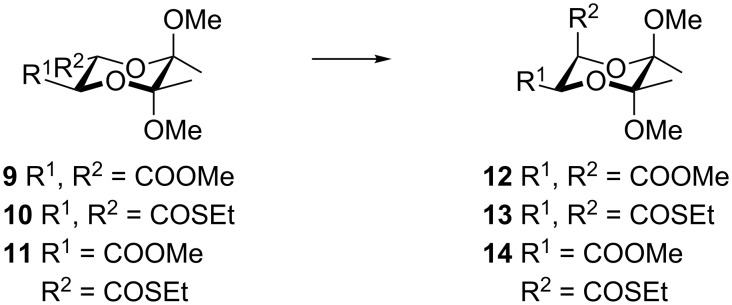
Isomerization of *trans*-2,3-butanediacetals **9**–**11** to *cis*-2,3-butanediacetals **12**–**14**.

2,3-Butanediacetals obtained from tartaric acid dimethyl diester contain protected hydroxy groups and two functional groups suitable for the attachment of various substituents. *trans*-2,3-Disubstituted butanediacetal derivatives of dimethyl tartrates **9**–**11** can be converted to *cis*-isomers **12**–**14** (Scheme 2) [32].

Compound **12** can be obtained from *trans*-dimethyl ester **9** in a two-step procedure either following a traditional way or using continuous flow chemistry [32–34]. The isomerization of *trans*-2,3-butanediacetals to the *cis-*isomers has also been performed via dithiolate derivative **10** to obtain compound **13** (Scheme 2) [35]. However, attempts to isomerize dimethyl ester **9** in the same way gave a cyclic compound as a major product [36]. We have recently developed a simple and efficient method for the isomerization of *trans*-disubstituted butanediacetal derivative **11** with two different substituents (one ester group and one thioester group) to its *cis* derivative **14** which proceeded with 94% yield [37]. The *cis*-2,3-butanediacetals **12**–**14** have the appropriate configuration of the chiral centers for the synthesis of both enantiomers of disparlure **1** and **3** and monachalure **2** and **4**. Compound **14** also offers the possibility of selective modification of one of the substituents as well as sequential introduction of aliphatic chains. After deprotection of the butanediacetal group, diols **5**–**8** are obtained. We therefore decided to apply this compound for the synthesis of both enantiomers of disparlure and both enantiomers of monachalure.

## Results and Discussion

**Scheme 3:**
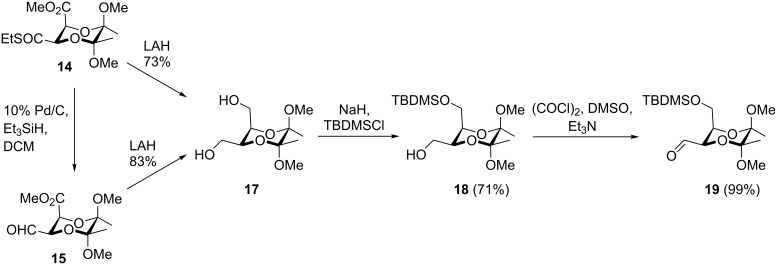
Synthesis of diol **17** and its subsequent modifications.

Compound **14** was prepared and converted to **15** by Fukuyama reduction as described previously [37]. The overall yield for the synthesis of compound **15** from ʟ-dimethyl tartrate and butanone was 32%. We initially attempted to carry out the Wittig reaction of compound **15** with *n*-pentyltriphenylphosphonium bromide and the conditions of the individual reactions are collected in Table 1. Product **16** was obtained with various yields ranging from 0 to 64% and the results were not reproducible. We could not identify the reason of these problems and could not overcome them. So, although compound **15** seemed an attractive starting material for introducing alkyl chains of pheromones **1**–**4**, we decided to abandon this substrate and use aldehyde **19** instead. This compound was obtained from both aldehyde **15** and its precursor ethyl thioester methyl ester **14**, respectively (Scheme 3). Both substrates **14** and **15** were reduced to the corresponding diol **17** with lithium aluminum hydride with 73% or 83% yield, respectively. Next, the selective protection of the axial hydroxymethyl group [32,38–39] was performed affording the product with a diastereomeric ratio of 6:1. Isomer **18** was isolated by column chromatography and subjected to a Swern oxidation to give the desired aldehyde **19** in nearly quantitative yield.

**Table 1:** Conditions of the Wittig reaction.

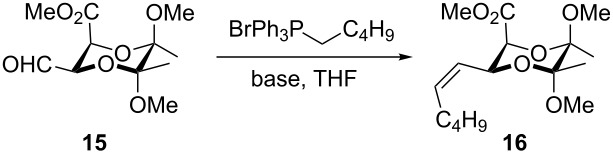			

entry	base (equiv)	ylide (equiv)	yield (%)

1	*n*-BuLi 1.2–2.0	1.2–2.0	10–53^a^
2	LiHMDS 1.1–2.1	1.1–2.2	17–64^a^
3	KHMDS 1.2–1.5	1.2-1.5	0-46^a^

^a^The yields were in a wide range and were not reproducible.

**Scheme 4:**
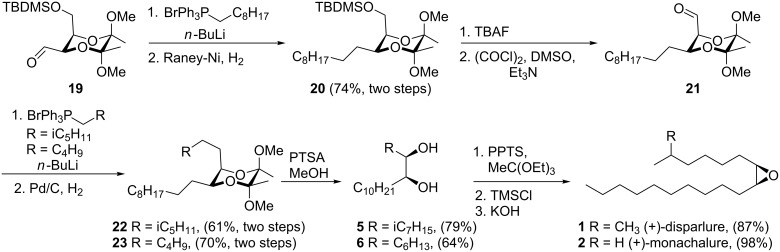
Synthesis of (+)-disparlure (**1**) and (+)-monachalure (**2**).

This aldehyde derivative **19** was then used in the synthesis of (+)-disparlure (**1**), (−)-disparlure (**3**), (+)-monachalure (**2**), and (−)-monachalure (**4**). First, the (+)-enantiomers **1** and **2** were synthesized (Scheme 4).

In a Wittig reaction with *n*-nonyltriphenylphosphonium bromide followed by reduction of the double bond, the longer chain of the pheromone structures was attached giving compound **20** in 74% yield. Next, removal of the protecting group from the secondary alcohol followed by a Swern oxidation gave aldehyde **21** which was subjected to Wittig reactions with either of the two different alkyl derivatives and catalytic hydrogenation, giving compounds **22** and **23**. The butanediacetal groups were then removed with *p*-toluenesulfonic acid and diols **5** and **6** were obtained with 79% and 64% yield, respectively. They were then used in a well-established three-step, one-pot procedure for epoxide ring closure [26,30,40–41]. Pure (+)-disparlure (**1**) and (+)-monachalure (**2**) were obtained after column chromatography with 87% and 98% yield, respectively.

**Scheme 5:**
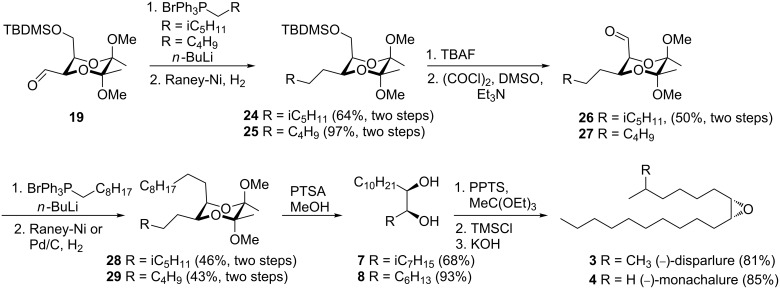
Synthesis of (−)-disparlure (**3**) and (−)-monachalure (**4**).

For the synthesis of the (−)-enantiomers **3** and **4** the sequence of attachment of alkyl chains to aldehyde **19** was reversed (Scheme 5). The Wittig reactions with 4-methylpentyltriphenylphosphonium bromide or *n*-pentyltriphenylphosphonium bromide followed by reduction of the double bonds were performed first giving derivatives **24** and **25** containing the shorter carbon chains of the pheromones. Aldehydes **26** and **27** were obtained after removal of the protecting groups from the alcohols in compounds **24** and **25**, followed by Swern oxidation. The second Wittig reactions with the longer alkyl derivative and hydrogenation gave compounds **28** and **29**. The diols **7** and **8** were then obtained by cleavage of the butanediacetals and were used for the synthesis of pheromones **3** and **4** using the procedure described previously for the synthesis of the (+)-enantiomers **1** and **2** with 81% and 85% yield, respectively.

## Conclusion

The synthesis of pheromones **1**–**4** was achieved using stable butanediacetal derivatives as the starting materials. All intermediates are stable compounds and could be easily purified. The procedure may also be applied for the preparation of other unsymmetrically substituted *cis*-epoxides. The overall yields, starting from aldehyde **19**, were 21% for (+)-disparlure, 6% for (−)-disparlure, 22% for (+)-monachalure, and 23% for (−)monachalure. The optical rotation of the obtained (+)-disparlure (**1**) and (−)-disparlure (**3**) are in agreement with the literature data. For the isomers of monachalure **2** and **4** the optical rotations have been measured for the first time and were low, similar to the values of the disparlure enantiomers **1** and **3**.

## Supporting Information

File 1 Experimental procedures of the synthesized compounds.

File 2 Copies of ^1^H NMR and ^13^C NMR spectra.
