# Supplementary material for: Synthesis of disparlure and monachalure enantiomers from 2,3-butanediacetals
Source: Beilstein J Org Chem. 2020 Apr 3;16:616–20. doi: 10.3762/bjoc.16.57 (PMC7136567; doi:10.3762/bjoc.16.57)
Supplement: File 1 — Experimental procedures of the synthesized compounds. [file Beilstein_J_Org_Chem-16-616-s001.pdf]

BEILSTEIN JOURNAL OF ORGANIC CHEMISTRY

Supporting Information

for

Synthesis of disparlure and monachalure enantiomers from 2,3-butanediacetals

Adam Drop, Hubert Wojtasek and Bożena Frąckowiak-Wojtasek

Beilstein J. Org. Chem. **2020,** *16,* 616–620. doi:10.3762/bjoc.16.57

Experimental procedures of the synthesized compounds

License and Terms: This is a supporting information file under the terms of the Creative Commons Attribution License (http://creativecommons.org/licenses/by/4.0). Please note that the reuse, redistribution and reproduction in particular requires that the authors and source are credited.
The license is subject to the *Beilstein Journal of Organic Chemistry* terms and conditions: (https://www.beilstein-journals.org/bjoc)

Table of contents

		General information	S3
1.		Physical constants and spectroscopical data of (*2R*,*3R*,*5R*,*6R*)-5,6-dimethoxy-5,6-dimethyl-1,4-dioxane-2,3-dimethyl dicarboxylate (**9**)	S4
2.		Physical constants and spectroscopical data of methyl (2*S*,3*R*,5*R*,6*R*)-3-formyl-5,6-dimethoxy-5,6-dimethyl-1,4-dioxane-2-carboxylate (**15**)	S4
3.		Physical constants and spectroscopical data of [(2*S*,3*R*,5*R*,6*R*)-3-(hydroxymethyl)-5,6-dimethoxy-5,6-dimethyl-1,4-dioxan-2-yl]methanol (**17**)	S4
4.		Physical constants and spectroscopical data of (2*S*,3*R*,5*R*,6*R*)-3-*tert*-butyldimethylsilyloxymethyl-2-hydroxymethyl-5,6-dimethoxy-5,6-dimethyl-14-dioxane (**18**)	S5
5.		Physical constants and spectroscopical data of (2*R*,3*R*,5*R*,6*R*)-3-[[*tert*-butyl(dimethyl)silyl]oxymethyl]-5,6-dimethoxy-5,6-dimethyl-1,4-dioxane-2-carbaldehyde (**19**)	S5
6.		Synthesis of methyl (−)-(2*S*,3*S*,5*R*,6*R*)-3-[(*Z*)-hex-1-enyl]-5,6-dimethoxy-5,6-dimethyl-1,4-dioxane-2-carboxylate (**16**)	S6
7.		Synthesis of (+)-disparlure **1** and (+)-monachalure (**2**)	S6
	7.1.	Synthesis of (−)-*tert*-butyl-[[(2*R*,3*S*,5*R*,6*R*)-3-decyl-5,6-dimethoxy-5,6-dimethyl-1,4-dioxan-2-yl]methoxy]-dimethyl-silane (**20**)	S6
	7.2.	Synthesis of (−)-(2*S*,3*S*,5*R*,6*R*)-3-decyl-5,6-dimethoxy-5,6-dimethyl-1,4-dioxane-2-carbaldehyde (**21**)	S8
	7.3.	Synthesis of (+)-(2*R*,3*R*,5*S*,6*R*)-5-decyl-2,3-dimethoxy-2,3-dimethyl-6-(5-methylhexyl)-1,4-dioxane **(22)**	S10
	7.4.	Synthesis of (+)-(2*R*,3*R*,5*S*,6*R*)-5-decyl-6-hexyl-2,3-dimethoxy-2,3-dimethyl-1,4-dioxane (**23**)	S11
	7.5.	Synthesis of (+)-(7*R*,8*S*)-2-methyloctadecane-7,8-diol (**5**)	S13
	7.6.	Synthesis (+)-(7*R*,8*S*)-octadecane-7,8-diol (**6**)	S14
	7.7.	Synthesis of (+)-disparlure (**1**)	S15
	7.8.	Synthesis of (+)-monachalure (**2**)	S16
8.		Synthesis of (−)-disparlure (**3**) and (−)-monachalure (**4**)	S17
	8.1.	Synthesis of (−)-*tert*-butyl-[[(2*R*,3*S*,5*R*,6*R*)-5,6-dimethoxy-5,6-dimethyl-3-(5-methylhexyl)-1,4-dioxan-2-yl]methoxy]-dimethyl-silane (**24**)	S17
	8.2.	Synthesis of (−)-*tert*-butyl-[[(2*R*,3*S*,5*R*,6*R*)-3-hexyl-5,6-dimethoxy-5,6-dimethyl-1,4-dioxan-2-yl]methoxy]-dimethyl-silane (**25**)	S19

8.3. Synthesis of (−)-(2*S*,3*S*,5*R*,6*R*)-5,6-dimethoxy-5,6-dimethyl-3-(5-methylhexyl)-1,4-dioxane-2-carbaldehyde (**26**) S21

8.4. Synthesis of (−)-(2*S*,3*S*,5*R*,6*R*)-3-hexyl-5,6-dimethoxy-5,6-dimethyl-1,4-dioxane-2-carbaldehyde (**27**) S22

8.5. Synthesis of (−)-(2*R*,3*R*,5*R*,6*S*)-5-decyl-2,3-dimethoxy-2,3-dimethyl-6-(5-methylhexyl)-1,4-dioxane (**28**) S24

8.6. Synthesis of (−)-(2*R*,3*R*,5*R*,6*S*)-5-decyl-6-hexyl-2,3-dimethoxy-2,3-dimethyl-1,4-dioxane (**29**) S25

8.7. Synthesis of (−)-(7*S*,8*R*)-2-methylnonadecane-7,8-diol (**7**) S27

8.8. Synthesis of (−)-(7*S*,8*R*)-nonadecane-7,8-diol (**8**) S28

8.9. Synthesis of (−)-disparlure (**3**) S28

8.10. Synthesis of (−)-monachalure (**4**) S29

9. Determination of enantiomeric purity of (+)- and (−)-disparlure **1** and **3** via GC–MS method of *cis*-*N*-(α-methylbenzyl)aziridine derivatives S30

9.1. Derivatization of (+)-disparlure (**1**) S30

9.2. Derivatization of (−)-disparlure (**3**) S31

9.3. GC–MS method of resolution of *cis*-*N*-(α-methylbenzyl)aziridine derivatives S32

General information

All non-aqueous reactions were carried out under an atmosphere of argon using oven-dried glassware. Chemicals were purchased from Sigma–Aldrich, Merck, Fluorochem or Avantor Performance Materials Poland SA. Chemicals and solvents were of reagent grade. Tetrahydrofuran (THF) was distilled from sodium/benzophenone under argon. Diisopropylamine was dried over KOH for 24 hours and then distilled under argon. ^{1}H NMR, ^{13}C NMR spectra were obtained using a Bruker Avance 400 MHz spectrometer. ^{1}H NMR spectra were obtained at 400 MHz in $CDCl_3$ with chemical shift (δ) values in ppm downfield from tetramethylsilane, and coupling constants (*J*) are expressed in Hz. ^{13}C NMR spectra were obtained at 100.62 MHz in $CDCl_3$. Analytical TLC was done on precoated aluminum plates (Merck silica gel 60 F254 or Supelco silica gel) and visualized by UV fluorescence and aqueous solution of potassium permanganate. Preparative column chromatography was done on silica gel (60–120 mesh). Optical rotations were measured with a Jasco P-2000 polarimeter. Melting points (uncorrected) were determined on a Boetius apparatus. IR spectra were recorded as thin films between potassium bromide plates or as KBr pellets on a Nicolet 6700 spectrometer. High resolution mass spectra (HRMS) were obtained on a Bruker Daltonik micrOTOF-Q II mass spectrometer equipped with an ESI source in the positive ion mode calibrated with sodium formate clusters. Low temperature (−78 °C) was reached with a Julabo FT902 immersion cooler.

All phosphonium salts were synthesized from the corresponding alkyl bromides and triphenylphosphine according to published procedure [1]. (*2R*,*3R*,*5R*,*6R*)-5,6-Dimethoxy-5,6-dimethyl-1,4-dioxane-2,3-dimethyl dicarboxylate (**9**) was synthesized (69% yield) from dimethyl (+)-L-tartrate according to published procedures [2]. Dimethyl (+)-L-tartrate (ee ≥ 99%) was purchased from Sigma-Aldrich. Methyl (2*S*,3*R*,5*R*,6*R*)-3-formyl-5,6-dimethoxy-5,6-dimethyl-1,4-dioxane-2-carboxylate (**15**) was synthesized (78% yield) according to published procedures [3]. The overall yield of compound **15** obtained from dimethyl (+)-L-tartrate and butanone was 32%. [(2*S*,3*R*,5*R*,6*R*)-3-(Hydroxymethyl)-5,6-dimethoxy-5,6-dimethyl-1,4-dioxan-2-yl]methanol (**17**) was synthesized (in 73% yield from **14** and 83% from **15**) according to published procedures [3]. (2*R*,3*R*,5*R*,6*R*)-3-[[*tert*-Butyl(dimethyl)silyl]oxymethyl]-5,6-dimethoxy-5,6-dimethyl-1,4-dioxane-2-carbaldehyde (**19**) was synthesized in 99% yield according to published procedures [2].

1. **Physical constants and spectroscopical data of (*2R*,*3R*,*5R*,*6R*)-5,6-dimethoxy-5,6-dimethyl-1,4-dioxane-2,3-dimethyl dicarboxylate (9)**

m.p. = 107.5-108.5 °C

Lit. m.p. = 109-110 °C; [2]

$[\alpha]_D^{25}$ = -146.0 ° (c = 1.00, CH_2Cl_2);

Lit. $[\alpha]_D^{29}$ = -148.0 ° (c = 1.00, CH_2Cl_2); [2];

IR (KBr, cm^{-1}) = 2993, 1738, 1212, 14143, 1112, 1063;

^{1}H NMR (400 MHz, $CDCl_3$): δ = 4.52 (2H, s), 3.75 (6H, s), 3.31 (6H, s), 1.35 (6H, s).

^{13}C NMR (101 MHz, $CDCl_3$): δ = 168.56, 99.31, 68.86, 52.66, 48.58, 17,44.

HMRS (+ESI): *m/z* $C_{12}H_{20}$ NaO_8 $[M+Na]^+$ calcd for: 315.1050; found: 315.1041.

2. **Physical constants and spectroscopical data of methyl (2*S*,3*R*,5*R*,6*R*)-3-formyl-5,6-dimethoxy-5,6-dimethyl-1,4-dioxane-2-carboxylate (15)**

m.p. = 75.2-75.9 °C;

$[\alpha]_D^{20}$ = -128.5 ° (c = 1.00, $CHCl_3$);

IR (KBr, cm^{-1}): 2839, 1740, 1722;

^{1}H NMR (400 MHz, $CDCl_3$): δ = 9.69 (1H, s), 4.56 (1H, d, *J* = 4.4 Hz), 4.43 (1H, d, *J* = 4.4 Hz), 3.77 (3H, s), 3.31 (3H, s), 3.21 (3H, s), 1.40 (3H, s), 1.36 (3H, s).

^{13}C NMR (101 MHz, $CDCl_3$): δ = 199.25, 170.0, 100.51, 99.95, 72.49, 70.34, 52.44, 49.83, 48.60, 18.21, 17.87.

HRMS (+ESI): *m/z* $C_{11}H_{18}NaO_7$ $[M+Na]^+$ calcd for: 285.0945; found: 285.0948.

3. **Physical constants and spectroscopical data of [(2*S*,3*R*,5*R*,6*R*)-3-(hydroxymethyl)-5,6-dimethoxy-5,6-dimethyl-1,4-dioxan-2-yl]methanol (17)**

m.p. = 69 °C;

Lit. m.p. = 69 °C; [2]

$[\alpha]_D^{20}$ = -132.1 ° (c = 1.00, CH_2Cl_2);

Lit. $[\alpha]_D^{29}$ = -132.0 ° (c = 1.00, CH_2Cl_2); [2]

IR (KBr, cm^{-1}): 3273 br, 2950 m, 2834 m, 1121 s, 1138 s;

^{1}H NMR (400 MHz, $CDCl_3$): δ = 4.25-4.21(1H, m), 4.05-4.00 (1H, m), 3.92-3.90 (1H, m, OH), 3.89-3.67 (4H, m, OH’), 3.34 (3H, s), 3.25 (3H, s), 3.14 (1H, t, *J* = 6.2 Hz), 1.33 (3H, s), 1.31 (3H, s).

^{13}C NMR (101 MHz, $CDCl_3$): δ = 99.6, 98.1, 72.8, 68.4, 63.2, 61.7, 49.4, 48.1, 18.3, 18.0.

HRMS (+ESI): *m/z* $C_{10}H_{20}NaO_6$ $[M+Na]^+$ calcd for: 259.1152; found: 259.1189.

4. **Physical constants and spectroscopical data of (2*S*,3*R*,5*R*,6*R*)-3-*tert*-butyldimethylsilyloxymethyl-2-hydroxymethyl-5,6-dimethoxy-5,6-dimethyl-14-dioxane (18)**

m.p. = 88 °C;

Lit. m.p. . = +79 °C;[2]

$[\alpha]_D^{20}$ = -116.6 ° (c = 1.00, $CHCl_3$);

Lit. $[\alpha]_D^{29}$ = -119.0 ° (c = 1.05, $CHCl_3$);[2]

IR (KBr, cm^{-1}): 3490 bs, 2999 m, 2955 m, 2885 m, 2856 m, 1139 s, 1121 s;

^1H NMR (400 MHz, $CDCl_3$): δ = 4.38 (1H, d, *J* = 9.9 Hz), 4.25 (1H, td, *J* = 6.7 Hz, 3.9 Hz), 3.76 (1H, ddd, *J* = 9.9 Hz, 3.8 Hz, 2.7 Hz,), 3.68 (2H, dd, *J* = 7.0 Hz, 3.1 Hz), 3.59 (1H, ddd, *J* = 9.9 Hz, 2.6 Hz, 1.0 Hz,), 3.26 (3H, s), 3.24 (3H, s), 2.97 (1H, t, *J* = 7.5 Hz), 1.28 (3H, s), 1.27 (3H, s), 0.89 (9H, s), 0.10 (6H, s).

^{13}C NMR (101 MHz, $CDCl_3$): δ = 99.40, 98.28, 72.93, 68.00, 62.49, 61.81, 49.49, 48.09, 25.95, 18.30, 18.23, 18.00, -5.37, -5.40.

HRMS (+ESI): *m/z* calcd for $C_{16}H_{34}NaO_6Si$ $[M+Na]^+$: 373.2017; found: 373.2027.

5. **Physical constants and spectroscopical data of (2*R*,3*R*,5*R*,6*R*)-3-[[*tert*-butyl(dimethyl)silyl]oxymethyl]-5,6-dimethoxy-5,6-dimethyl-1,4-dioxane-2-carbaldehyde (19)**

m.p. = 47 °C;

Lit. m.p. = 50 °C; [2]

$[\alpha]_D^{20}$ = -123.5 ° (c = 1.00, $CHCl_3$);

Lit. $[\alpha]_D^{28}$ = -122.0 ° (c = 1.08, $CHCl_3$); [2]

IR (KBr, cm^{-1}): 2952 m, 2952 m, 2858 m, 1738 s;

^1H NMR (400 MHz, $CDCl_3$): δ = 9.50 (1H, s), 4.53 (1H, d, *J* = 3.9 Hz), 4.32 (1H, d, *J* = 9.7 Hz), 4.16-4.09 (1H, m), 3.62 (1H, dd, *J* = 4.1 Hz, J = 9.6 Hz), 3.25 (3H, s), 3.19 (3H, s), 1.37 (3H, s), 1.27 (3H, s), 0.85 (9H, s), 0.02 (6H, s).

^{13}C NMR (101 MHz, $CDCl_3$): δ = 195.81, 100.55, 98.72, 74.49, 72.19, 61.18, 48.85, 45.38, 25.91, 18.53, 18.07, -5.48, -5.41.

HRMS (+ESI): *m/z* $C_{16}H_{32}NaO_6Si$ $[M+Na]^+$ calcd for: 371.1860; found: 371.1859.

6. Synthesis of methyl (-)-(2*S*,3*S*,5*R*,6*R*)-3-[(*Z*)-hex-1-enyl]-5,6-dimethoxy-5,6-dimethyl-1,4-dioxane-2-carboxylate (16)

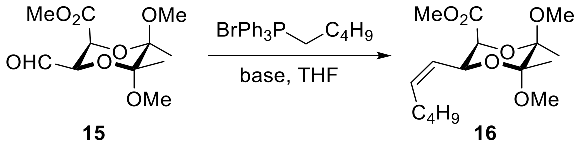

15 16

LiHMDS (1.0 M in THF, 1.31 mL, 1.31 mmol) was added dropwise to a mixture of *n*-pentyltriphenylphosphonium bromide (0.539 g, 1.31 mmol) in anhydrous THF (8.0 mL) at −30 °C. The mixture was stirred for 30 min and cooled to −78 °C. Next, methyl (2*S*,3*R*,5*R*,6*R*)-3-formyl-5,6-dimethoxy-5,6-dimethyl-1,4-dioxane-2-carboxylate (**15**, 0.163 g, 0.621 mmol) in anhydrous THF (3 mL) was added at −78 °C. The cooling was turned off and the mixture stirred until the temperature reached 0 °C (about 1 hour), and TLC analysis (eluent: 20% ethyl acetate in hexane) showed complete conversion of the substrate. The reaction mixture was filtered through a thin layer of silica gel, which was washed with ethyl acetate. The collected organic layer was washed with saturated aqueous ammonium chloride, water, brine and dried over magnesium sulfate. The crude reaction mixture after removing the organic solvent was purified by column chromatography (15 g silica gel, eluent: 10% ethyl acetate in hexane) giving product **16** as an oil (0.101 g, 0.316 mmol, 51% yield).
$[\alpha]_D^{20}$ = -102.5 ° (c = 1.00, $CHCl_3$);
^{1}H NMR (400 MHz, $CDCl_3$): δ = 5.90 (1H, ddt, *J* = 11.3 Hz, 8.4 Hz, 1.5 Hz), 5.55 (1H, dddd, *J* = 11.1 Hz, 8.2 Hz, 7.0 Hz, 1.2 Hz), 4.97 (1H, ddd, *J* = 8.4 Hz, 3.9 Hz, 1.3 Hz), 3.99 (1H, d, *J* = 3.9 Hz), 3.75 (3H, s), 3.28 (3H, s), 3.23 (3H, s), 2.18-1.92 (2H, m), 1.36-1.26 (10H, m), 0.87 (3H, t, *J* = 7.1 Hz).

7. Synthesis of (+)-disparlure (1) and (+)-monachalure (2)

7.1. Synthesis of (−)-*tert*-butyl-[[(2*R*,3*S*,5*R*,6*R*)-3-decyl-5,6-dimethoxy-5,6-dimethyl-1,4-dioxan-2-yl]methoxy]-dimethylsilane (20)

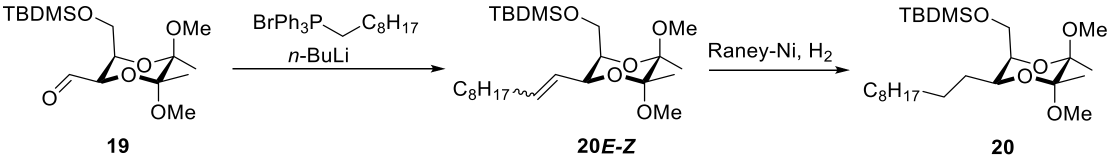

19 20*E-Z* 20

n-Nonyltriphenylphosphonium bromide (7.32 g, 15.6 mmol) was dried in a Schlenk flask at 60 °C under reduced pressure (10 mbar) for 1 hour. Next, anhydrous THF

(80 mL) was added, followed by cooling to −25 °C and addition of *n*-butyllithium (2.5 M in hexane, 5.83 mL, 14.6 mmol). After 30 min (2*R*,3*R*,5*R*,6*R*)-3-[[*tert*-butyl(dimethyl)silyl]oxymethyl]-5,6-dimethoxy-5,6-dimethyl-1,4-dioxane-2-carbaldehyde (**19**, 2.82 g, 8.10 mmol) in anhydrous THF (8 mL) was added at −78 °C. The cooling was turned off and the mixture stirred until it reached 0 °C (about 1 hour), and TLC analysis (eluent: 20% ethyl acetate in hexane) showed complete conversion of the substrate. Next, diethyl ether (20 mL) and water (20 mL) were added. The aqueous layer was extracted twice with 15 mL of diethyl ether and the combined organic layers were washed with brine, water and dried over magnesium sulfate. The crude reaction mixture, after removing the organic solvents, was purified by column chromatography (50 g silica gel, eluent: 2% ethyl acetate in hexane) giving olefin **20*E-Z*** as an oil (3.07 g, 6.68 mmol, 82% yield) as a mixture of geometric isomers in a ratio 1:0.2.

$[\alpha]_D^{20}$ = -68.2 ° (c = 1.00, $CHCl_3$);

IR (Film, cm^{-1}): 2955 s, 2928 s, 2856 s, 1463 m, 1373 m, 1256 m, 1137 s, 1114 s, 1051 b m;

^1H NMR (400 MHz, $CDCl_3$): δ = 5.63 (1H from isomer *E* or *Z*, dt, *J* = 10.8 Hz, 7.4 Hz), 5.56-5.49 (1H from isomer *E* or *Z*, m), 5.42 (1H from isomer *E* or *Z*, dd, *J* = 10.8 Hz, 9.2 Hz), 5.29-5.21 (1H from isomer *E* or *Z*, m), 4.94 (1H from isomer *E* or *Z*, dd, *J* = 8.9 Hz, 4.3 Hz), 4.82 (1H from isomer *E* or *Z*, t, *J* = 9.8 Hz), 4.51 (1H from isomer *E* or *Z*, dd, *J* = 7.6 Hz, 4.1 Hz), 4.05 (1H from isomer *E* or *Z*, dd, *J* = 10.6 Hz, 6.8 Hz), 3.85 (1H from isomer *E* or *Z*, dd, *J* = 10.5 Hz, 5.8 Hz), 3.71-3.7 (1H from isomer *E* or *Z*, m), 3.65-3.60 (1H from isomer *E* or *Z*, m), 3.55-3.52 (1H from isomer *E* or *Z*, m), 3.31(6H from isomer *E* and *Z*, s), 3.27 (6H from isomer *E* and *Z*, s), 2.15-2.01 (4H from isomer *E* and *Z*, m), 1.39-1.22 (36H from isomer *E* and *Z*, m), 0.91-0.84 (24H from isomer *E* and *Z*, m), 0,03 (12H from isomer *E* and *Z*, d, *J* = 2.1 Hz).

^{13}C NMR (101 MHz, $CDCl_3$); δ = 133.60, 125.82, 99.81, 98.03, 75.42, 65.03, 62.24, 49.52, 48.12, 32.02, 32.1, 29.85, 29.64, 29.44, 29.39, 28.13, 26.12, 25.98, 22.81, 18.65, 18.23, 14.25, -5.19, -5.21.

HRMS (+ESI): *m/z* calcd for $C_{25}H_{50}NaO_5Si$ $[M+Na]^+$: 481.3320; found: 481.3234.

Raney nickel (9.20 g, 50% in water) was washed 10 times with anhydrous methanol (10 mL), suspended in 10 mL of methanol and hydrogen was bubbled through the suspension. Next the olefin **20*E-Z*** (3.07 g, 6.68 mmol) in methanol (10 mL) was

added to the mixture and a balloon filled with hydrogen was attached to the flask through a rubber septum. The reaction was vigourously stirred until TLC analysis (eluent: 5% ethyl acetate in hexane) showed complete conversion of the substrate (16 hours). Next, the flask was filled with argon, vigourously stirred for 5 min. The reaction mixture was filtered through a thin layer of silica gel, which was washed with diethyl ether. The crude reaction mixture, after removing the organic solvent, was purified by column chromatography (45 g silica gel, eluent: 2% ethyl acetate in hexane) giving (−)-*tert*-butyl-[[(2*R*,3*S*,5*R*,6*R*)-3-decyl-5,6-dimethoxy-5,6-dimethyl-1,4-dioxan-2-yl]methoxy]-dimethylsilane (**20**) as an oil (2.78 g, 6.03 mmol, 90% yield).

$[\alpha]_D^{20}$ = -82.2 ° (c = 1.00, $CHCl_3$);

IR (Film, cm^{-1}): 2954 s, 2927 s, 2856 s, 1463 m, 1374 m, 1256 m, 1138 s, 1115 s, 1040 s, 837 s;

^1H NMR (400 MHz, $CDCl_3$): δ = 4.13 (1H, dt, *J* = 7.1 Hz, 5.6 Hz), 4.07-4.00 (1H, m), 3.68 (1H, dd, *J* = 10.2 Hz, 3.7 Hz), 3.58 (1H, dt, *J* = 7.8 Hz, 3.5 Hz), 3.26 (3H, s), 3.24 (3H, s), 1.55-1.40 (2H, m), 1.32-1.22 (22H, m), 0.90-0.84 (12H, m), 0.04 (6H, s).

^{13}C NMR (101 MHz, $CDCl_3$): δ = 99.61, 98.16, 75.42, 68.72, 62.41, 49.52, 47.91, 32.06, 30.92, 29.92, 29.82, 29.77, 29.75, 29.46, 26.34, 26.00, 22.84, 18.45, 18.29, 18.25, 14.27, -5.24, -5.29,

HRMS (+ESI): *m/z* calcd for $C_{25}H_{52}NaO_5Si$ $[M+Na]^+$: 483.3476; found: 483.3398.

7.2. Synthesis of (-)-(2*S*,3*S*,5*R*,6*R*)-3-decyl-5,6-dimethoxy-5,6-dimethyl-1,4-dioxane-2-carbaldehyde (21)

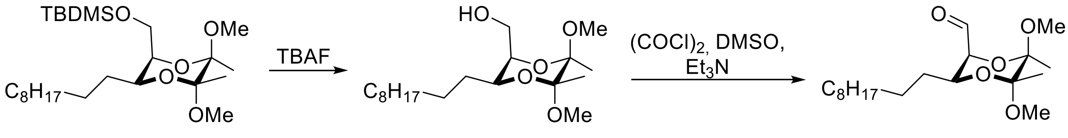

20 **20a** **21**

Tetra-*n*-butylammonium fluoride (1 M in THF, 8.87 mL, 8,87 mmol) was added to (−)-*tert*-butyl-[[(2*R*,3*S*,5*R*,6*R*)-3-decyl-5,6-dimethoxy-5,6-dimethyl-1,4-dioxan-2-yl]methoxy]-dimethylsilane (**20**, 2.73 g, 5.92 mmol) at room temperature. The reaction mixture was stirred until TLC analysis (eluent: 5% ethyl acetate in hexane) showed complete conversion of the substrate (5 hours). Next, saturated sodium hydrogencarbonate (10 mL) was added. The aqueous layer was extracted twice with 10 mL of ethyl acetate and the combined organic layers were washed with brine, water and dried over magnesium sulfate. The crude reaction mixture, after removing

the organic solvents, was purified by column chromatography (45 g silica gel, eluent: 15% ethyl acetate in hexane) giving alcohol **20a** as an oil (2.03 g, 5.86 mmol, 99% yield).

$[\alpha]_D^{20}$ = -80.8 ° (c = 1.00, $CHCl_3$);

IR (Film, cm^{-1}): 3471 bm, 2925 s, 2855 s, 1462 m, 1375 s, 1138 s, 1118 s, 1038 s;

^1H NMR (400 MHz, $CDCl_3$): δ = 4.07 (1H, dt, *J* = 8.7 Hz, 4.1 Hz), 3.87-3.75 (3H, m), 3.57 (1H, td, *J* = 4.0 Hz, 2.1 Hz,), 3.39 (3H, s), 3.24 (3H, s), 1.77 (1H, ddd, *J* = 13.6 Hz, 9.7 Hz, 5.0 Hz), 1.59-1.42 (2H, m), 1.45-1.19 (20H, m), 0.92-0.81 (4H, m).

^{13}C NMR (101 MHz, $CDCl_3$): δ = 99.46, 97.98, 74.36, 68.43, 63.39, 49.29, 47.95, 32.05, 30.42, 29.77, 29.73, 29.45, 26.26, 22.82, 18.44, 18.20, 14.24.

HRMS (+ESI): *m/z* calcd for $C_{19}H_{38}NaO_5$ $[M+Na]^+$: 369.2611; found: 369.2558.

DMSO (0.198 mL, 2.79 mmol) was added dropwise to the mixture of oxalyl chloride (0.147 mL, 1.74 mmol) in anhydrous dichloromethane (10 mL) at −60 °C. After 5 min of stirring, alcohol **20a** (1.40 g, 1.16 mmol) in anhydrous dichloromethane (4 mL) was added. The reaction was kept for 15 min at −60 °C followed by the addition of triethylamine (0.485 mL, 3.48 mmol). After the reaction mixture had warmed to room temperature, water (10 mL) was added. The aqueous layer was extracted twice with 10 mL of dichloromethane and the combined organic layers were washed with saturated sodium hydrogencarbonate and dried over magnesium sulfate. The crude product (0.421 g) was used in the next step without further purification.

$[\alpha]_D^{20}$ = -51.7 ° (c = 1.00, $CHCl_3$);

IR (Film, cm^{-1}): 2926 s, 2855 s, 1731 s, 1467 m, 1379 m, 1116 s, 1046 s;

^1H NMR (400 MHz, $CDCl_3$): δ = 9.96 (1H, dd, *J* = 2.9 Hz, 0.7 Hz), 4.12-4.06 (1H, m). 3.58-3.55 (1H, m), 3.27 (3H, s), 3.17 (3H, s), 1.72-1.64 (1H, m), 1.55-1.44 (2H, m) 1.36-1.18 (21H, m), 0.87 (3H, t, *J* = 6.8 Hz).

^{13}C NMR (101 MHz, $CDCl_3$); δ = 200.31, 99.25, 98.40, 75.66, 66.90, 48.74, 47.59, 31.47, 29.26, 29.15, 29.03, 28.88, 25.46, 22.26, 17.58, 16.99, 13.70.

HRMS (+ESI): *m/z* calcd for $C_{19}H_{36}NaO_5$ $[M+Na]^+$: 367.2455; found: 367.2502.

7.3. Synthesis of (+)-(2*R*,3*R*,5*S*,6*R*)-5-decyl-2,3-dimethoxy-2,3-dimethyl-6-(5-methylhexyl)-1,4-dioxane (22)

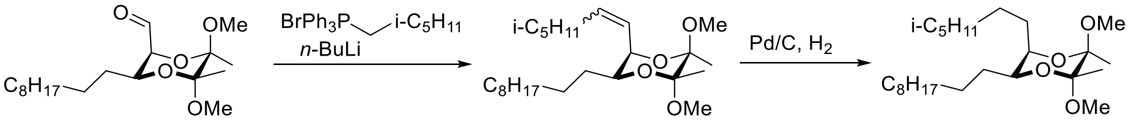

21 **22*E-Z*** **22**

4-Methylpentyltriphenylphosphonium bromide (0.957 g, 2.21 mmol) was dried in a Schlenk flask at 60 °C under reduced pressure (10 mbar) for 1 hour. Next, anhydrous THF (30 mL) was added, followed by cooling to −25 °C and the addition of *n*-butyllithium (2.5 M in hexane, 0.850 mL, 2.13 mmol). After 30 min, (−)-(2*S*,3*S*,5*R*,6*R*)-3-decyl-5,6-dimethoxy-5,6-dimethyl-1,4-dioxane-2-carbaldehyde (**21**, 0.421 g, 1.16 mmol) in anhydrous THF (5 mL) was added at −78 °C. The cooling was turned off and the mixture stirred until the temperature reached 0 °C (about 1 hour), and TLC analysis (eluent: 20% ethyl acetate in hexane) showed complete conversion of the substrate. Next, diethyl ether (20 mL) and water (20 mL) were added. The aqueous layer was extracted twice with 15 mL of diethyl ether and the combined organic layers were washed with brine, water and dried over magnesium sulfate. The crude reaction mixture after removing the organic solvents was purified by column chromatography (20 g silica gel, eluent: 2% ethyl acetate in hexane) giving the olefin **22*E-Z*** as an oil (0.301 g, 0.731 mmol, 63% yield) as a mixture of geometric isomers in a ratio 1:0.2.

$[\alpha]_D^{20}$ = -42.8 ° (c = 1.00, $CHCl_3$);

IR (Film, cm^{-1}): 2955 m, 2926 s, 2855 m, 1467 m, 1374 m, 1139 s, 1115 s, 1040 s, 758 s;

^1H NMR (400 MHz, $CDCl_3$): δ = 6.00 (1H from isomer *E* or *Z*, dd, *J* = 15.4 Hz, 10.2 Hz), 5.95-5.87 (1H isomer *E* or *Z*, m), 5.68-5.62 (1H isomer *E* or *Z*, m), 5.59 (1H from isomer *E* or *Z*, ddd, *J* = 14.7 Hz, 8.9 Hz, 4.7 Hz), 4.28 (1H from isomer *E* or *Z*, dd, *J* = 10.3 Hz, 3.9 Hz), 4.10-4.03 (1H from isomer *E* or *Z*, m), 4.01 (1H from isomer *E* or *Z*, dd, *J* = 8.4 Hz, 4.5 Hz), 3.83 (1H from isomer *E* or *Z*, dd, *J* = 10.2 Hz, 3.9 Hz), 3.25 (6H from isomer *E* and *Z*, s), 3.17 (3H from isomer *E* or *Z*, s), 3.12 (3H from isomer *E* or *Z*, s), 2.13-2.02 (4H from isomer *E* and *Z*, m), 1.55 (2H from isomer *E* and *Z*, m), 1.48-1.15 (52H from isomer *E* and *Z*, m), 0.91-0.83 (18H from isomer *E* and *Z*, m).

^{13}C NMR (101 MHz, $CDCl_3$): δ = 134.34, 126.21, 99.62, 98.14, 68.92, 68.65, 48.80, 47.92, 39.01, 32.05, 31.12, 29.79, 29.73, 29.68, 29.46, 27.80, 25.56, 25.16, 22.83, 22.68, 22.54, 18.64, 18.11, 14.26.

HRMS (+ESI): *m/z* calcd for $C_{25}H_{48}NaO_4$ $[M+Na]^+$: 435.3445; found: 435.3398.

The solution of olefin **22*E-Z*** (0.418 g, 1.01 mmol) in anhydrous THF (5 mL) was placed in a round-bottomed flask which was filled with argon followed by addition of Pd/C (10 mol %, 0.128 g, 10%). The flask was closed with a rubber septum and a balloon filled with hydrogen was attached. The mixture was vigorously stirred until TLC analysis (eluent: 5% ethyl acetate in hexane) showed complete conversion of the substrate (18 hours). Next, the flask was filled with argon, and the mixture vigorously stirred for 5 min. The reaction mixture was filtered through a thin layer of Celite, which was washed with ethyl acetate. The crude reaction mixture, after removing the organic solvent, was purified by column chromatography (13 g silica gel, eluent: 2.5% ethyl acetate in hexane) giving (+)-(2*R*,3*R*,5*S*,6*R*)-5-decyl-2,3-dimethoxy-2,3-dimethyl-6-(5-methylhexyl)-1,4-dioxane (**22**) as an oil (0.404 g, 0.973 mmol, 96% yield).

$[\alpha]_D^{20}$ = +2.5 ° (c = 1.00, $CHCl_3$);

IR (Film, cm^{-1}): 3321 bm, 3245 bm, 2955 m, 2919 m, 2851 s, 1469 s, 1374s, 1137 s, 1112 s;

^1H NMR (400 MHz, $CDCl_3$): δ = 4.06 (1H, dt, J = 8.6 Hz, 4.2 Hz), 3.52-3.42 (1H, m), 3.28 (3H, s), 3,23 (3H, s), 1.99-1.85 (1H, m), 1.64-1.12 (32H, m), 0.96-0.77 (9H, m).

^{13}C NMR (101 MHz, $CDCl_3$): δ = 99.78, 98.76, 75.03, 68.99, 49.40, 47.89, 39.17, 32.05, 31.53, 29.84, 29.76, 29.74, 29.70, 29.46, 28.75, 28.06, 27.75, 27.69, 26.14, 22.82, 22.73, 19.00, 18.33, 14.26.

HRMS (+ESI): *m/z* calcd for $C_{25}H_{50}NaO_4$ $[M+Na]^+$: 437.3601; found: 437.3506

7.4. Synthesis of (+)-(2*R*,3*R*,5*S*,6*R*)-5-decyl-6-hexyl-2,3-dimethoxy-2,3-dimethyl-1,4-dioxane (23)

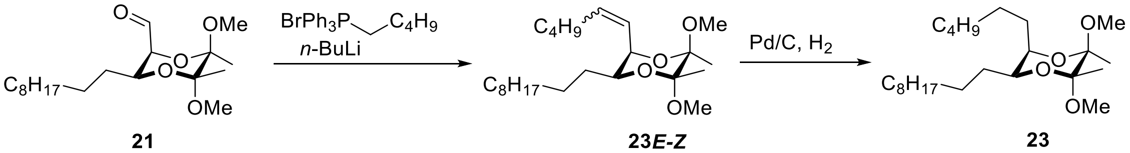

21 **23*E-Z*** **23**

n-Pentyltriphenylphosphonium bromide (0.835 g, 2.02 mmol) was dried in a Schlenk flask at 60 °C under reduced pressure (10 mbar) for 1 hour. Next, anhydrous THF (20 mL) was added, followed by cooling to −25 °C and addition of *n*-butyllithium (2.5 M in hexane, 0.808 mL, 2.02 mmol). After 30 min, (−)-(2*S*,3*S*,5*R*,6*R*)-3-decyl-5,6-dimethoxy-5,6-dimethyl-1,4-dioxane-2-carbaldehyde (**21**, 0.365 g, 1.01 mmol) in anhydrous THF (4 mL) was added at −78 °C. The cooling was turned off and the mixture was stirred until the temperature reached 0 °C (about 1 hour), and TLC analysis (eluent: 20% ethyl acetate in hexane) showed complete conversion of the substrate. Next, diethyl ether (20 mL) and water (20 mL) were added. The aqueous layer was extracted twice with 15 mL of diethyl ether and the combined organic layers were washed with brine, water and dried over magnesium sulfate. The crude reaction mixture, after removing the organic solvents, was purified by column chromatography (20 g silica gel, eluent: 2% ethyl acetate in hexane) giving olefin **23*E-Z*** as an oil (0.285 g, 0.717 mmol, 71% yield) as a mixture of geometric isomers in a ratio 1:0.2.

$[\alpha]_D^{20}$ = -43.5 ° (c = 1.00, $CHCl_3$);

IR (Film, cm^{-1}): 2955 s, 2926 s, 2855 s, 1467 m, 1374 m, 1215 m, 1139 s, 1115 s, 1040 s, 758 s;

^{1}H NMR (400 MHz, $CDCl_3$): δ = 6.04-5.97 (1H from isomer *E* or *Z*, m), 5.96-5.88 (1H from isomer *E* or *Z*, m), 5.64-5.54 (1H from isomer *E* or *Z*, m), 5.35-5.18 (1H from isomer *E* or *Z*, m), 4.27 (1H from isomer *E* or *Z*, ddd, *J* = 10.3 Hz, 3.9 Hz, 0.8 Hz), 4.11-4.03 (1H from isomer *E* or *Z*, m), 4.03-3.98 (1H from isomer *E* or *Z*, m), 3.83 (1H from isomer *E* or *Z*, dd, *J* = 10.2 Hz, 3.9 Hz), 3.25 (6H from isomer *E* and *Z*, s), 3.17 (3H from isomer *E* or *Z*, s), 3.13 (3H from isomer *E* or *Z*, s), 2.28-2.19 (2H from isomer *E* or *Z*, m), 2.16-2.00 (2H from isomer *E* or *Z*, m), 1.45-1.15 (56H from isomer *E* and *Z* m), 0.96-0.83 (12H from isomer *E* or *Z*, m).

^{13}C NMR (101 MHz, $CDCl_3$): δ = 134.23, 126.35, 99.63, 98.14, 68.94, 68.67, 48.79, 47.91, 32.05, 31.99, 31.13, 29.79, 29.73, 29.67, 29.46, 26.95, 25.57, 22.83, 22.53, 18.63, 18.12, 14.26, 14.07.

HRMS (+ESI): *m/z* calcd for $C_{24}H_{46}NaO_4$ $[M+Na]^+$: 421.3289; found: 421.3240.

The solution of olefin **23*E-Z*** (0.281 g, 0.701 mmol) in methanol (5 mL) was placed in a round-bottomed flask which was filled with argon followed by the addition of Pd/C (10 mol %, 0.100 g, 10%). The flask was closed with a rubber septum and a balloon

filled with hydrogen was attached. The mixture was vigorously stirred until TLC analysis (eluent: 5% ethyl acetate in hexane) showed complete conversion of the substrate (18 hours). Next, the flask was filled with argon, and the mixture vigorously stirred for 5 min. The reaction mixture was filtered through a thin layer of Celite, which was washed with ethyl acetate. The crude reaction mixture, after removing the organic solvents, was purified by column chromatography (13 g silica gel, eluent: 5% ethyl acetate in hexane) giving (+)-(2*R*,3*R*,5*S*,6*R*)-5-decyl-6-hexyl-2,3-dimethoxy-2,3-dimethyl-1,4-dioxane (**23**) as an oil (0.277 g, 0.692 mmol, 98% yield).

$[\alpha]_D^{20}$ = +2.5 ° (c = 1.00, $CHCl_3$);

IR (Film, cm^{-1}): 2953 s, 2922 s, 2854 s, 1468 s, 1374 s, 1137 s, 1113 s, 1040 s;

^1H NMR (400 MHz, $CDCl_3$): δ = 4.05 (1H, dt, *J* = 8.6 Hz, 4.2 Hz), 3.50-3.43 (1H, m), 3.28 (3H, s), 3.23 (3H, s), 1.97-1.85 (1H, m), 1.67-1.18 (33H, m), 0.87 (6H, t, *J* = 6.6 Hz).

^{13}C NMR (101 MHz, $CDCl_3$): δ = 99.78, 98.76, 75.05, 68.99, 49.39, 47.89, 32.06, 31.53, 29.84, 29.76, 29.74, 29.69, 29.65, 29.46, 28.74, 27.51, 26.14, 22.82, 19.00, 18.33, 14.26, 14.24.

HRMS (+ESI): *m/z* calcd for $C_{24}H_{48}NaO_4$ $[M+Na]^+$: 423.3445; found: 423.3355.

7.5. Synthesis of (+)-(7*R*,8*S*)-2-methyloctadecane-7,8-diol (5)

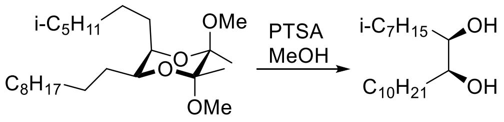

22 **5**

p-Toluenesulfonic acid (0.270 g, 1.42 mmol) was added to (+)-(2*R*,3*R*,5*S*,6*R*)-5-decyl-2,3-dimethoxy-2,3-dimethyl-6-(5-methylhexyl)-1,4-dioxane (**22**, 0.380 g, 0.917 mmol) in anhydrous methanol (15 mL). The reaction mixture was stirred at 60 °C under an Ar atmosphere until TLC analysis (eluent: 5% ethyl acetate in hexane) showed complete conversion of the substrate **22** (1 hour). Next, after cooling the reaction mixture to room temperature, anhydrous triethylamine (0.198 mL, 1.42 mmol) was added dropwise and the solvent evaporated. The crude mixture was purified by column chromatography (13 g silica gel, eluent: 20% ethyl acetate in hexane) giving (+)-(7*R*,8*S*)-2-methyloctadecane-7,8-diol (**5**) as a solid (0.219 g, 0.728 mmol, 79% yield).

m.p. = 87.7 °C;

Lit. m.p. = 86-87 °C [4]; Lit. m.p. = 85-88 °C [5];

$[\alpha]_D^{20}$ = +1.8° (c = 1.00, $CHCl_3$);

Lit. $[\alpha]_D^{22}$ = 1.9° (c = 0.5, $CHCl_3$) [4];

IR (KBr, cm^{-1}): 3328 bs, 3260 bs, 2916 s, 2916 m, 2874 m, 2850 m, 1470 s, 1069 s;

^1H NMR (400 MHz, $CDCl_3$): δ = 3.62-3.58 (2H, m), 2.01 (2H, br s), 1.54-1.16 (27H, m), 0.90-0.86 (9H, m).

^{13}C NMR (101 MHz, $CDCl_3$) δ = 74.83, 39.05, 32.05, 31.32, 31.30, 29.83, 29.76, 29.74, 29.48, 28.07, 27.57, 26.42, 26.18, 22.83, 22.78, 22.75, 14.27.

HRMS (+ESI): *m/z* calcd for $C_{19}H_{40}NaO_2$ $[M+Na]^+$: 323.2921; found: 323.2870.

7.6. Synthesis (+)-(7*R*,8*S*)-octadecane-7,8-diol (6)

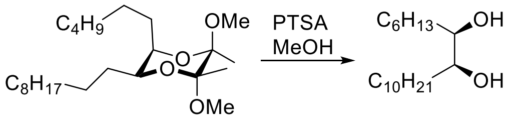

23 **6**

p-Toluenesulfonic acid (0.179 g, 0.944 mmol) was added to (+)-(2*R*,3*R*,5*S*,6*R*)-5-decyl-6-hexyl-2,3-dimethoxy-2,3-dimethyl-1,4-dioxane (**23**, 0.252 g, 0.629 mmol) in anhydrous methanol (15 mL). The reaction mixture was stirred at 60 °C under an Ar atmosphere until TLC analysis (eluent: 5% ethyl acetate in hexane) showed complete conversion of the substrate **23** (1 hour). Next, after cooling the reaction mixture to room temperature, anhydrous triethylamine (0.132 mL, 0.944 mmol) was added dropwise and the solvent was evaporated. The crude mixture was purified by column chromatography (13 g silica gel, eluent: 20% ethyl acetate in hexane) giving (+)-(7*R*,8*S*)-octadecane-7,8-diol (**6**) as a solid (0.116 g, 0.405 mmol, 64% yield).

m.p. = 119.7 °C

$[\alpha]_D^{20}$ = +0.31° (c = 1.00, $CHCl_3$);

IR (KBr, cm^{-1}): 3290 b s, 3213 b s, 2954 s, 2937 s, 2916 s, 2849 s, 1469 s, 1378 w, 1069 s, 1036 m;

^1H NMR (400 MHz, $CDCl_3$): δ = 3.60 (2H, br s), 1.85 (2H, br s), 1.58-1.17 (28H, m), 0.94-0.78 (6H, m).

^{13}C NMR (101 MHz, $CDCl_3$): δ = 74.85, 32.06, 31.94, 31.35, 29.83, 29.76, 29.74, 29.49, 26.16, 26.13, 22.83, 22.77, 14.26, 14.22.

HRMS (+ESI): *m/z* calcd for $C_{18}H_{38}NaO_2$ $[M+Na]^+$: 309.2764; found: 309.2745.

7.7. Synthesis of (+)-disparlure (1)

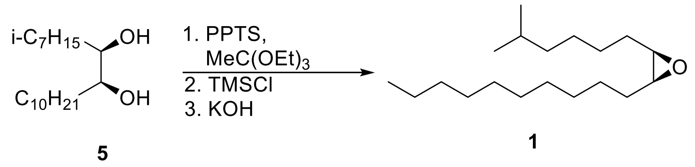

Triethyl orthoacetate (0.240 mL, 1.25 mmol) and pyridinium *p*-toluenesulfonate (2 mg) were added to (+)-(7*R*,8*S*)-2-methyloctadecane-7,8-diol (**5**, 0.075 g, 0.250 mmol) in anhydrous toluene (1.5 mL). The reaction mixture was stirred at 110 °C under an Ar atmosphere (2 hours). Next, after cooling the reaction mixture to room temperature, the solvent was evaporated and the residue dissolved in anhydrous dichloromethane (2 mL) followed by the addition of trimethylsilyl chloride (0.317 mL, 2.50 mmol). The reaction mixture was stirred at room temperature under an Ar atmosphere for 15 hours. After removing the solvent, the product was purified by flash column chromatography (5 g silica gel, eluent: 2% ethyl acetate in hexane) and dissolved in anhydrous THF (1.5 mL). A solution of potassium hydroxide (1 N in methanol, 0.720 mL, 0.720 mmol) in anhydrous THF (1.5 mL) was added at room temperature and the reaction mixture was stirred for 2 hours. Next, water (10 mL) was added and the mixture extracted twice with diethyl ether. The combined organic layers were washed with brine and dried over magnesium sulfate. The crude mixture was purified by column chromatography (6 g silica gel, eluent: 2% ethyl acetate in hexane) giving (+)-disparlure (**1**) as an oil (0.062 g, 0.218 mmol, 87% yield).

$[\alpha]_D^{20}$ = +1.1° (c = 1.00, CCl_4);

Lit. $[\alpha]_D^{25}$ = +0.8° (c = 0.50, CCl_4) [4]; Lit. $[\alpha]_D$ = +0.9 ° (c = 1.10, CCl_4) [6];

Lit. $[\alpha]_D^{25}$ = +1.60 ° (c = 1.10, CCl_4) [7]; Lit. $[\alpha]_D$ = +1.0 ° (c = 1.50 CCl_4) [8];

IR (Film, cm^{-1}): 2955m, 2925 s, 2855 m, 1467 s/m, 1384 s/m 1366 s/m;

^{1}H NMR (400 MHz, $CDCl_3$): δ = 2.91-2.88 (2H, m), 1.56-1.14 (27H, m), 0.89-0.85 (9H, m).

^{13}C NMR (101 MHz, $CDCl_3$) δ = 57.40, 39.05, 32.05, 29.75, 29.71, 29.48, 28.04, 28.01, 27.98, 27.48, 27.01, 26.75, 22.83, 22.76, 14.26.

HRMS (+ESI): *m/z* calcd for $C_{19}H_{38}O$ $[M+Na]^+$: 305.2815; found: 305.2838

7.8. Synthesis of (+)-monachalure (2)

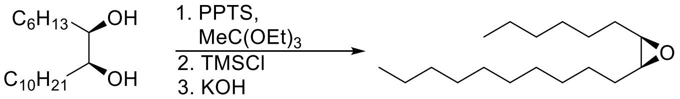

6

2

Triethyl orthoacetate (0.320 mL, 1.25 mmol) and pyridinium *p*-toluenesulfonate (3 mg) were added to (+)-(7*R*,8*S*)-octadecane-7,8-diol (**6**, 0.100 g, 0.350 mmol) in anhydrous toluene (1.5 mL). The reaction mixture was stirred at 110 °C under an Ar atmosphere (2 hours). Next, after cooling the reaction mixture to room temperature, the solvent was evaporated and the residue dissolved in anhydrous dichloromethane (2 mL), followed by the addition of trimethylsilyl chloride (0.444 mL, 3.50 mmol). The reaction mixture was stirred at room temperature under an Ar atmosphere for 15 hours. After removing the solvent, the product was purified by flash column chromatography (5 g silica gel, eluent: 2% ethyl acetate in hexane) and dissolved in anhydrous THF (1.5 mL). A solution of potassium hydroxide (1.01 mL, 1.01 mmol, 1 N in methanol) in anhydrous THF (2 mL) was added at room temperature and the reaction mixture was stirred for 2 hours. Next, water (10 mL) was added and the mixture was extracted twice with diethyl ether. The combined organic layers were washed with brine and dried over magnesium sulfate. The crude mixture was purified by column chromatography (6 g silica gel, eluent: 2% ethyl acetate in hexane) giving (+)-monachalure (**2**) as an oil (0.092 g, 0.343 mmol, 98% yield).

$[\alpha]_D^{20}$ = +0.87 ° (c = 1.00, CCl_4);

IR (film, cm^{-1}): 2957 s, 2925 s, 2855 s, 1467 s, 1379 w, 1261 w;

^1H NMR (400 MHz, $CDCl_3$): δ = 2.92-2.88 (2H, m), 1.57-1.17 (28H, m), 0.88 (3H, t, *J* = 6.7 Hz), 0.87 (3H, t, *J* = 6.8 Hz).

^{13}C NMR (101 MHz, $CDCl_3$): δ = 57.42, 32.05, 31.92, 29.74, 29.71, 29.48, 29.38, 27.96, 26.74, 26.72, 22.83, 22.72, 14.27, 14.22.

HRMS (+ESI): *m/z* calcd for $C_{18}H_{36}NaO$ $[M+Na]^+$: 291.2658; found: 291.2679.

8. Synthesis of (−)-disparlure (3) and (−)-monachalure (4)

8.1. Synthesis of (−)-*tert*-butyl-[[(2*R*,3*S*,5*R*,6*R*)-5,6-dimethoxy-5,6-dimethyl-3-(5-methylhexyl)-1,4-dioxan-2-yl]methoxy]-dimethylsilane (24)

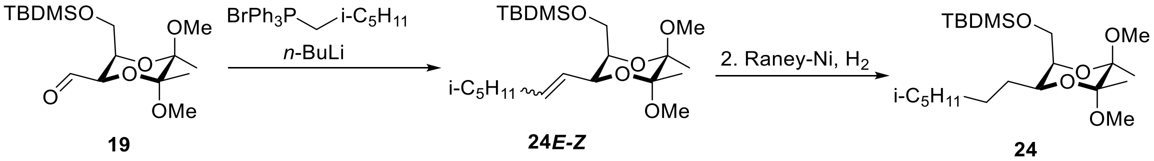

19 24*E-Z* 24

4-Methylpentyltriphenylphosphonium bromide (3.36 g, 7.87 mmol) was dried in a Schlenk flask at 60 °C under reduced pressure (10 mbar) for 1 hour. Next, anhydrous THF (30 mL) was added, followed by cooling to −25 °C and the addition of *n*-butyllithium (2.5 M in hexane, 3.15 mL, 7.87 mmol). After 30 min, aldehyde **19** (1.37 g, 3.94 mmol) in anhydrous THF (6 mL) was added at −78 °C. The cooling was turned off and the mixture was stirred until the temperature reached 0 °C (about 1 hour), and TLC analysis (eluent: 20% ethyl acetate in hexane) showed complete conversion of the substrate. Next, diethyl ether (20 mL) and water (20 mL) were added. The aqueous layer was extracted twice with 15 mL of diethyl ether and the combined organic layers were washed with brine, water and dried over magnesium sulfate. The crude reaction mixture, after removing the organic solvents, was purified by column chromatography (20 g silica gel, eluent: 2.5% ethyl acetate in hexane) giving olefin ***24E-Z*** as an oil (1.08 g, 2.58 mmol, 65% yield) as a mixture of geometric isomers in a ratio 1:0.2. Small amounts of geometric isomers were separated.

$[\alpha]_D^{20}$ = -86.2° (c = 1.00, $CHCl_3$);

IR (Film, cm^{-1}): 2991 m, 2955 s, 2930 s, 2857 s, 1471 m, 1373 m, 1256 m, 1150 s, 1138 s; HRMS (+ESI): *m/z* calcd for $C_{22}H_{44}O_5Si$ $[M+Na]^+$: 439.2850; found: 439.2850.

Isomer *E*:

^{1}H NMR (400 MHz, $CDCl_3$): δ = 5.52 (1H , ddd, *J* = 13.7 Hz, 9.1 Hz, 3.6 Hz), 5.40 (1H, ddd, *J* = 10.6 Hz, 2.6 Hz, 1.3 Hz), 4.98-4.91 (1H, m), 4.04 (1H, dd, *J* = 10.5 Hz, 6.7 Hz), 3.85 (1H, dd, *J* = 10.5 Hz, 5.8 Hz), 3.66-3.57 (1H , m), 3.31 (3H, s), 3.26 (3H, s), 2.19-1.97 (2H , m), 1.60-1.48 (1H , m), 1.29 (3H, s), 1.29 (3H , s), 1.28-1.19 (3H, m), 0.86 (12H, s), 0.02 (6H, d, *J* = 2.0 Hz).

^{13}C NMR (101 MHz, $CDCl_3$): δ = 133.68, 125.68, 99.80, 98.03, 75.44, 65.04, 62.26, 49.52, 48.13, 39.04, 27.76, 25.98, 25.86, 22.65, 18.65, 18.28, 18.23, -5.20.

Isomer *Z*:

^{1}H NMR (400 MHz, $CDCl_3$): δ = 5.62 (1H, ddd, *J* = 10.8 Hz, 9.3 Hz, 4.1 Hz), 5.25 (1H, ddt, *J* = 10.8 Hz, 9.3 Hz, 1.6 Hz), 4.81 (1H, t, *J* = 10.0 Hz), 3.75 (1H, dt, *J* = 10.3 Hz, 3.0 Hz), 3.68 (1H, dd, *J* = 11.2 Hz, 2.7 Hz), 3.54 (1H, dd, *J* = 11.2 Hz, 3.3 Hz), 3.39 (3H, s), 3.33 (3H, s), 2.29-2.17 (1H, m), 2.10-1.97 (1H, m), 1.63-1.50 (2H, m), 1.40 (6H, s), 1.29-1.19 (3H, m), 0.89 (13H, s), 0.05 (6H, d, *J* = 4.4 Hz).

^{13}C NMR (101 MHz, $CDCl_3$); δ = 136.43, 126.47, 100.42, 100.24, 75.42, 68.08, 63.39, 48.66, 48.59, 38.87, 27.82, 26.17, 26.12, 25.98, 22.76, 22.71, 22.63, 22.52, -5.11, -5.29.

Raney nickel (5.70 g, 50% in water) was washed 10 times with anhydrous methanol (10 mL), suspended in 10 mL of methanol and hydrogen was bubbled through the suspension. Next, olefin **24*E-Z*** (0.984 g, 2.36 mmol) in methanol (4 mL) was added to the mixture and a balloon filled with hydrogen was attached to the flask through a rubber septum. The reaction was vigorously stirred until TLC analysis (eluent: 5% ethyl acetate in hexane) showed complete conversion of the substrate (4 hours). Next, the flask was filled with argon and the mixture vigorously stirred for 5 min. The reaction mixture was filtered through a thin layer of silica gel, which was washed with diethyl ether. The crude reaction mixture, after removing the organic solvents, was purified by column chromatography (10 g silica gel, eluent: 5% ethyl acetate in hexane) giving (−)-*tert*-butyl-[[(2*R*,3*S*,5*R*,6*R*)-5,6-dimethoxy-5,6-dimethyl-3-(5-methylhexyl)-1,4-dioxan-2-yl]methoxy]-dimethylsilane (**24**) as a solid (0.965 g, 2.31 mmol, 98% yield)

m.p. = 46.7 °C

$[\alpha]_D^{20}$ = -94.5 ° (c = 1.00, CCl_4);

IR (KBr, cm^{-1}): 2990 s, 2951 s, 2928 s, 2857 s, 1468 s, 1374 s, 1260 m, 1136 s, 1150 s, 1078 s;

^{1}H NMR (400 MHz, $CDCl_3$): δ = 4.18-4.11 (1H, m), 4.04 (1H, dt, *J* = 7.9 Hz, 3.8 Hz), 3.71-3.64 (1H, m), 3.58 (1H, dt, *J* = 7.8 Hz, 3.8 Hz), 3.25 (3H, s), 3.23 (3H, s), 1.55-1.41 (4H, m), 1.32-1.22 (9H, m), 1.19-1.10 (2H, m), 0.88-0.86 (9H, m), 0.86 (3H, s), 0.84 (3H, br s), 0.04 (6H, br s).

^{13}C NMR (101 MHz, $CDCl_3$): δ = 99.58, 98.13, 75.41, 68.71, 62.40, 49.53, 47.91, 39.10, 30.92, 28.13, 27.62, 26.56, 26.00, 22.83, 22.74, 18.43, 18.27, 18.25, -5.25, -5.30.

HRMS (+ESI): *m/z* calcd for $C_{22}H_{46}NaO_5$ $[M+Na]^+$: 441,3007; found: 441,2954.

8.2. Synthesis of (−)-*tert*-butyl-[[(2*R*,3*S*,5*R*,6*R*)-3-hexyl-5,6-dimethoxy-5,6-dimethyl-1,4-dioxan-2-yl]methoxy]-dimethylsilane (25)

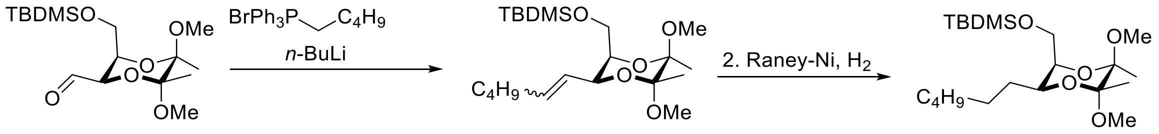

19 **25*E-Z*** **25**

n-Pentyltriphenylphosphonium bromide (3.71 g, 8.98 mmol) was dried in a Schlenk flask at 60 °C under reduced pressure (10 mbar) for 1 hour. Next, anhydrous THF (60 mL) was added, followed by cooling to −25 °C and the addition of *n*-butyllithium (2.5 M in hexane, 3.59 mL, 8.98 mmol). After 30 min, aldehyde **19** (1.56 g, 4.49 mmol) in anhydrous THF (8 mL) was added at −78 °C. The cooling was turned off and the mixture was stirred until the temperature reached 0 °C (about 1 hour), and TLC analysis (eluent: 20% ethyl acetate in hexane) showed complete conversion of the substrate. Next, diethyl ether (20 mL) and water (20 mL) were added. The aqueous layer was extracted twice with 15 mL of diethyl ether and the combined organic layers were washed with brine, water and dried over magnesium sulfate. The crude reaction mixture, after removing the organic solvents, was purified by column chromatography (50 g silica gel, eluent: 2.5% ethyl acetate in hexane) giving olefin **25*E-Z*** as an oil (1.71 g, 2.58 mmol, 98% yield) as a mixture of geometric isomers in a ratio 1:0.22.

$[\alpha]_D^{20}$ = -26.5 ° (c = 1.00, $CHCl_3$);

IR (Film, cm^{-1}): 2956 m, 2929 m, 2858 m, 1463 m, 1371 m, 1144 m, 1116 m, 775 s;

^1H NMR (400 MHz, $CDCl_3$): δ = 5.63 (1H from isomer *E* or *Z*, ddd, *J* = 10.9 Hz, 9.3 Hz, 4.3 Hz), 5.58-5.49 (1H from isomer *E* or *Z*, m), 5.47-5.38 (1H from isomer *E* or *Z*, m), 5.29-5.21 (1H from isomer *E* or *Z*, m), 4.95 (1H from isomer *E* or *Z*, dd, *J* = 8.9 Hz, 4.2 Hz), 4.87-4.77 (1H from isomer *E* or *Z*, m), 4.79-3.72 (1H from isomer *E* or *Z*, m), 3.62 (2H from isomer *E* or *Z*, ddd, *J* = 6.7 Hz, 5.9 Hz, 4.3 Hz), 3.96 (2H from isomer *E* or *Z*, ddd, *J* = 16.1 Hz, 10.5 Hz, 6.2 Hz), 3.62 (1H from isomer *E* or *Z*, ddd, *J* = 6.7 Hz, 5.9 Hz, 4.3 Hz), 3.39 (3H from isomer *E* or *Z*, s), 3.34 (3H, from isomer *E* or *Z*, s), 3.31 (3H from isomer *E* or *Z*, s), 3.27 (3H from isomer *E* or *Z*, s), 2.26-2.02 (4H from isomer *E* and *Z*, m), 1.41 (6H from isomer *E* or *Z*, br s) 1.38-1.24 (8H, from isomer *E* and *Z*), 1.30 (3H from isomer *E* or *Z*, s), 0.91-0.89 (12H from isomer *E* or *Z*, m), 0.89-0.85 (12H from isomer *E* or *Z*, m), 0.06 (6H from isomer *E* or *Z*, d, *J* = 6.0 Hz), 0.03 (6H from isomer *E* or *Z*, d, *J* = 2.1 Hz).

^{13}C NMR (101 MHz, $CDCl_3$): δ = 136.25, 126.60, 100.42, 100.23, 75.38, 68.07, 63.34, 48.67, 48.58, 31.88, 27.97, 26.11, 22.56, 18.55, 17.80, 17.69, 14.12, -5.14, -5.29.
HRMS (+ESI): *m/z* calcd for $C_{21}H_{42}NaO_5Si$ $[M+Na]^+$: 425.2634; found: 425.2690.

Raney nickel (1.44 g, 50% in water) was washed 10 times with anhydrous methanol (10 mL), suspended in 10 mL of methanol and hydrogen was bubbled through the suspension. Next, olefin **25*E-Z*** (0.230 g, 0.572 mmol) in methanol (4 mL) was added to the mixture and a balloon filled with hydrogen was attached to the flask through a rubber septum. The reaction mixture was vigorously stirred until TLC analysis (eluent: 5% ethyl acetate in hexane) showed complete conversion of the substrate (2 hours). Next, the flask was filled with argon and the mixture vigorously stirred for 5 min. The reaction mixture was filtered through a thin layer of silica gel, which was washed with diethyl ether. The crude reaction mixture, after removing the organic solvents, was purified by column chromatography (20 g silica gel, eluent: 5% ethyl acetate in hexane) giving (−)-*tert*-butyl-[[(2*R*,3*S*,5*R*,6*R*)-3-hexyl-5,6-dimethoxy-5,6-dimethyl-1,4-dioxan-2-yl]methoxy]-dimethylsilane (**25**) as an oil (0.229 g, 0.566 mmol, 99% yield).
$[\alpha]_D^{20}$ = -90.9 ° (c = 1.00, $CHCl_3$);
IR (Film, cm^{-1}): 2954 s, 2930 s, 2857 s, 1463 m, 1373 m, 1139 s, 1116 s, 837 s;
^1H NMR (400 MHz, $CDCl_3$): δ = 4.00 (1H, dd, *J* = 12.9 Hz, 5.2 Hz), 3.74-3.61 (4H, m), 3.34 (3H, s), 3.32 (3H, s), 3.25 (1H, m), 1.38 (3H, s), 1.37 (3H, s), 1.31-1.24 (8H, m) 0.92-0.85 (12H, m), 0.05 (6H, d, *J* = 2.5 Hz).
^{13}C NMR (101 MHz, $CDCl_3$): δ = 99.59, 98.13, 75.41, 68.71, 62.40, 49.53, 47.92, 31.97, 30.92, 29.57, 26.29, 25.99, 22.82, 18.44, 18.28, 18.25, 14.26, -5.25, -5.30.
HRMS (+ESI): *m/z* calcd for $C_{21}H_{44}NaO_5Si$ $[M+Na]^+$: 427.2850; found: 427.2797.

8.3. Synthesis of (−)-(2*S*,3*S*,5*R*,6*R*)-5,6-dimethoxy-5,6-dimethyl-3-(5-methylhexyl)-1,4-dioxane-2-carbaldehyde (26)

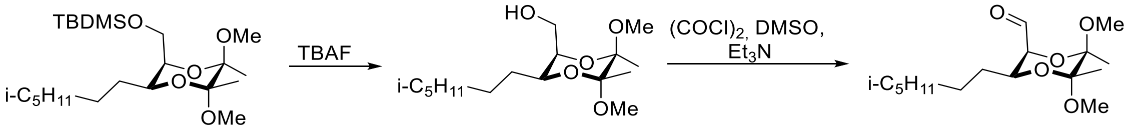

24 **24a** **26**

Tetra-*n*-butylammonium fluoride (1 M in THF, 3.22 mL, 3.22 mmol) was added to (−)-*tert*-butyl-[[(2*R*,3*S*,5*R*,6*R*)-5,6-dimethoxy-5,6-dimethyl-3-(5-methylhexyl)-1,4-dioxan-2-yl]methoxy]-dimethylsilane (**24**, 0.906 g, 2.16 mmol) in anhydrous THF (13 mL) at room temperature. The reaction mixture was stirred until TLC analysis (eluent: 5% ethyl acetate in hexane) showed complete conversion of the substrate (5 hours). Next, saturated sodium hydrogencarbonate (10 mL) was added. The aqueous layer was extracted twice with 10 mL of ethyl acetate and the combined organic layers were washed with brine, water and dried over magnesium sulfate. The crude reaction mixture, after removing the organic solvents, was purified by column chromatography (10 g silica gel, eluent: 20% ethyl acetate in hexane) giving alcohol **24a** as an oil (0.635 g, 2.09 mmol, 96% yield).

$[\alpha]_D^{20}$ = -112.0 ° (c = 1.00, CCl_4);

IR (Film, cm^{-1}): 3479 b s, 2992 m, 2952 s, 2869 m, 2834 m, 1463 s, 1374 s, 1136 s, 1118 s, 1037 s;

^1H NMR (400 MHz, $CDCl_3$): δ = 4.11-4.02 (1H, m), 3.80 (2H, m), 3.57 (1H, t, *J* = 3.9 Hz), 3.38 (3H, s), 3.23 (3H, s), 1.78 (1H, ddd, *J* = 13.2 Hz, 9.8 Hz, 5.1 Hz, 1.59-1.44 (3H, m), 1.37-1.28 (3H, m), 1.34 (3H, s), 1.29 (3H, s), 1.28-1.11 (3H, m), 0.86 (3H, s), 0.84 (3H, s).

^{13}C NMR (101 MHz, $CDCl_3$): δ = 99.41, 97.93, 74.31, 68.39, 63.39, 49.30, 47.96, 39.10, 30.42, 28.09, 27.50, 26.50, 22.77, 22.74, 18.43, 18.19.

HRMS (+ESI): *m/z* calcd for $C_{16}H_{32}NaO_5$ $[M+Na]^+$: 327.2142; found: 327.2094.

DMSO (0.335 mL, 5.01 mmol) was added dropwise to a mixture of oxalyl chloride (0.265 mL, 3.13 mmol) in anhydrous dichloromethane (10 mL) at −60 °C. After stirring for 5 min, alcohol **24a** (0.626 g, 2.09 mmol) in anhydrous dichloromethane (2 mL) was added. The reaction was carried out for 15 min at −60 °C followed by the addition of triethylamine (0.872 mL, 6.25 mmol). After the reaction mixture was warmed to room temperature water (10 mL) was added. The aqueous layer was

extracted twice with 10 mL of dichloromethane and the combined organic layers were washed with saturated sodium hydrogencarbonate and dried over magnesium sulfate. The crude reaction mixture, after removing the organic solvents, was purified by column chromatography (20 g silica gel, eluent: 5% ethyl acetate in hexane) giving (−)-(2*S*,3*S*,5*R*,6*R*)-5,6-dimethoxy-5,6-dimethyl-3-(5-methylhexyl)-1,4-dioxane-2-carbaldehyde (**26**) as an oil (0.331 g, 1.09 mmol, 52% yield).

$[\alpha]_D^{20}$ = -121.3 ° (c = 1.00, $CHCl_3$);

IR (Film, cm^{-1}): 2993 m, 2953 s, 2869 m, 1724 s, 1466 s, 1376 s, 1142 s, 1119 s, 1038 s;

^1H NMR (400 MHz, $CDCl_3$): δ = 9.96 (1H, dd, *J* = 2.9 Hz, 0.7 Hz), 4.13-4.06 (1H, m), 3.60-3.55 (1H, m), 3.27 (3H, s), 3.18 (3H, s), 1.70 (1H, m), 1.56-1.43 (3H, m), 1.35-1.22 (9H, m), 1.19-1.11 (2H, m), 0.86 (3H, s), 0.84 (3H, s).

^{13}C NMR (101 MHz, $CDCl_3$): δ = 200.89, 99.82, 98.97, 76.24, 67.47, 49.32, 48.16, 38.95, 29.85, 28.06, 27.34, 26.29, 22.76, 22.72, 18.14, 17.56.

HRMS (+ESI): *m/z* calcd for $C_{16}H_{30}NaO_6$ $[M+Na]^+$: 325.1985; found: 325.1207.

8.4. Synthesis of (-)-(2*S*,3*S*,5*R*,6*R*)-3-hexyl-5,6-dimethoxy-5,6-dimethyl-1,4-dioxane-2-carbaldehyde (27)

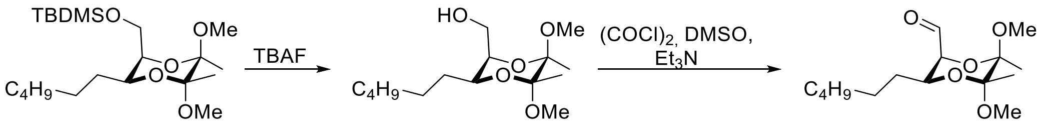

25 **25a** **27**

Tetra-*n*-butylammonium fluoride (1 M in THF, 2.46 mL, 2.46 mmol) was added to (−)-*tert*-butyl-[[(2*R*,3*S*,5*R*,6*R*)-3-hexyl-5,6-dimethoxy-5,6-dimethyl-1,4-dioxan-2-yl]methoxy]-dimethylsilane (**25**, 0.663 g, 1.64 mmol) in anhydrous THF (10 mL) at room temperature. The reaction mixture was stirred until TLC analysis (eluent: 5% ethyl acetate in hexane) showed complete conversion of the substrate (5 hours). Next, saturated sodium hydrogencarbonate (10 mL) was added. The aqueous layer was extracted twice with 10 mL of ethyl acetate and the combined organic layers were washed with brine, water and dried over magnesium sulfate. The crude reaction mixture, after removing the organic solvents, was purified by column chromatography (10 g silica gel, eluent: 20% ethyl acetate in hexane) giving alcohol **25a** as an oil (0.472 g, 1.62 mmol, 99% yield).

$[\alpha]_D^{20}$ = -111.2 ° (c = 1.00, $CHCl_3$);

IR (Film, cm^{-1}): 3478 sb, 2953 s, 2930 s, 1459 m, 1375 m, 1193 s, 1118 s, 1038 s;
^{1}H NMR (400 MHz, $CDCl_3$): δ = 4.11-4.03 (1H, m), 3.82 (2H, t, *J* = 3.4 Hz), 3.57 (1H, m), 3.39 (3H, s), 3.24 (3H, s), 1.78 (1H, ddd, *J* = 13.6 Hz, 9.6 Hz, 5.2 Hz), 1.52 (2H, m), 1.40-1.18 (14H, m), 0.87 (3H, t, *J* = 6.5 Hz).
^{13}C NMR (101 MHz, $CDCl_3$): δ = 106.50, 72.10, 64.17, 48.38, 31.94, 31.83, 30.58, 29.29, 25.27, 22.73, 18.58, 18.47, 14.21.
HRMS (+ESI): *m/z* calcd for $C_{15}H_{30}NaO_5$ $[M+Na]^+$: 313.1985; found: 313.1950.

DMSO (0.276 mL, 3.90 mmol) was added dropwise to a mixture of oxalyl chloride (0.206 mL, 2.44 mmol) in anhydrous dichloromethane (10 mL) at -60 °C. After stirring for 5 min, the alcohol **25a** (0.472 g, 1.62 mmol) in anhydrous dichloromethane (2 mL) was added. The reaction was carried out for 15 min at −60 °C followed by addition of triethylamine (0.678 mL, 4.87 mmol). After the reaction mixture was warmed to room temperature, water (10 mL) was added. The aqueous layer was extracted twice with 10 mL of dichloromethane and the combined organic layers were washed with saturated sodium hydrogencarbonate and dried over magnesium sulfate. The crude reaction product (−)-(2*S*,3*S*,5*R*,6*R*)-3-hexyl-5,6-dimethoxy-5,6-dimethyl-1,4-dioxane-2-carbaldehyde (**27**) was obtained as an oil (≈ 0.477 g) and used in the next step without purification.
$[\alpha]_D^{20}$ = -116.5 ° (c = 1.00, $CHCl_3$);
Lit. $[\alpha]_D^{28}$ = -122.0 ° (c = 1.08, $CHCl_3$);[2]
IR (Film, cm^{-1}): 2954 m, 2930 m, 2858 m, 1724 s, 1459 s, 1377 s, 1142 s, 1118 s;
^{1}H NMR (400 MHz, $CDCl_3$): δ = 9.60 (1H, d, *J* = 1.6 Hz), 4.08 (1H, dd, *J* = 9.7 Hz, 1.7 Hz), 4.00-3.92 (1H, m), 3.40 (3H, s), 3.33 (3H, s), 1.57-1.18 (10H, m), 1.43 (3H, s), 1.37 (3H, s), 0.87 (3H, t, *J* = 6.8 Hz).
HRMS (+ESI): *m/z* calcd for $C_{15}H_{28}NaO_5$ $[M+Na]^+$: 311.1829; found: 311.1822

8.5. Synthesis of (-)-(2*R*,3*R*,5*R*,6*S*)-5-decyl-2,3-dimethoxy-2,3-dimethyl-6-(5-methylhexyl)-1,4-dioxane (28)

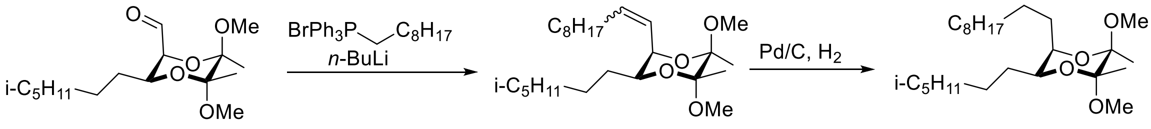

26 **28*E-Z*** **28**

n-Nonyltriphenylphosphonium bromide (0.890 g, 1.99 mmol) was dried in a Schlenk flask at 60 °C under reduced pressure (10 mbar) for 1 hour. Next, anhydrous THF (15 mL) was added, followed by cooling to −25 °C and the addition of *n*-butyllithium (2.5 M in hexane, 0.758 mL, 1.99 mmol). After 30 min, (−)-(2*S*,3*S*,5*R*,6*R*)-5,6-dimethoxy-5,6-dimethyl-3-(5-methylhexyl)-1,4-dioxane-2-carbaldehyde (**26**, 0.287 g 0.948 mmol) in anhydrous THF (2 mL) was added at −78 °C. The cooling was turned off and the mixture stirred until the temperature reached 0 °C (about 1 hour), and TLC analysis (eluent: 20% ethyl acetate in hexane) showed complete conversion of the substrate. Next, diethyl ether (20 mL) and water (20 mL) were added. The aqueous layer was extracted twice with 15 mL of diethyl ether and the combined organic layers were washed with brine, water and dried over magnesium sulfate. The crude reaction mixture, after removing the organic solvents, was purified by column chromatography (20 g silica gel, eluent: 2% ethyl acetate in hexane) giving olefin **28*E-Z*** as an oil (0.293 g, 0.709 mmol, 74% yield) as a mixture of geometric isomers in a ratio 1:0.2.

$[\alpha]_D^{20}$ = -50.3 ° (c = 1.00, $CHCl_3$);

IR (Film, cm^{-1}): 2990 m, 2954 s, 2927 s, 2855 m, 1653 w, 1466 m, 1373 m, 1138 s, 1116 s, 1040 s;

^1H NMR (400 MHz, $CDCl_3$): δ = 6.04-5.96 (1H from isomer *E* or *Z*, m), 5.96-5.88 (1H from A, m), 5.60 (2H from isomer *E* and *Z*, dt, *J* = 10.6 Hz, 7.5 Hz), 4.27 (1H from isomer *E* or *Z*, ddd, *J* = 10.3 Hz, 3.9 Hz, 0.7 Hz), 4.09-4.03 (1H from isomer *E* or *Z*, m), 4.02 (1H from isomer *E* or *Z*, dd, *J* = 8.8 Hz, 4.7 Hz), 3.83 (1H from isomer *E* or *Z*, dd, *J* = 10.3 HZ, 4.0 Hz), 3.25 (6H from isomer *E* and *Z*, s), 3.17 (3H from isomer *E* or *Z*, s), 3.13 (3H from isomer *E* or *Z*, s), 2.17-1.96 (8H from isomer *E* and *Z*, m), 1.53-1.10 (44H from isomer *E* and *Z*, m), 0.90-0.81 (24H from isomer *E* and *Z*, m).

^{13}C NMR (101 MHz, $CDCl_3$): δ = 133.75, 125.77, 99.07, 97.58, 68.39, 68.11, 50.48, 48.24, 47.36, 38.52, 31.46, 30.62, 29.24, 29.02, 28.89, 27.49, 27.00, 26.66, 25.31, 23.00, 22.25, 22.21, 18.07, 17.56, 13.69.

HRMS (+ESI): *m/z* calcd for $C_{25}H_{48}NaO_4$ $[M+Na]^+$: 435.3445; found: 435.3404.

The solution of olefin **28*E-Z*** (0.221 g, 0.536 mmol) in anhydrous THF (5 mL) was placed in a round-bottomed flask which was filled with argon and then Pd/C (10 mol%, 0.057 g, 10%) was added. The flask was closed with a rubber septum and a balloon filled with hydrogen was attached through a rubber septum. The mixture was vigorously stirred until TLC analysis (eluent: 5% ethyl acetate in hexane) showed complete conversion of the substrate. Next, the flask was filled with argon and the mixture vigorously stirred for 5 min. The reaction mixture was filtered through a thin layer of Celite, which was washed with ethyl acetate. The crude reaction mixture, after removing the organic solvents, was purified by column chromatography (7 g silica gel, eluent: 1% ethyl acetate in hexane) giving (−)-(2*R*,3*R*,5*R*,6*S*)-5-decyl-2,3-dimethoxy-2,3-dimethyl-6-(5-methylhexyl)-1,4-dioxane (**28**) as an oil (0.137 g, 0.331 mmol, 62% yield).
$[\alpha]_D^{20}$ = -30.0 ° (c = 1.00, $CHCl_3$);
IR (Film, cm^{-1}): 2954 s, 2923 s, 2853 s, 1468 s, 1373 s, 1137 s, 1113 s, 1041 s;
^1H NMR (400 MHz, $CDCl_3$): δ = 4.06 (1H, dt, *J* = 8.7 Hz, 4.2 Hz), 3.51-3.42 (1H, m), 3.28 (3H, s), 3.23 (3H, s), 1.97-1.85 (1H, m), 1.56-1.08 (32H, m), 0.92-0.80 (9H, m).
^{13}C NMR (101 MHz, $CDCl_3$): δ = 99.83, 98.80, 75.06, 69.04, 49.39, 47.89, 39.11, 32.06, 31.59, 29.97, 29.84, 29.81, 29.77, 29.48, 28.77, 28.09, 27.60, 27.55, 26.44, 22.83, 22.78, 22.73, 19.02, 18.34, 14.25.
HRMS (+ESI): *m/z* calcd for $C_{25}H_{50}NaO_4$ $[M+Na]^+$: 437.3601; found: 437.3554.

8.6. Synthesis of (−)-(2*R*,3*R*,5*R*,6*S*)-5-decyl-6-hexyl-2,3-dimethoxy-2,3-dimethyl-1,4-dioxane (29)

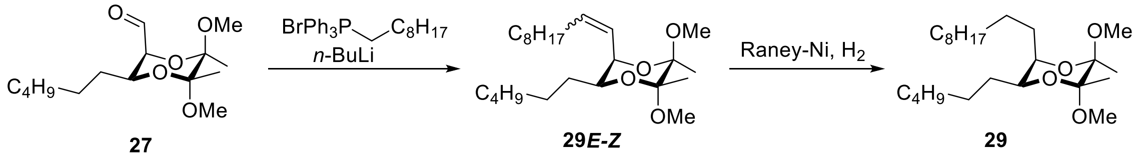

27 **29*E-Z*** **29**

n-Nonyltriphenylphosphonium bromide (0.137 g, 2.92 mmol) was dried in a Schlenk flask at 60 °C under reduced pressure (10 mbar) for 1 hour. Next, anhydrous THF (10 mL), was added, followed by cooling to −25 °C and the addition of *n*-butyllithium (2.5 M in hexane, 1.17 mL, 2.92 mmol). After 30 min, (−)-(2*S*,3*S*,5*R*,6*R*)-3-hexyl-5,6-dimethoxy-5,6-dimethyl-1,4-dioxane-2-carbaldehyde (**27**, 0.447 g, 1.62 mmol) in

anhydrous THF (4 mL) was added at −78 °C. The cooling was turned off and the mixture was stirred until the temperature reached 0 °C (about 1 hour), and TLC analysis (eluent: 20% ethyl acetate in hexane) showed complete conversion of the substrate. Next, diethyl ether (20 mL) and water (20 mL) were added. The aqueous layer was extracted twice with 15 mL of diethyl ether and the combined organic layers were washed with brine, water and dried over magnesium sulfate. The crude reaction mixture, after removing the organic solvents, was purified by column chromatography (20 g silica gel, eluent: 2% ethyl acetate in hexane) giving olefin ***29E-Z*** as an oil (0.316 g, 0.794 mmol, 49% yield) as a mixture of geometric isomers in a ratio 1:0.2.

$[\alpha]_D^{20}$ = -97.5 ° (c = 1.00, $CHCl_3$);

IR (Film, cm^{-1}): 2956 m, 2929 m, 2858 m, 1463 m, 1371 m, 1144 m, 1116 m, 775 s;

^{1}H NMR (400 MHz, $CDCl_3$): δ = 5.99 (1H from isomer *E* or *Z*, m, *J* = 15.3 Hz, 10.2 Hz, 1.3 Hz), 5.96-5.88 (1H from isomer *E* or *Z*, m), 5.65-5.53 (1H from isomer *E* or *Z*, m), 5.23 (1H from isomer *E* or *Z*, ddt, *J* = 10.8 Hz, 9.2 Hz, 1.6 Hz), 4.27 (1H from isomer *E* or *Z*, ddd, *J* = 10.3 Hz, 3.9 Hz, 0.8 Hz), 4.46-4.39 (1H from isomer *E* or *Z*, m), 4.09-4.02 (2H from isomer *E* and *Z*, m), 3.83 (1H from isomer *E* or *Z*, dd, *J* = 10.2 Hz, 3.9 Hz), 3.67-3.62 (1H from isomer *E* or *Z*, m) 3.35 (6H from isomer *E* and *Z*, s), 3.33 (6H from isomer *E* and *Z*, s), 2.16-1.99 (2H from isomer *E* or *Z*, m), 1.51-1.18 (56H from isomer *E* and *Z*, m), 0.90-0.82 (12H from isomer *E* and *Z*, m).

^{13}C NMR (101 MHz, $CDCl_3$); δ = 106.48, 99.55, 77.73, 77.43, 72.08, 70.11, 64.15, 49.18, 48.36, 31.81, 31.73, 30.56, 29.28, 29.20, 29.03, 25.25, 24.99, 22.71, 22.68, 18.61, 18.57, 18.46, 18.08, 14.19.

HRMS (+ESI): *m/z* calcd for $C_{24}H_{46}O_4$ $[M+Na]^+$: 421.3288; found: 421.3241.

Raney nickel (0.64 g, 50% in water) was washed 10 times with anhydrous methanol (10 mL), suspended in 10 mL of methanol and hydrogen was bubbled through the suspension. Next, olefin ***29E-Z*** (0.095 g, 0.256 mmol) in methanol (2 mL) was added to the mixture and a balloon filled with hydrogen was attached to the flask through a rubber septum. The reaction mixture was vigorously stirred until TLC analysis (eluent: 5% ethyl acetate in hexane) showed complete conversion of the substrate (2 hours). Next, the flask was filled with argon and the mixture vigorously stirred for 5 min. The reaction mixture was filtered through a thin layer of silica gel, which was washed with diethyl ether. The crude reaction mixture, after removing the organic

solvents, was purified by column chromatography (10 g silica gel, eluent: 5% ethyl acetate in hexane) giving (−)-(2*R*,3*R*,5*R*,6*S*)-5-decyl-6-hexyl-2,3-dimethoxy-2,3-dimethyl-1,4-dioxane (**29**) as an oil (0.0835 g, 0.224 mmol, 88% yield).

$[\alpha]_D^{20}$ = -2.4 ° (c = 1.00, $CHCl_3$);

IR (Film, cm^{-1}): 2953 s, 2922 s, 2854 s, 1468 s, 1374 s, 1137 s, 1113 s, 1040 s;

^1H NMR (400 MHz, $CDCl_3$): δ = 4.06 (1H, dt, *J* = 8.7 Hz, 4.2 Hz), 3.50-3.43 (1H, m), 3.29 (3H, s), 3.24 (3H, s), 1.98-1.85 (1H, m), 1.66-1.16 (33H, m), 0.88 (6H, t, *J* = 6.5 Hz).

^{13}C NMR (101 MHz, $CDCl_3$): δ = 99.84, 98.81, 75.07, 69.06, 49.39, 47.89, 32.06, 31.94, 31.55, 29.82, 29.77, 29.66, 29.49, 28.77, 26.16, 26.12, 22.83, 22.78, 19.02, 18.35, 14.25, 14.22.

HRMS (+ESI): *m/z* calcd for $C_{24}H_{48}NaO_4$ $[M+Na]^+$: 423.3445; found: 423.3357.

8.7. Synthesis of (-)-(7*S*,8*R*)-2-methylnonadecane-7,8-diol (7)

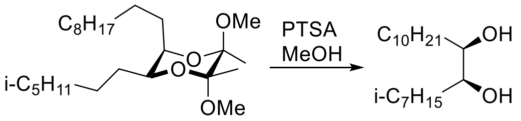

28 **7**

p-Toluenesulfonic acid (0.075 g, 0.393 mmol) was added to (−)-(2*R*,3*R*,5*R*,6*S*)-5-decyl-2,3-dimethoxy-2,3-dimethyl-6-(5-methylhexyl)-1,4-dioxane (**28**, 0.109 g, 0.262 mmol) in anhydrous methanol (6 mL). The reaction mixture was stirred at 60 °C under an Ar atmosphere until TLC analysis (eluent: 5% ethyl acetate in hexane) showed complete conversion of the substrate. Next, after cooling the reaction mixture to room temperature, anhydrous triethylamine (0.0551 mL, 0.394 mmol) was added dropwise and the solvent was evaporated. The crude mixture was purified by column chromatography (20 g silica gel, eluent: 20% ethyl acetate in hexane) giving (−)-(7*S*,8*R*)-2-methylnonadecane-7,8-diol (**7**) as a solid (0.053 g, 0.177 mmol, 68% yield).

m.p. = 89.4 °C;

$[\alpha]_D^{20}$ = -1.37 ° (c = 1.00, $CHCl_3$);

IR (KBr, cm^{-1}): 3327 b s, 2956 s, 2916 b s, 1470 s, 1069 s, 1031 m;

^1H NMR (400 MHz, $CDCl_3$): δ = 3.65-3.55 (2H, m), 1.93 (2H, br s), 1.56-1.11 (27H, m), 0.93-0.82 (9H, m).

^{13}C NMR (101 MHz, $CDCl_3$): δ = 74.86, 39.08, 32.05, 31.38, 29.83, 29.76, 29.48, 28.08, 27.57, 26.42, 26.17, 22.83, 22.78, 22.75, 14.25.
HRMS (+ESI): *m/z* calcd for $C_{19}H_{40}NaO_2$ $[M+Na]^+$: 323.2921; found: 323.2893.

8.8. Synthesis of (-)-(7*S*,8*R*)-nonadecane-7,8-diol (8)

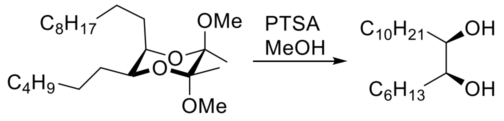

29 **8**

p-Toluenesulfonic acid (0.31 g, 0.87 mmol) was added to (−)-(2*R*,3*R*,5*R*,6*S*)-5-decyl-6-hexyl-2,3-dimethoxy-2,3-dimethyl-1,4-dioxane (**29**, 0.33 g, 0.82 mmol) in anhydrous methanol (12 mL). The reaction mixture was stirred at 60 °C under an Ar atmosphere until TLC analysis (eluent: 5% ethyl acetate in hexane) showed complete conversion of the substrate. Next, after cooling the reaction mixture to room temperature anhydrous triethylamine (0.160 mL, 1.15 mmol) was added dropwise and the solvent was evaporated. The crude mixture was purified by column chromatography (10 g silica gel, eluent: 40% ethyl acetate in hexane) giving (−)-(7*S*,8*R*)-nonadecane-7,8-diol (**8**) as a solid (0.22 g, 0.76 mmol, 93% yield).
m.p. = 115.4 °C;
$[\alpha]_D^{20}$ = -2.4 ° (c = 1.00, $CHCl_3$);
IR (KBr, cm^{-1}): 3290 bs, 2954 s, 2916 s, 2849 s, 1469 s, 1377 m, 1317 m, 1069 s;
^1H NMR (400 MHz, $CDCl_3$): δ = 3.60 (2H, br s), 1.84 (2H,s), 1.56-1.20 (28H, m), 0.88 (6H, m,).
^{13}C NMR (101 MHz, $CDCl_3$): δ = 74.85, 32.05, 31.94, 31.36, 29.83, 29.76, 29.49, 26.16, 26.13, 22.83, 22.76, 14.26, 14.22.
HRMS (+ESI): *m/z* calcd for $C_{18}H_{38}NaO_2$ $[M+Na]^+$: 309.2764; found: 309.2744.

8.9. Synthesis of (−)-disparlure (3)

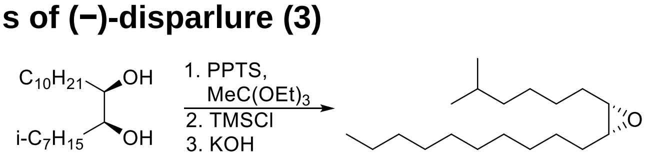

7 **3**

Triethyl orthoacetate (0.134 mL, 0.732 mmol) and pyridinium *p*-toluenesulfonate (2 mg) were added to (−)-(7*S*,8*R*)-2-methylnonadecane-7,8-diol (**7**, 0.044 g, 0.15 mmol) in anhydrous toluene (1.5 mL). The reaction mixture was stirred at

110 °C under an Ar atmosphere (2 hours). Next, after cooling the reaction mixture to room temperature, the solvent was evaporated and the residue dissolved in anhydrous dichloromethane (2 mL), followed by the addition of trimethylsilyl chloride (0.185 mL, 1.46 mmol). The reaction mixture was stirred at room temperature under an Ar atmosphere for 15 hours. After removing the solvent, the product was purified by flash column chromatography (5 g silica gel, eluent: 2% ethyl acetate in hexane) and dissolved in anhydrous THF (2 mL). A solution of potassium hydroxide (1 N in methanol, 0.420 mL, 0.420 mmol) in anhydrous THF (2 mL) was added at room temperature and the reaction mixture was stirred for 2 hours. Next, water (10 mL) was added and the mixture extracted twice with diethyl ether. The combined organic layers were washed with brine and dried over magnesium sulfate. The crude mixture was purified by column chromatography (6 g silica gel, eluent: 2% ethyl acetate in hexane) giving (−)-disparlure (**3**) as an oil (0.0335 g, 0.12 mmol, 81% yield).
$[\alpha]_D^{20}$ = -1.0 ° (c = 1.00, CCl_4);
Lit. $[\alpha]_D^{22}$ = -0.7 ° (c = 0.50, CCl_4) [3]; Lit. $[\alpha]_D$ = -0.9 ° (c = 0.21, CCl_4)[5];
Lit. $[\alpha]_D^{25}$ = -1.6 ° (c = 1.20, CCl_4) [6]; Lit. $[\alpha]_D$ = -1.0 ° (c = 1.50, CCl_4) [7];
FTIR (Film, cm^{-1}): 2955 m, 2925 m, 2856 m, 1467 s, 1385 s, 1366 s;
^{1}H NMR (400 MHz, $CDCL_3$) δ = 2.91-2.88 (2H, m), 1.56-1.15 (27H, m), 0.89-0.84 (9H, m).
^{13}C NMR (101 MHz, $CDCl_3$) δ = 57.42, 39.04, 32.05, 29.75, 29.71, 29.48, 28.04, 28.00, 27.96, 27.48, 27.00, 26.74, 22.83, 22.76, 14.27.
HRMS (+ESI): *m/z* calcd for $C_{19}H_{38}NaO$ $[M+Na]^+$: 305.2815; found: 305.2826.

8.10. Synthesis of (-)-monachalure (4)

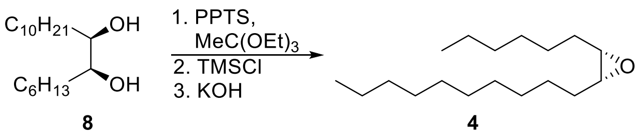

Triethyl orthoacetate (0.333 mL, 0.182 mmol)l) and pyridinium *p*-toluenesulfonate (2 mg) were added to (−)-(7*S*,8*R*)-nonadecane-7,8-diol (**8**, 0.104 g, 0.363 mmol) in anhydrous toluene (2 mL). The reaction mixture was stirred at 110 °C under an Ar atmosphere (2 hours). Next, after cooling the reaction mixture to room temperature, the solvent was evaporated and the residue was dissolved in anhydrous dichloromethane (2 mL), followed by the addition of trimethylsilyl chloride (0.461 mL, 3.63 mmol). The reaction mixture was stirred at room temperature under an Ar

atmosphere for 15 hours. After removing the solvent, the product was purified by flash column chromatography (5 g silica gel, eluent: 2% ethyl acetate in hexane) and dissolved in anhydrous THF (2 mL). A solution of potassium hydroxide (1 N in methanol, 1.05 mL, 1.05 mmol) in anhydrous THF (1.5 mL) was added at room temperature and the reaction mixture was stirred for 2 hours. Next, water (10 mL) was added and the mixture extracted twice with diethyl ether. The combined organic layers were washed with brine and dried over magnesium sulfate. The crude reaction mixture was purified by column chromatography (10 g silica gel, eluent: 5% ethyl acetate in hexane) giving (−)-monachalure (**4**) as an oil (0.082 g, 0.307 mmol, 85% yield).

$[\alpha]_D^{20}$ = -0.93 ° (c = 1.00, CCl_4);

IR (Film, cm^{-1}): 2957 s, 2925 s, 2855 s, 1467 s, 1378 m;

^1H NMR (400 MHz, $CDCl_3$): δ = 2.96-2.85 (2H, m), 1.56-1.19 (28H, m), 0.88 (6H, m).

^{13}C NMR (101 MHz, $CDCl_3$): δ = 57.42, 32.05, 31.93, 29.74, 29.71, 29.48, 29.38, 27.97, 26.74, 26.72, 22.83, 22.72, 14.27, 14.22.

HRMS (+ESI): *m/z* calcd for $C_{18}H_{36}NaO$ $[M+Na]^+$: 291.2658; found: 291.2682.

9. Determination of the enantiomeric purity of (+)- and (−)-disparlure 1 and 3 via GC–MS of *cis*-*N*-(α-methylbenzyl)aziridine derivatives

The derivatization procedures of (+)- and (−)-disparlure **1** and **3** were used according to known literature methods [9,10].

9.1. Derivatization of (+)-disparlure (1)

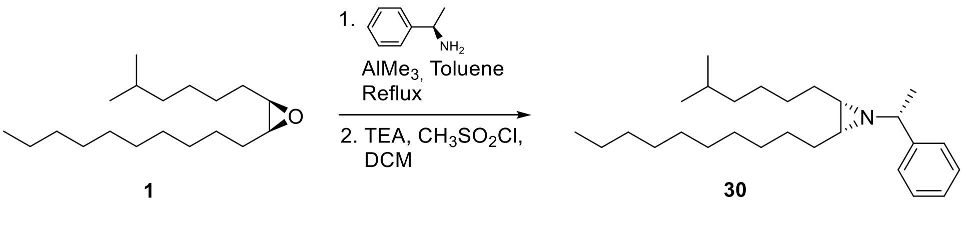

To a stirred solution of (+)-α-methylbenzylamine (10.7 mg, 11.2 µL, 0.0883 mmol) in toluene (0.5 mL), trimethylaluminum in hexane (0.05 mL, 2 M, 0.10 mmol) was added dropwise at 0 °C under an argon atmosphere. After that, a solution of (+)-disparlure (**1**, 25.0 mg, 0.0883 mmol) in toluene (1 mL) was added and the reaction mixture heated at reflux for 4 hours. After cooling, the reaction mixture was diluted with water (2 mL) and extracted with three portions of hexanes (2 mL). The combined extracts were rinsed well with water (2 mL) and dried over sodium sulfate. The crude mixture

was purified by column chromatography (5 g silica gel, eluent: 20% ethyl acetate in hexane) giving 26 mg of a mixture of the amino alcohols.

To a solution of the amino alcohols (26 mg) in dichloromethane (0.5 mL), trimethylamine (23 µL) and methanesulfonyl chloride (5.2 µL) were added. After stirring for 16 hours, the reaction mixture was diluted with aqueous sodium carbonate and extracted three times with pentanes (2 mL). The combined extracts were rinsed with water (2 mL) and dried over sodium sulfate. The crude mixture was purified by column chromatography (5 g silica gel, eluent: 5% ethyl acetate in hexane) giving *cis*-*N*-(α-methylbenzyl)aziridine (**30**, 23 mg). Mass spectra of **30**, m/z (relative intensity, %): 385 (M^+, 1), 384 (3), 370 (1), 280 (100), 244 (5), 105 (18), 82 (2), 69 (5), 55 (5).

9.2. Derivatization of (−)-disparlure (3)

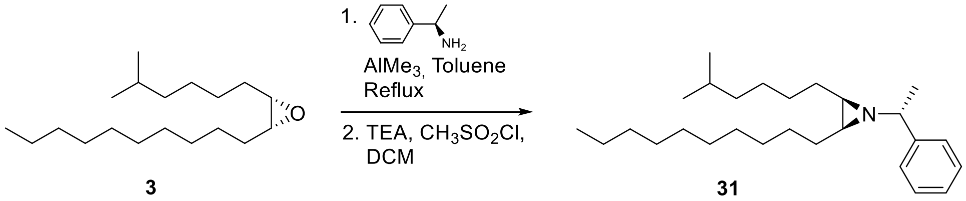

To a stirred solution of (+)-α-methylbenzylamine (2.14 mg, 2.24 µl, 0.0177 mmol) in toluene (0.2 mL), trimethylaluminum in hexane (0.01 mL, 2M, 0.02 mmol) was added dropwise at 0 °C under an argon atmosphere. After that, a solution of (−)-disparlure (**3**, 5 mg, 0.0177 mmol) in toluene (0.5 mL) was added and the reaction mixture heated at reflux for 4 h. After cooling, the reaction mixture was diluted with water (2 mL) and extracted with three portions of hexanes (2 mL). The combined extracts were rinsed well with water (2 mL) and dried over sodium sulfate. The crude mixture was purified by column chromatography (5 g silica gel, eluent: 20% ethyl acetate in hexane) giving 4.0 mg of a mixture of amino alcohols.

A solution of the amino alcohols (4.0 mg) in dichloromethane (0.5 mL), trimethylamine (3.5 µL) and methanesulfonyl chloride (0.8 µL) were added. After stirring 16 hours, the reaction mixture was diluted with aqueous sodium carbonate and extracted three times with pentanes (2 mL). The combined extracts were rinsed with water (2 mL) and dried over sodium sulfate. The crude mixture was purified by column chromatography (1 g silica gel, eluent: 5% ethyl acetate in hexane) giving *cis*-*N*-(α-methylbenzyl)aziridine (**31**, 2.0 mg). Mass spectra of **30**, m/z (relative intensity, %): 385 (M^+, 1), 384 (3), 370 (1), 280 (100), 244 (5), 105 (19), 82 (2), 69 (5), 55 (5).

9.3. GC–MS method for the resolution of *cis*-*N*-(α-methylbenzyl)aziridine derivatives

The GC–MS analysis was performed using a GC–MS (Hewlett-Packard, HP-5090/2) using a ZB-5HT silica capillary column (30 m × 0.32 mm i.d., film thickness 0.25 μm, Zebron) with positive electron ionization (EI). Samples were diluted in *n*-hexanes and injected at a 1:10 split ratio. The injector temperature was set to 250 °C. The oven temperature was held at 80 °C for 1 min and raised to 150 °C at 15 °C/min which was held for 100 min. Then, finally 20 °C/min to 260°C which was held for 1 min. The MS conditions were: solvent - delay time 6 min and scanned mass range (*m*/*z*) 50–500.

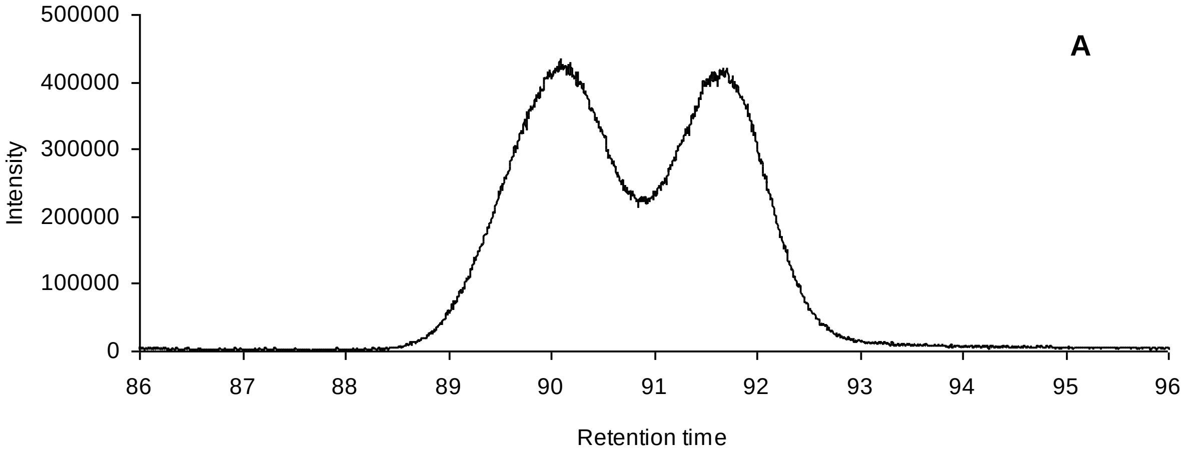

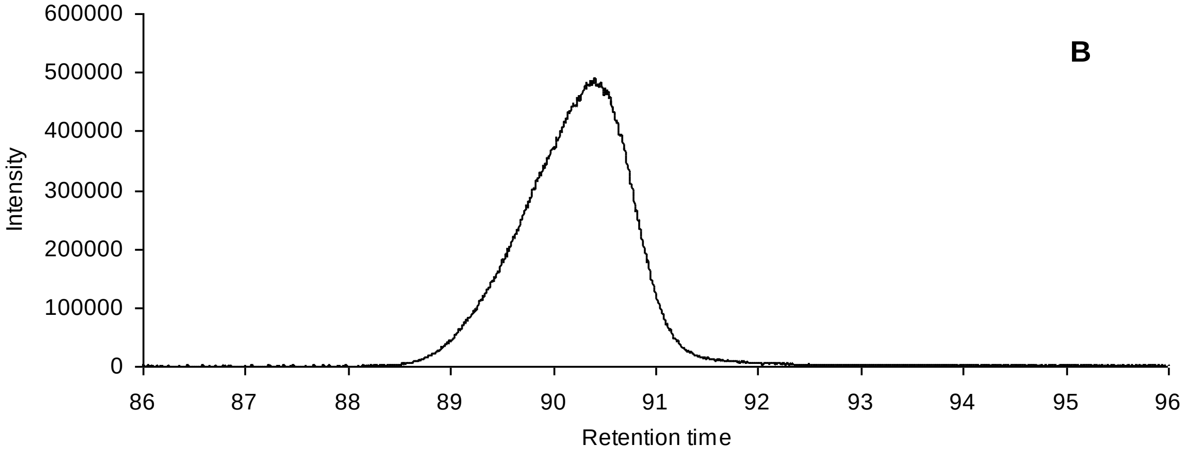

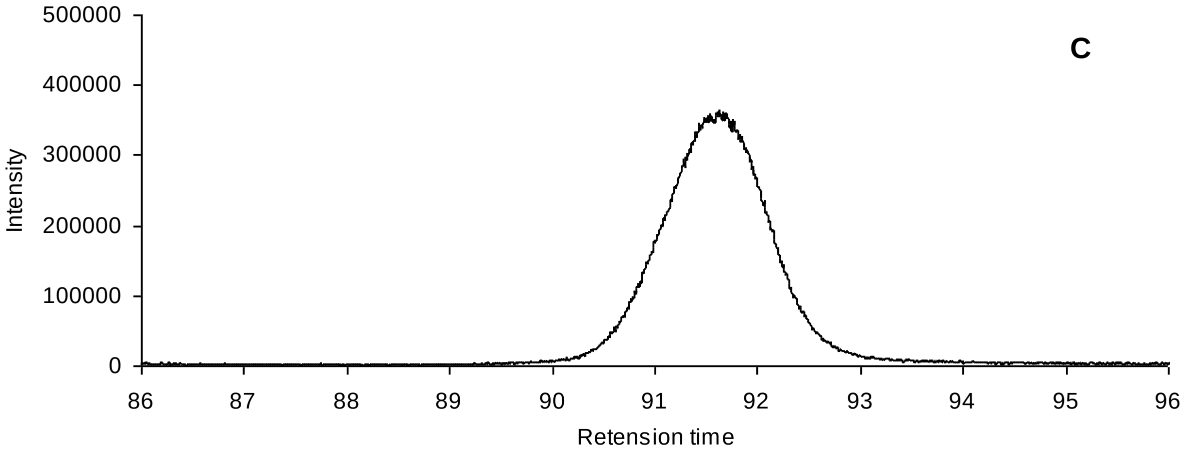

References

1. Dixon, D. J.; Ley, S. V.;Reynolds, D. J. *Chem. Eur. J.*, 2002, *8*, 1621-1036.
2. Dixon, D. J.; Foster, A. C.; Ley, S. V. *Can. J. Chem.*, 2001, *79*, 1668-1680.
3. Drop, A.; Wojtasek, H.; Frąckowiak-Wojtasek, B. *Tetrahedron Lett.*, 2017, *58*, 1453-1455.
4. Kim, S.-G. *Synthesis*, 2009, 2418-2422.
5. Sinha-Bagchi, A.; Sinha, S. C.; Keinan, E. *Tetrahedron: Asymmetry*, 1995, *6*, 2889-2892.
6. Marshall, J. A.; Jablonowski, J. A.; Jiang, H. *J. Org. Chem.*, 1999, *64*, 2152-2154.
7. Bethi, V.; Kattanguru, P.; Fernandes, R. A. *Eur. J. Org. Chem.*, 2014, *2014,* 3249-3255.
8. Prasad, K. R.; Anbarasan, P. *J. Org. Chem.*, 2007, *72*, 3155-3157.
9. Olivier, J. E.; Waters, R. M. *J. Chem. Ecol.*, 1995, *21*, 199-211.
10. Pinnelli, G. R.; Terrado, M.; Hillier, N. K.; Lance, D. R.; Plettner, E. *Eur. J. Org. Chem.*, 2019, 6807-6821.
