# Supplementary material for: Synthesis of disparlure and monachalure enantiomers from 2,3-butanediacetals
Source: Beilstein J Org Chem. 2020 Apr 3;16:616–20. doi: 10.3762/bjoc.16.57 (PMC7136567; doi:10.3762/bjoc.16.57)

BEILSTEIN JOURNAL OF ORGANIC CHEMISTRY

Supporting Information

for

Synthesis of disparlure and monachalure enantiomers from 2,3-butanediacetals

Adam Drop, Hubert Wojtasek and Bożena Frąckowiak-Wojtasek

Beilstein J. Org. Chem. **2020,** *16,* 616–620. doi:10.3762/bjoc.16.57

Copies of ^{1}H NMR and ^{13}C NMR spectra

License and Terms: This is a supporting information file under the terms of the Creative Commons Attribution License (http://creativecommons.org/licenses/by/4.0). Please note that the reuse, redistribution and reproduction in particular requires that the authors and source are credited.
The license is subject to the *Beilstein Journal of Organic Chemistry* terms and conditions: (https://www.beilstein-journals.org/bjoc)

Table of contents

1.	^{1}H NMR, ^{13}C NMR spectra of (2*R*,3*R*,5*R*,6*R*)-5,6-dimethoxy-5,6-dimethyl-1,4-dioxane-2,3-dimethyl dicarboxylate (**9**)	S3,S4
2.	^{1}H NMR, ^{13}C NMR spectra of methyl (2*S*,3*R*,5*R*,6*R*)-3-formyl-5,6-dimethoxy-5,6-dimethyl-1,4-dioxane-2-carboxylate (**15**)	S5,S6
3.	^{1}H NMR, ^{13}C NMR spectra of [(2*S*,3*R*,5*R*,6*R*)-3-(hydroxymethyl)-5,6-dimethoxy-5,6-dimethyl-1,4-dioxan-2-yl] methanol (**17**)	S7,S8
4.	^{1}H NMR, ^{13}C NMR spectra of (2*S*,3*R*,5*R*,6*R*)-3-*tert*-butyldimethylsilyloxymethyl-2-hydroxymethyl-5,6-dimethoxy-5,6-dimethyl-14-dioxane (**18**)	S9,S10
5.	^{1}H NMR, ^{13}C NMR spectra of (2*R*,3*R*,5*R*,6*R*)-3-[[*tert*-butyl(dimethyl)silyl]oxymethyl]-5,6-dimethoxy-5,6-dimethyl-1,4-dioxane-2-carbaldehyde (**19**)	S11,S12
6.	^{1}H NMR, ^{13}C NMR spectra of (−)-*tert*-butyl-[[(2*R*,3*S*,5*R*,6*R*)-3-decyl-5,6-dimethoxy-5,6-dimethyl-1,4-dioxan-2-yl] methoxy]-dimethyl-silane (**20**)	S13,S14
7.	^{1}H NMR, ^{13}C NMR spectra of (−)-(2*S*,3*S*,5*R*,6*R*)-3-decyl-5,6-dimethoxy-5,6-dimethyl-1,4-dioxane-2-carbaldehyde (**21**)	S15,S16
8.	^{1}H NMR, ^{13}C NMR spectra of (+)-(2*R*,3*R*,5*S*,6*R*)-5-decyl-2,3-dimethoxy-2,3-dimethyl-6-(5-methylhexyl)-1,4-dioxane (**22**)	S17,S18
9.	^{1}H NMR, ^{13}C NMR spectra of (+)-(2*R*,3*R*,5*S*,6*R*)-5-decyl-6-hexyl-2,3-dimethoxy-2,3-dimethyl-1,4-dioxane (**23**)	S19,S20
10.	^{1}H NMR, ^{13}C NMR spectra of (+)-(7*R*,8*S*)-2-methyloctadecane-7,8-diol (**5**)	S21,S22
11.	^{1}H NMR, ^{13}C NMR spectra of (+)-(7*R*,8*S*)-octadecane-7,8-diol (**6**)	S23,S24
12.	^{1}H NMR, ^{13}C NMR spectra of (+)-disparlure (**1**)	S25,S26
13.	^{1}H NMR, ^{13}C NMR spectra of (+)-monachalure (**2**)	S27,S28
14.	^{1}H NMR, ^{13}C NMR spectra of (−)-*tert*-butyl-[[(2*R*,3*S*,5*R*,6*R*)-5,6-dimethoxy-5,6-dimethyl-3-(5-methylhexyl)-1,4-dioxan-2-yl]methoxy]-dimethyl-silane (**24**)	S29,S30
15.	^{1}H NMR, ^{13}C NMR spectra of (−)-*tert*-butyl-[[(2*R*,3*S*,5*R*,6*R*)-3-hexyl-5,6-dimethoxy-5,6-dimethyl-1,4-dioxan-2-yl]methoxy]-dimethyl-silane (**25**)	S31,S32

16. ^{1}H NMR, ^{13}C NMR spectra of (−)-(2*S*,3*S*,5*R*,6*R*)-5,6-dimethoxy-5,6-dimethyl-3-(5-methylhexyl)-1,4-dioxane-2-carbaldehyde (**26**) S33,S34

17. ^{1}H NMR spectrum (−)-(2*S*,3*S*,5*R*,6*R*)-3-hexyl-5,6-dimethoxy-5,6-dimethyl-1,4-dioxane-2-carbaldehyde (**27**) S35

18. ^{1}H NMR, ^{13}C NMR spectra of (−)-(2*R*,3*R*,5*R*,6*S*)-5-decyl-2,3-dimethoxy-2,3-dimethyl-6-(5-methylhexyl)-1,4-dioxane (**28**) S36,S37

19. ^{1}H NMR, ^{13}C NMR spectra of (−)-(2*R*,3*R*,5*R*,6*S*)-5-decyl-6-hexyl-2,3-dimethoxy-2,3-dimethyl-1,4-dioxane (**29**) S38,S39

20. ^{1}H NMR, ^{13}C NMR spectra of (−)-(7*S*,8*R*)-2-methylnonadecane-7,8-diol (**7**) S40,S41

21. ^{1}H NMR, ^{13}C NMR spectra of (−)-(7*S*,8*R*)-nonadecane-7,8-diol (**8**) S42,S43

22. ^{1}H NMR, ^{13}C NMR spectra of (−)-disparlure (**3**) S44,S45

23. ^{1}H NMR, ^{13}C NMR spectra of (−)-monachalure (**4**) S46,S47

^{1}H NMR spectrum of **9**

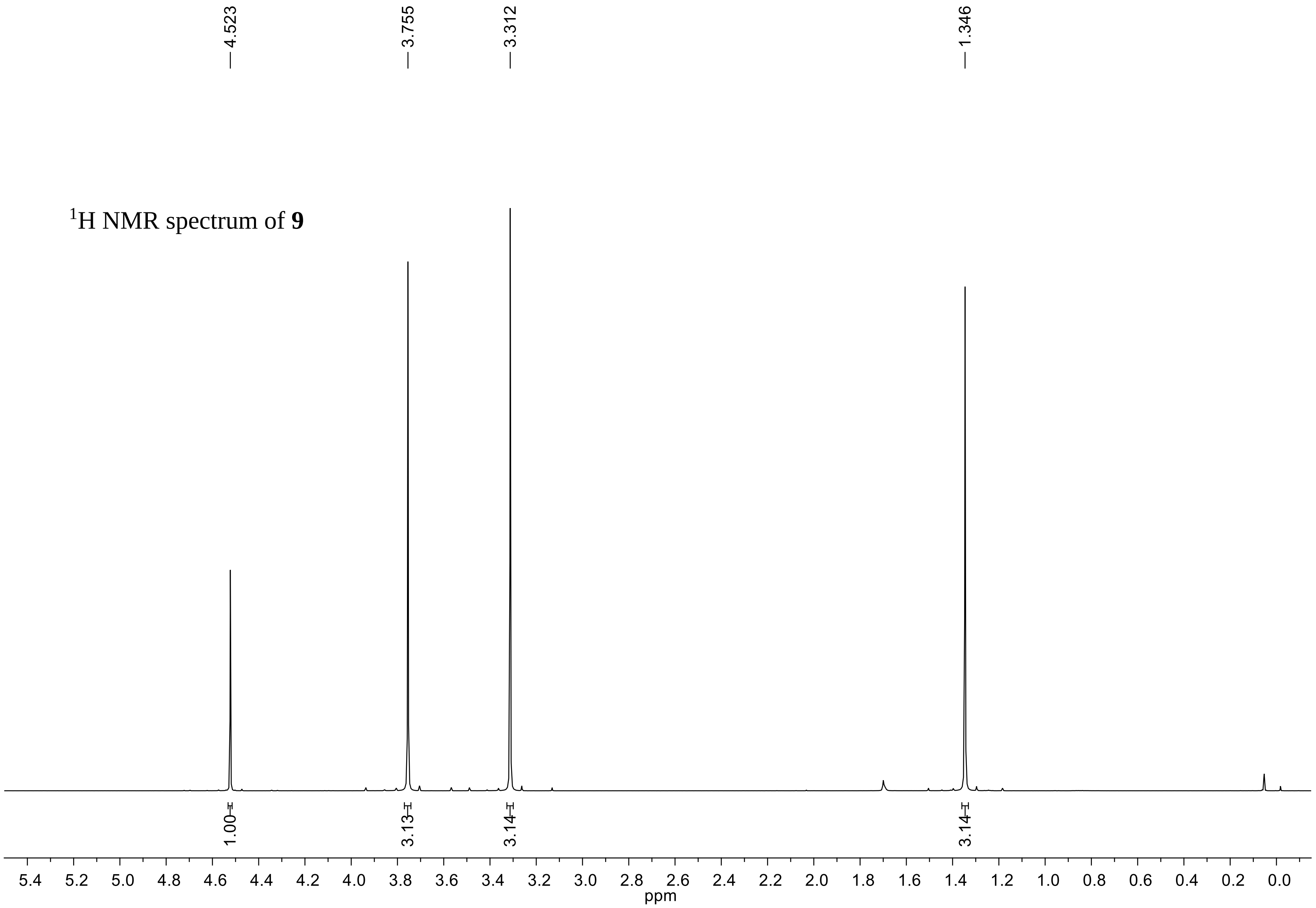

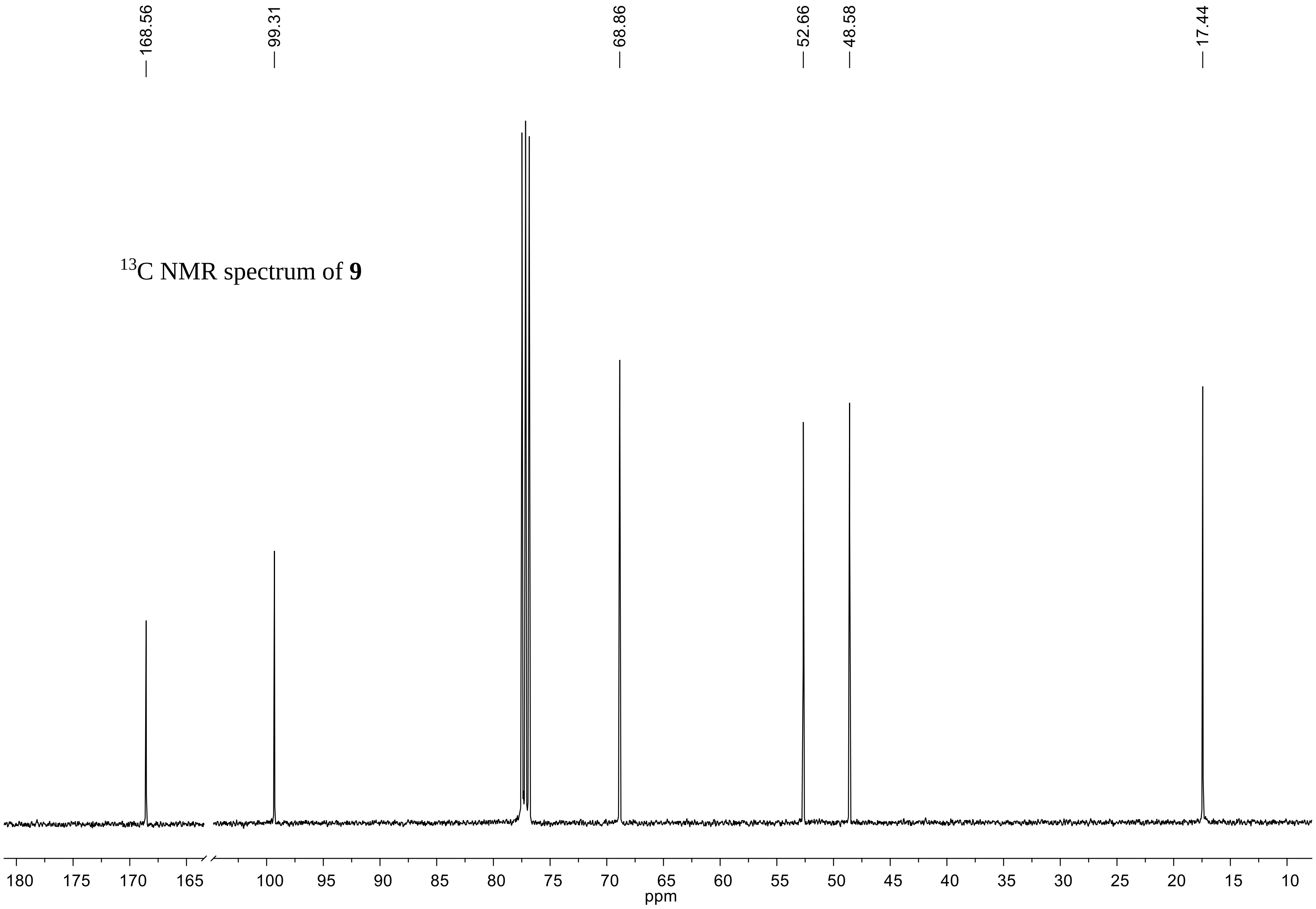

^{13}C NMR spectrum of **9**

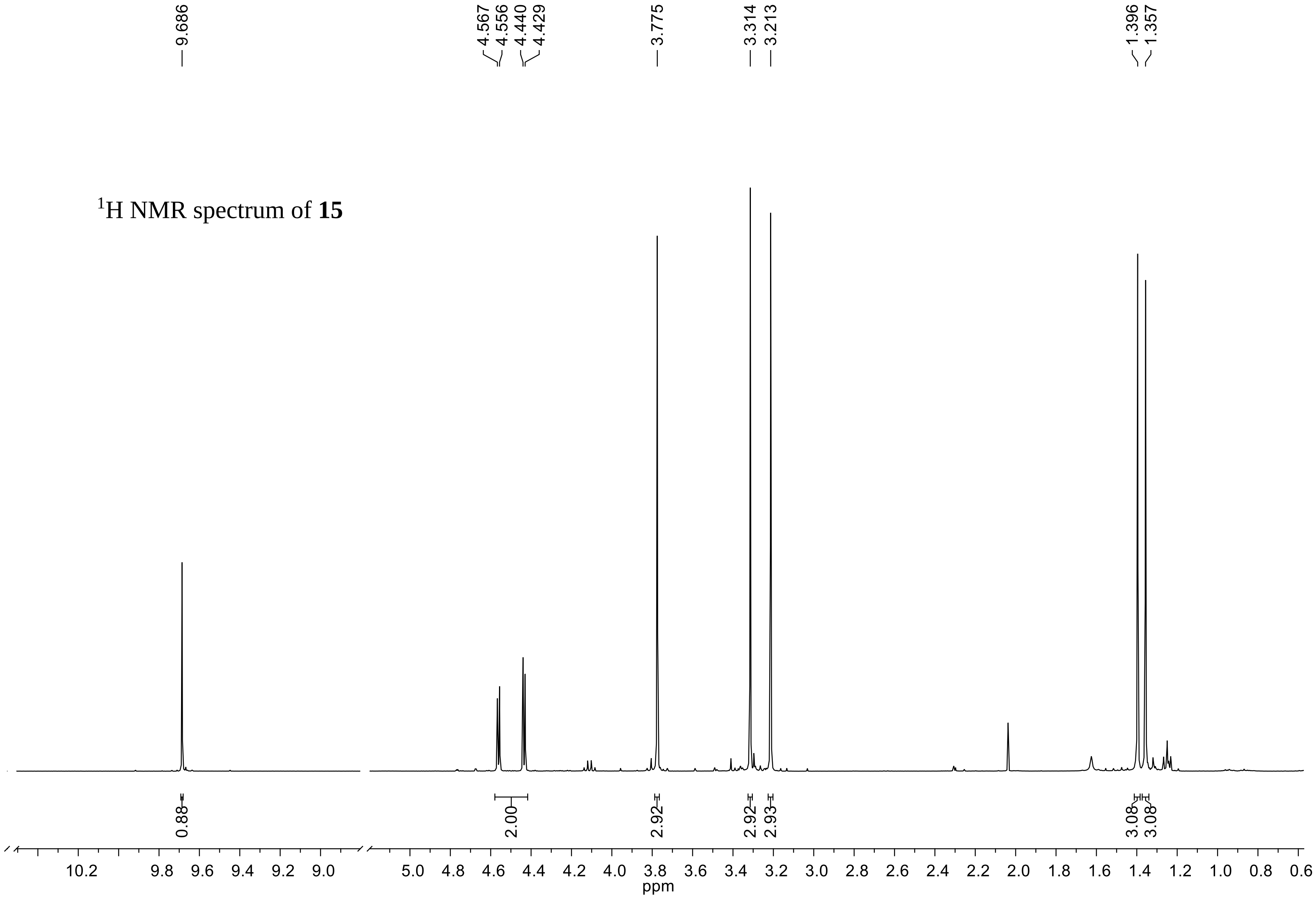
9.686
4.567
4.556
4.440
4.429
3.775
3.314
3.213
1.396
1.357
^{1}H NMR spectrum of **15**
0.88
2.00
2.92
2.92
2.93
3.08
3.08
10.2 9.8 9.6 9.4 9.2 9.0 5.0 4.8 4.6 4.4 4.2 4.0 3.8 3.6 3.4 3.2 3.0 2.8 2.6 2.4 2.2 2.0 1.8 1.6 1.4 1.2 1.0 0.8 0.6
ppm

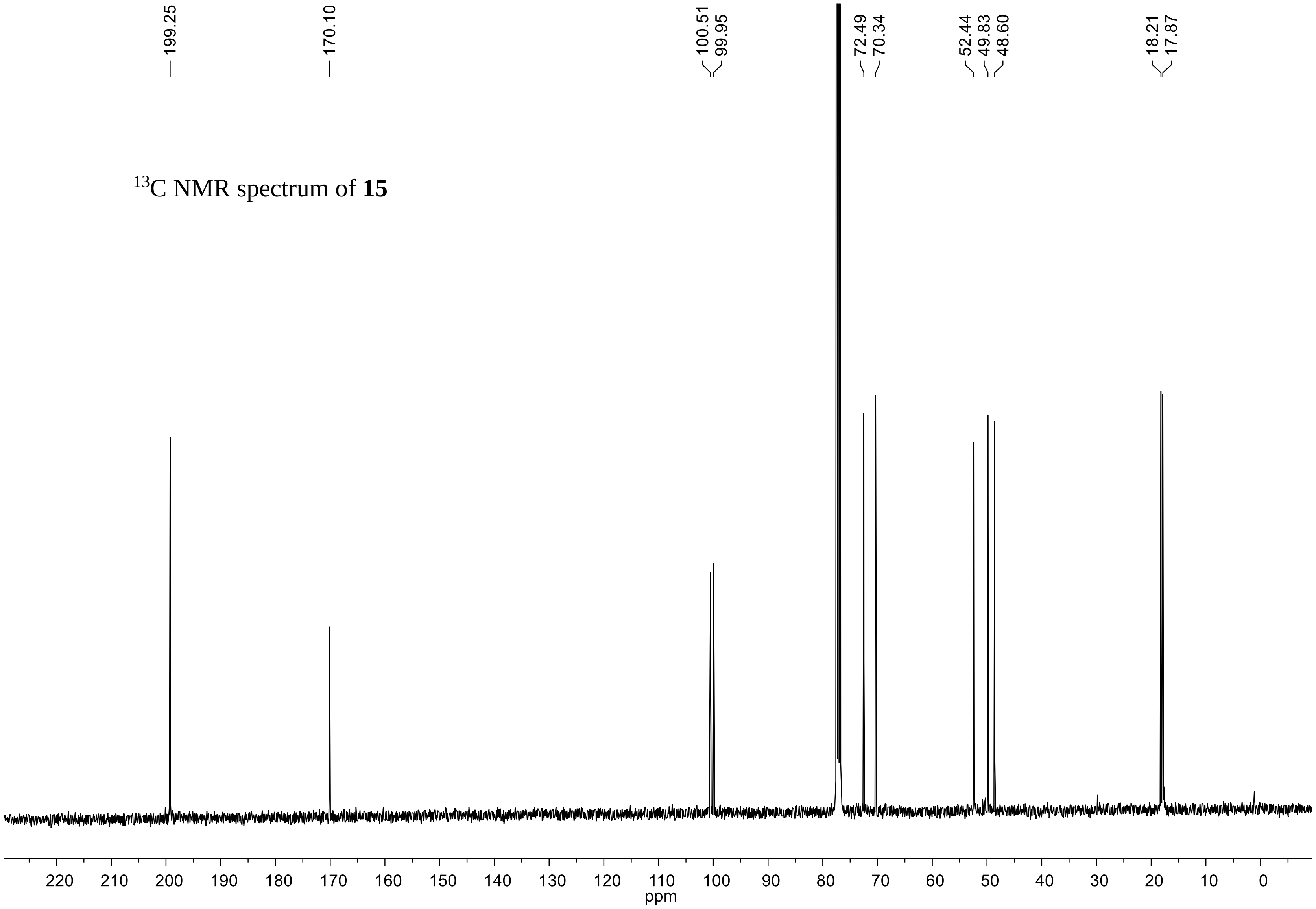

^{13}C NMR spectrum of **15**

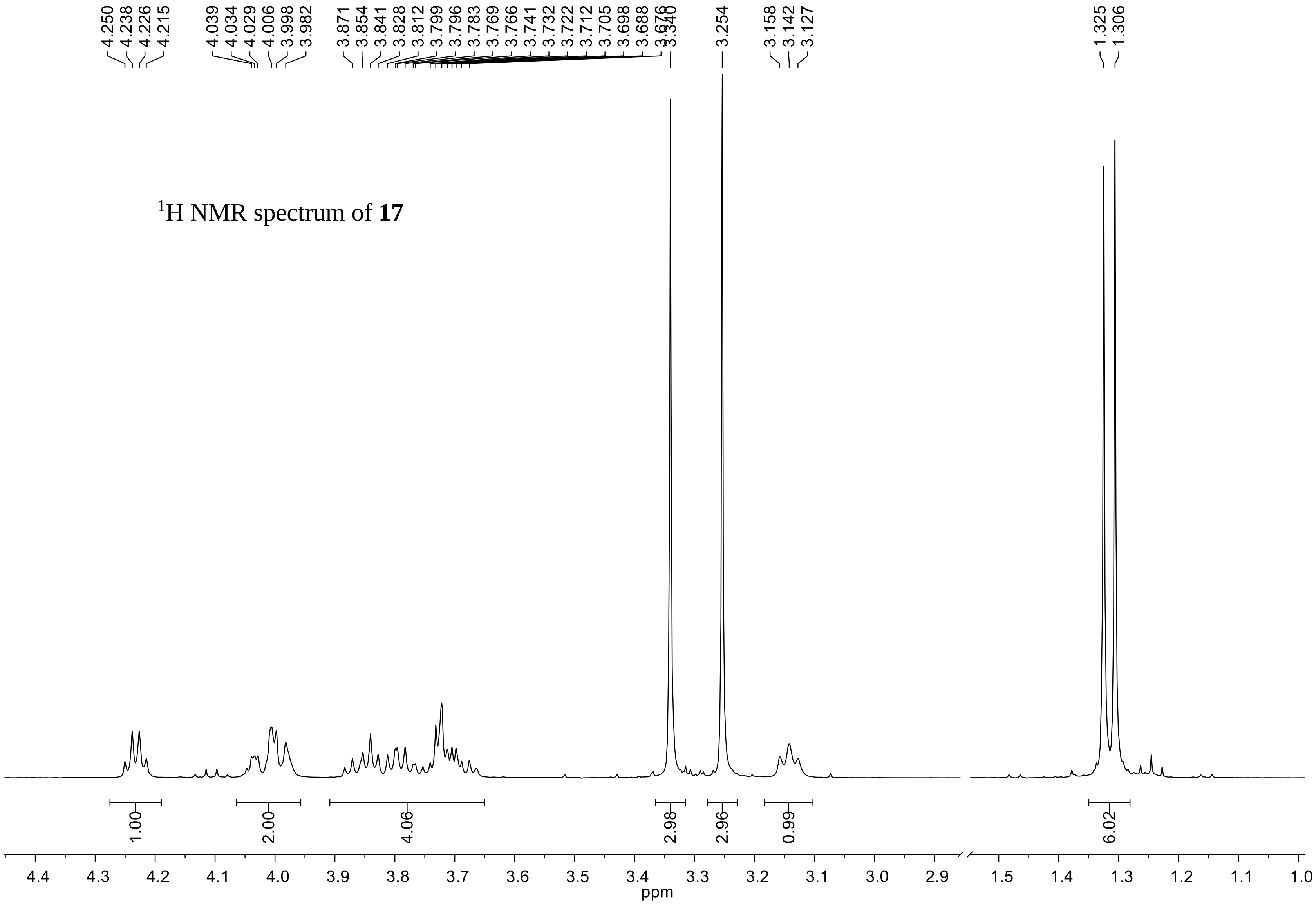

^{1}H NMR spectrum of **17**
4.250
4.238
4.226
4.215
4.039
4.034
4.029
4.006
3.998
3.982
3.871
3.854
3.841
3.828
3.812
3.799
3.796
3.783
3.769
3.766
3.741
3.732
3.722
3.712
3.705
3.698
3.688
3.676
3.340
3.254
3.158
3.142
3.127
1.325
1.306
1.00
2.00
4.06
2.98
2.96
0.99
6.02
ppm

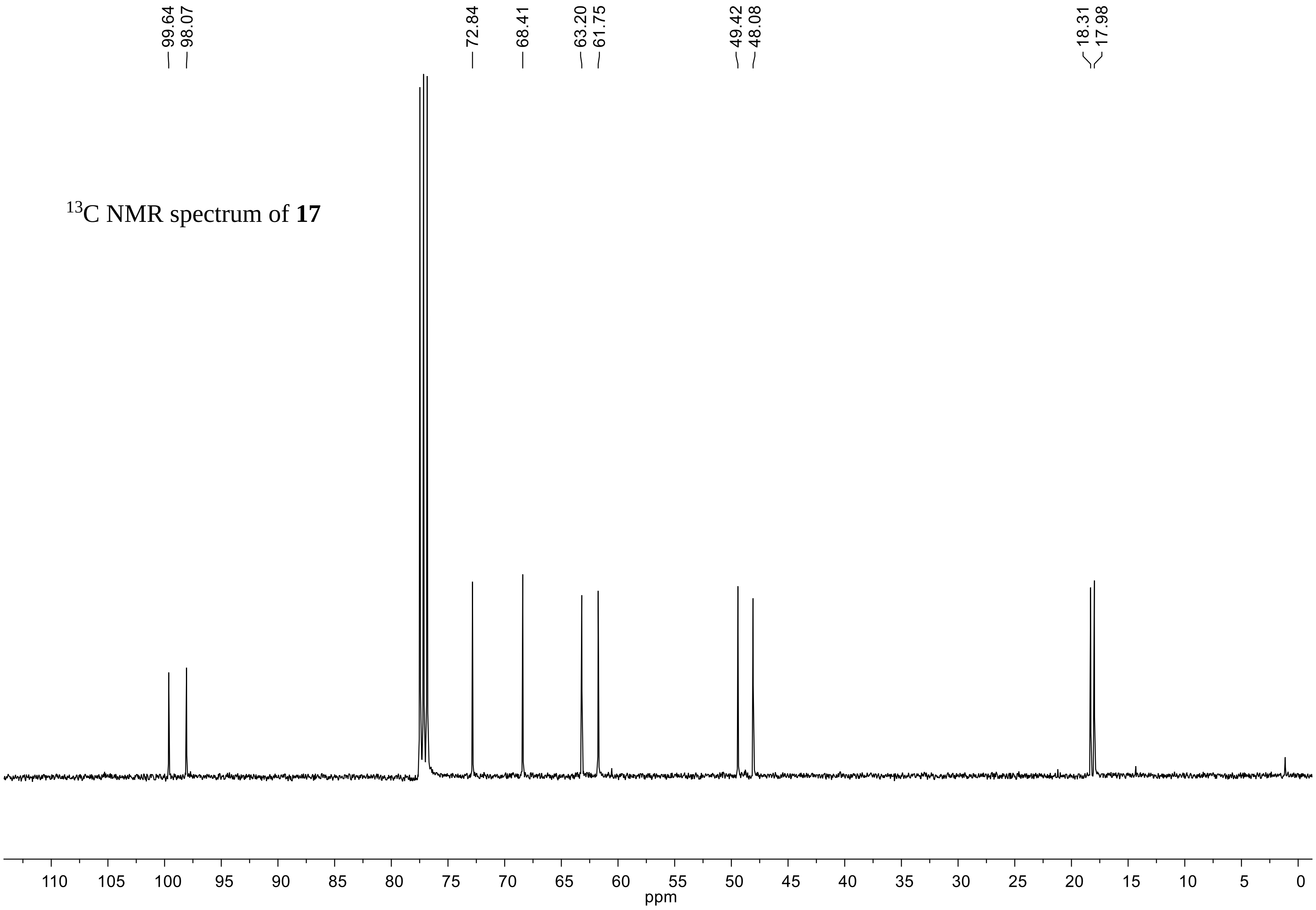

^{13}C NMR spectrum of **17**

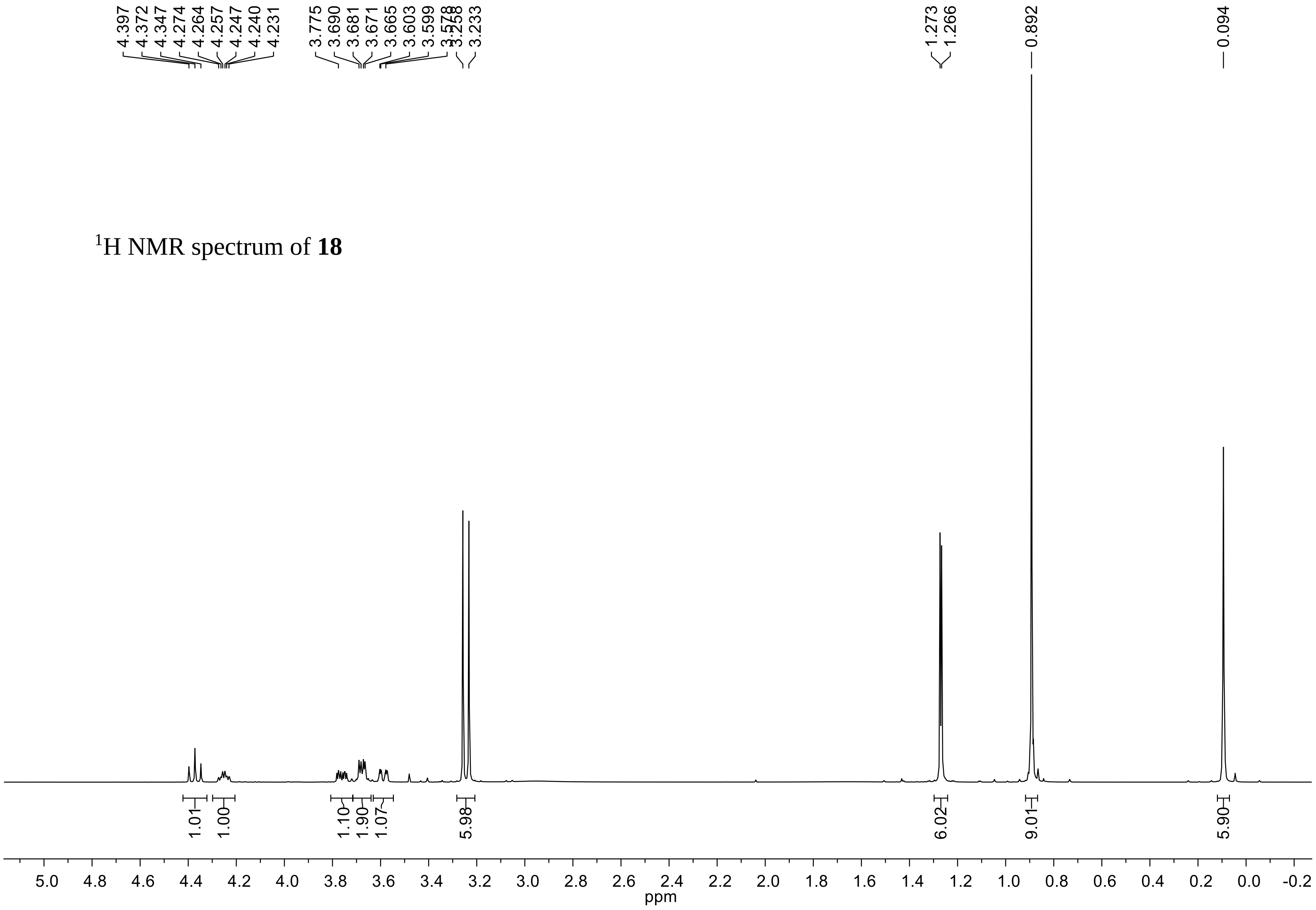

^{1}H NMR spectrum of **18**

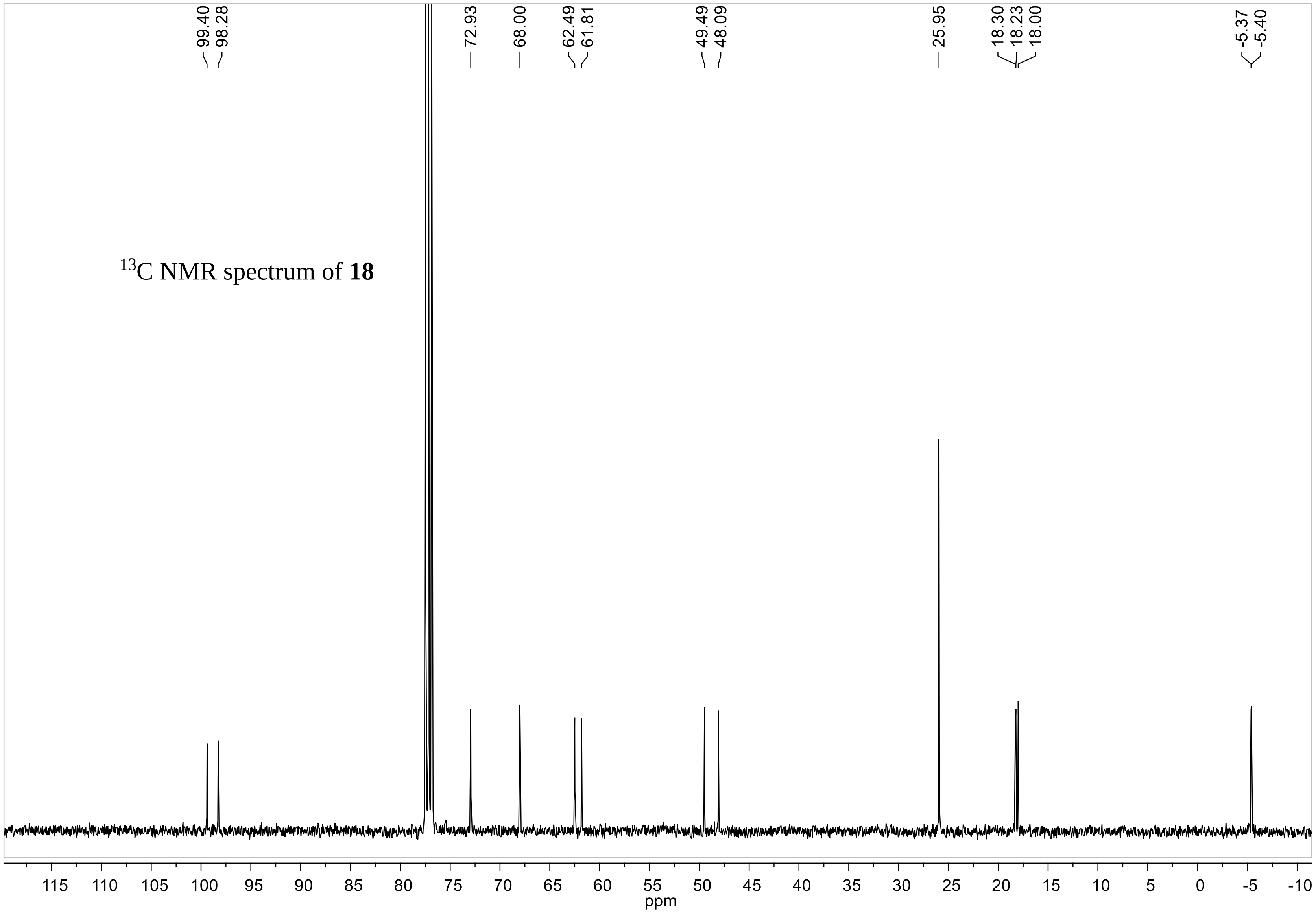

^{13}C NMR spectrum of **18**

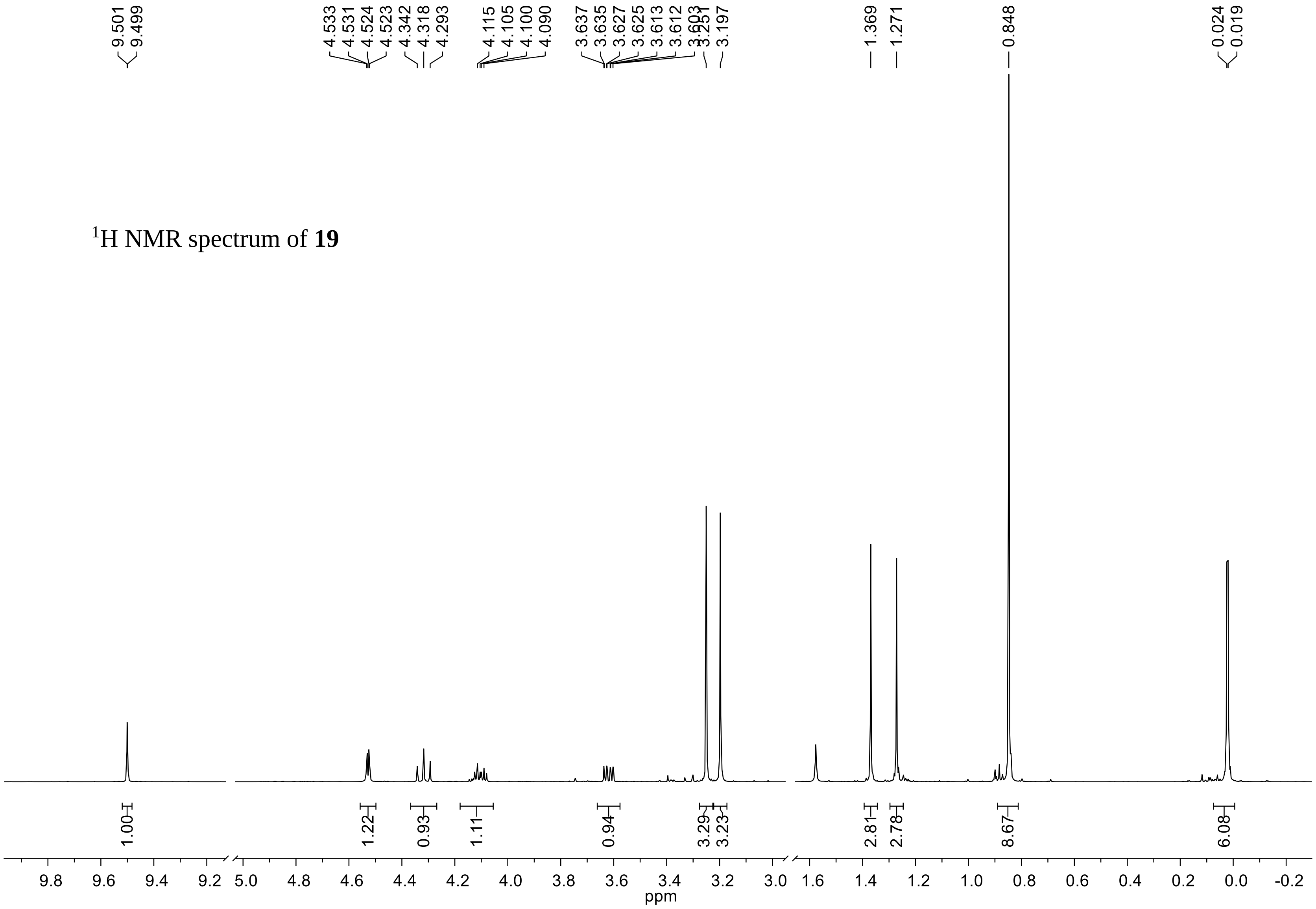

^{1}H NMR spectrum of **19**

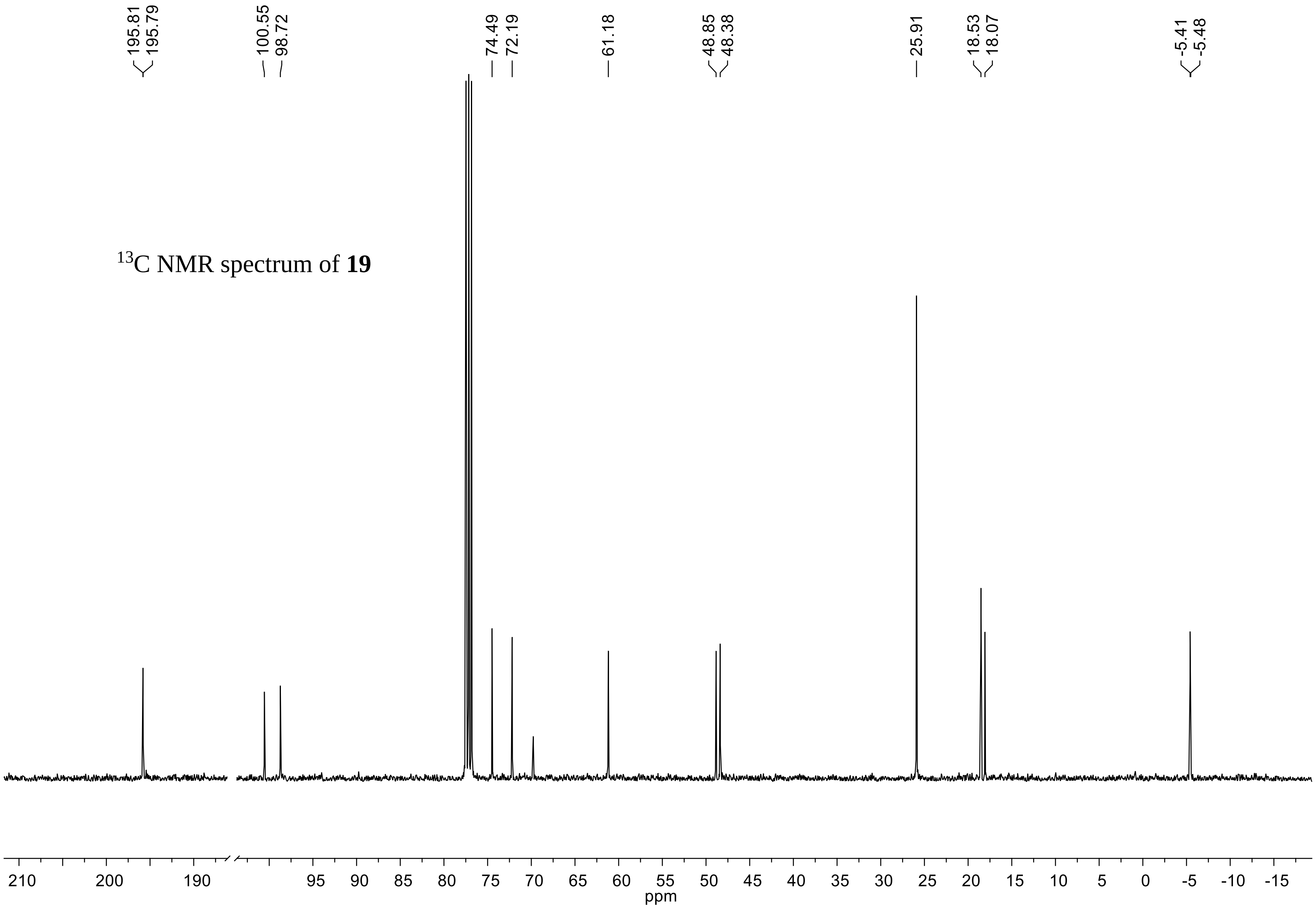

^{13}C NMR spectrum of **19**

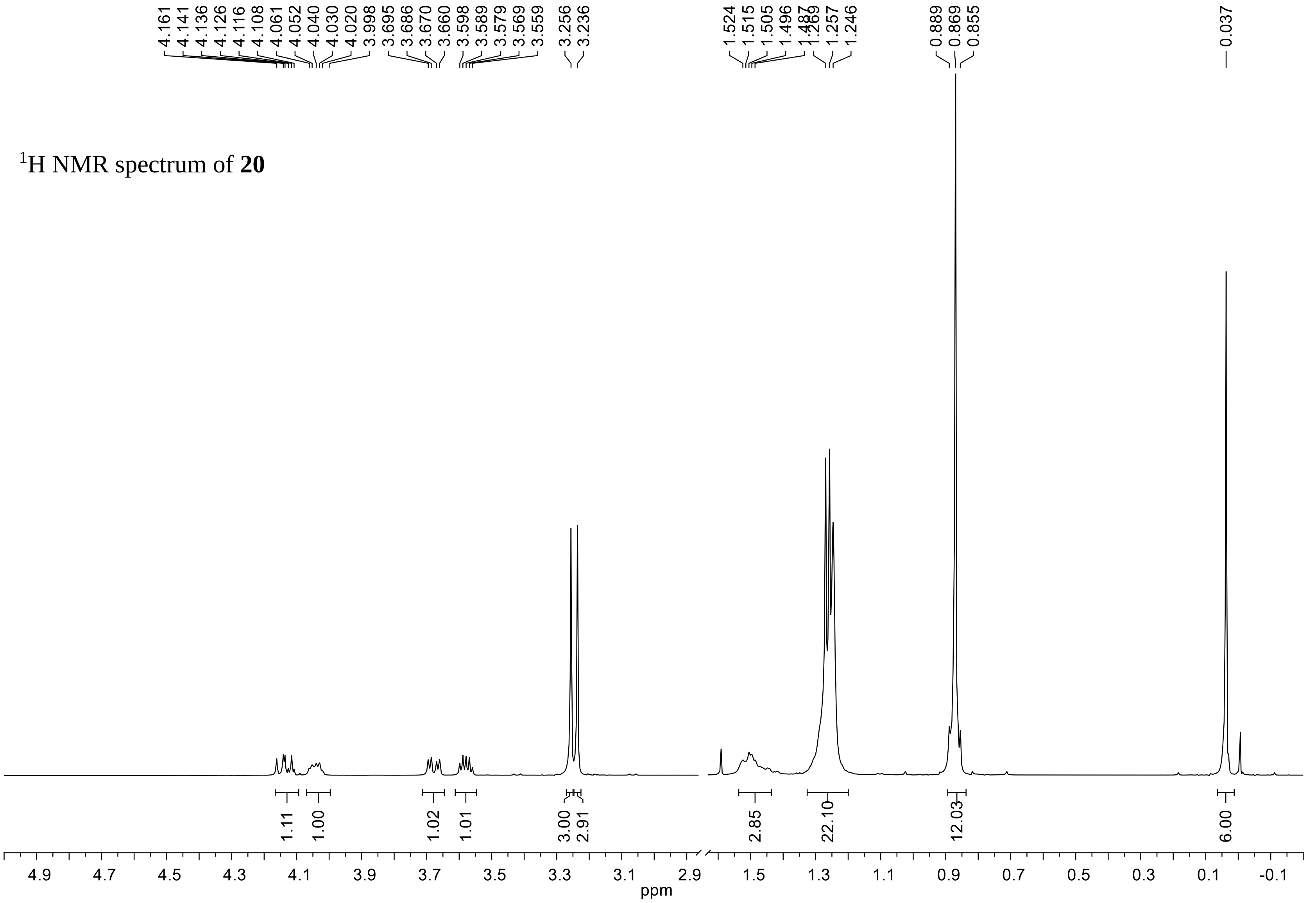

^{1}H NMR spectrum of **20**

^{13}C NMR spectrum of **20**

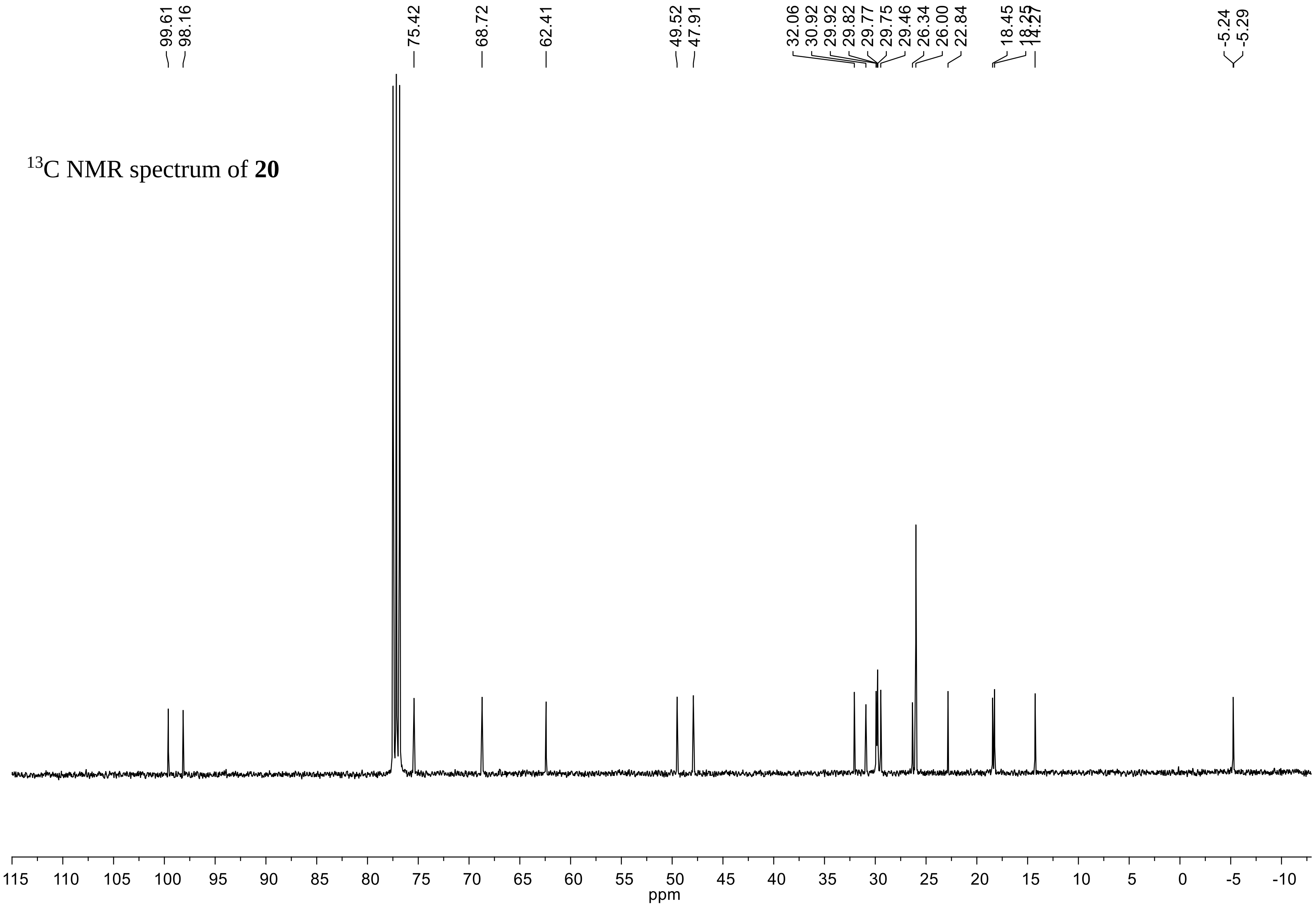

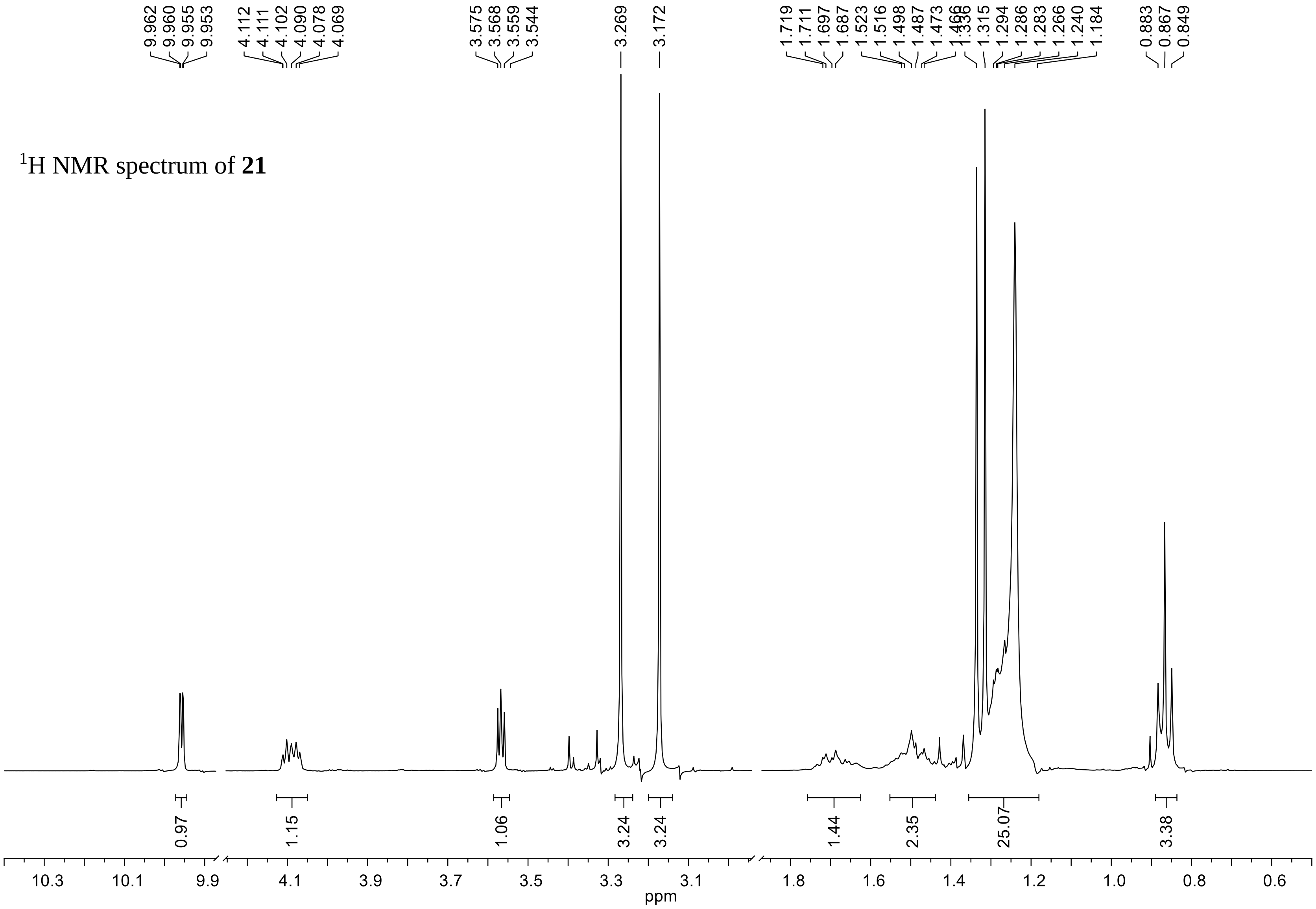

^{1}H NMR spectrum of **21**

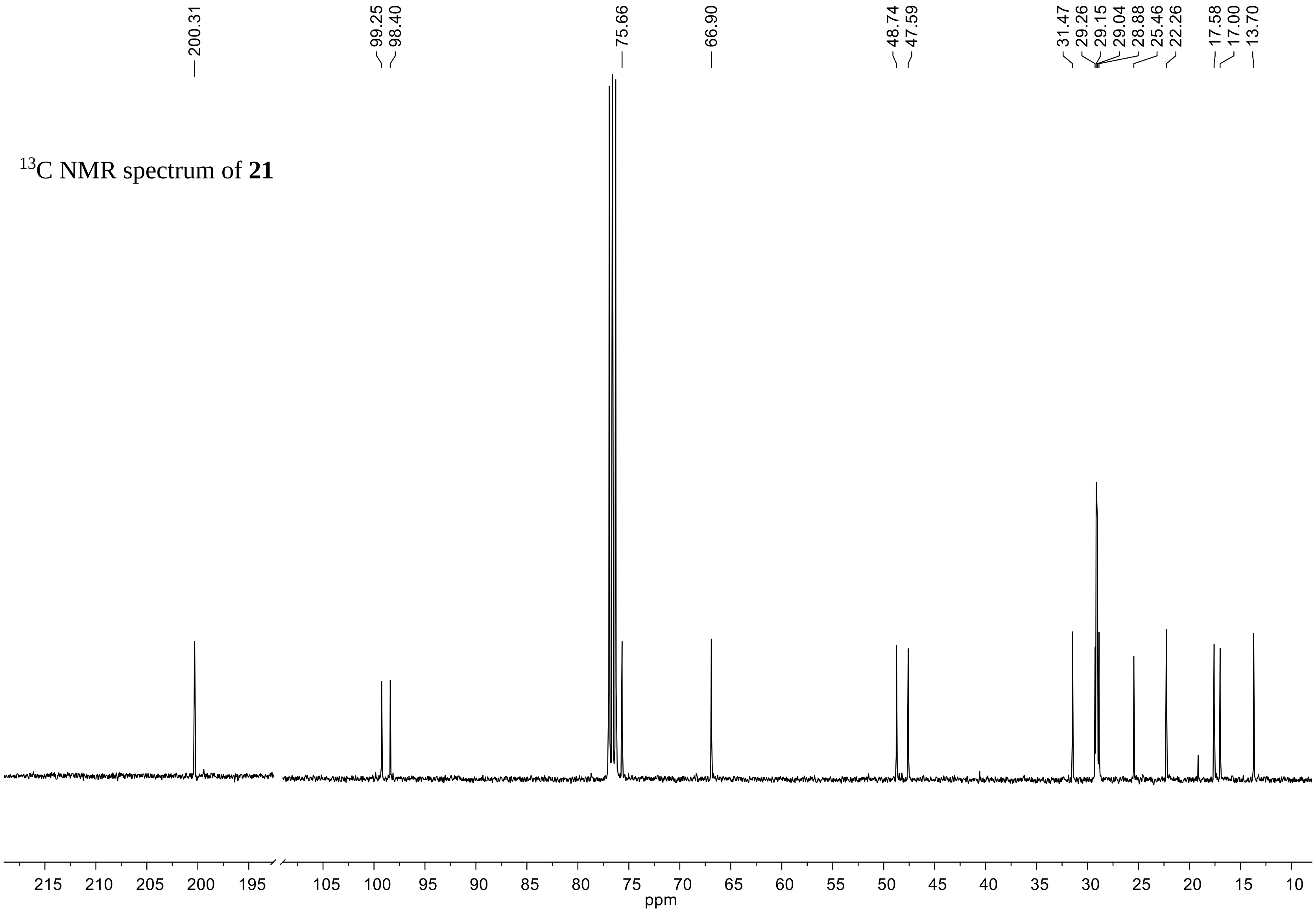

^{13}C NMR spectrum of **21**

^{1}H NMR spectrum of **22**

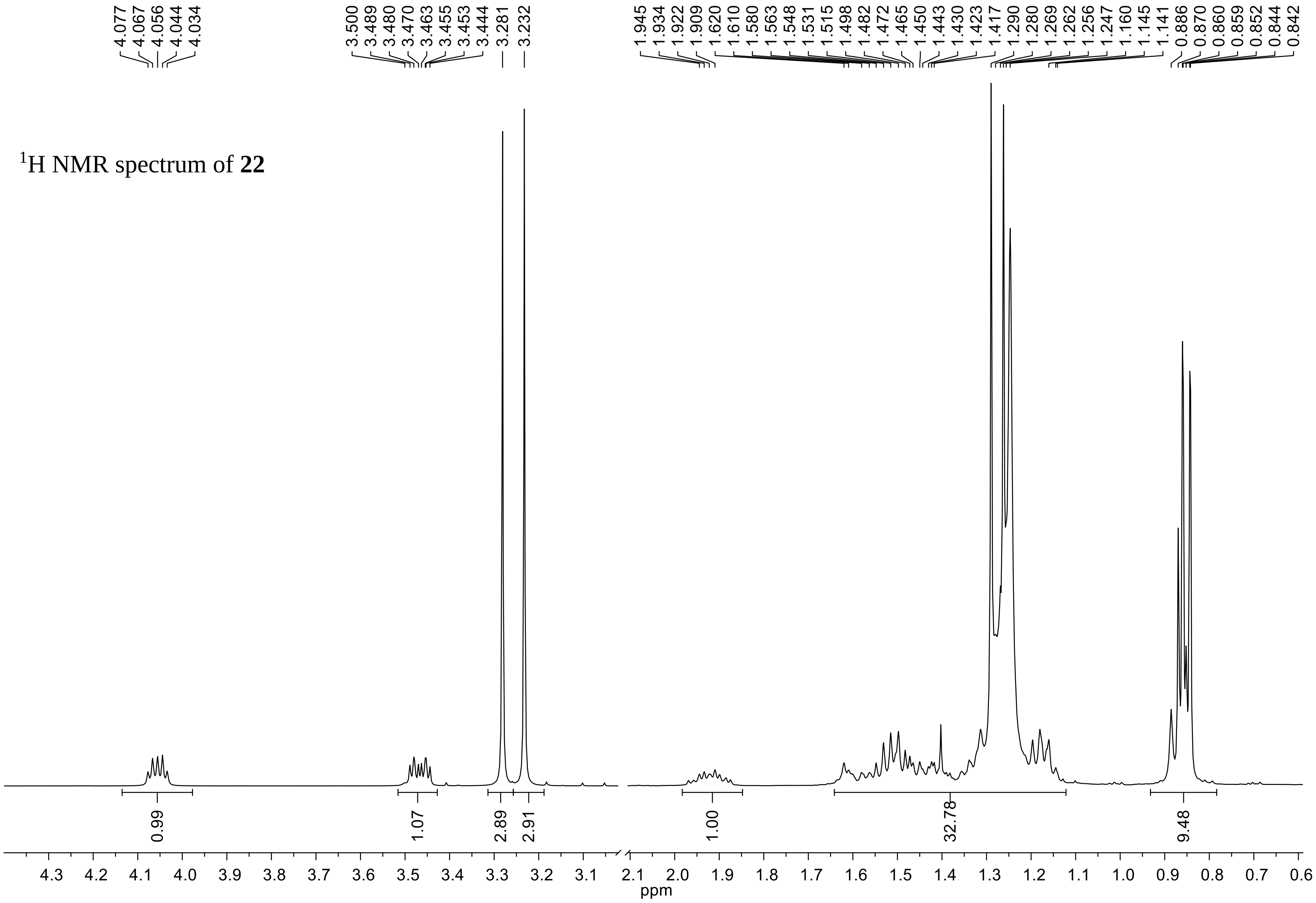

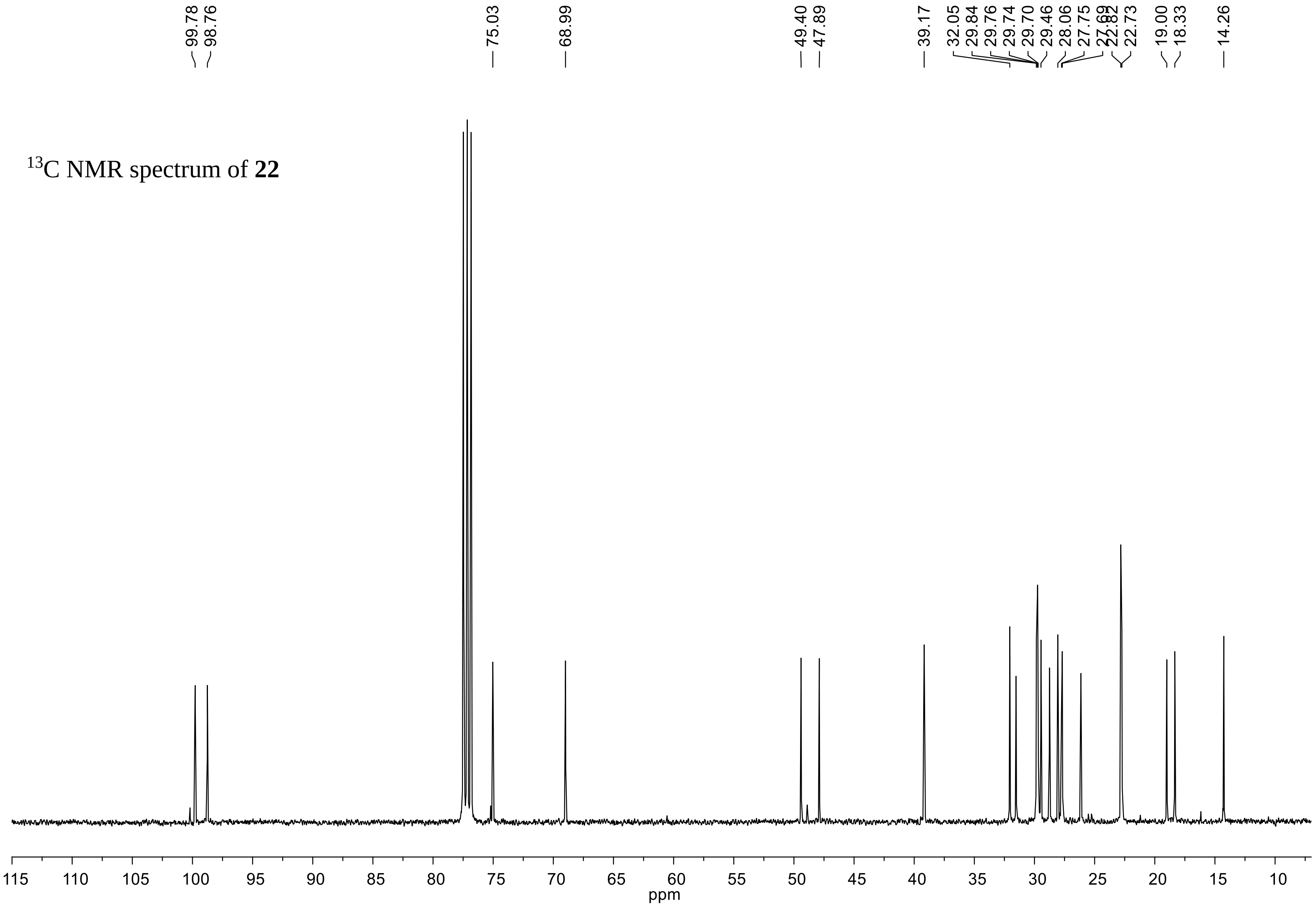

99.78
98.76
75.03
68.99
49.40
47.89
39.17
32.05
29.84
29.76
29.74
29.70
29.46
28.06
27.75
22.82
22.73
19.00
18.33
14.26
115 110 105 100 95 90 85 80 75 70 65 60 55 50 45 40 35 30 25 20 15 10
ppm

^{1}H NMR spectrum of **23**

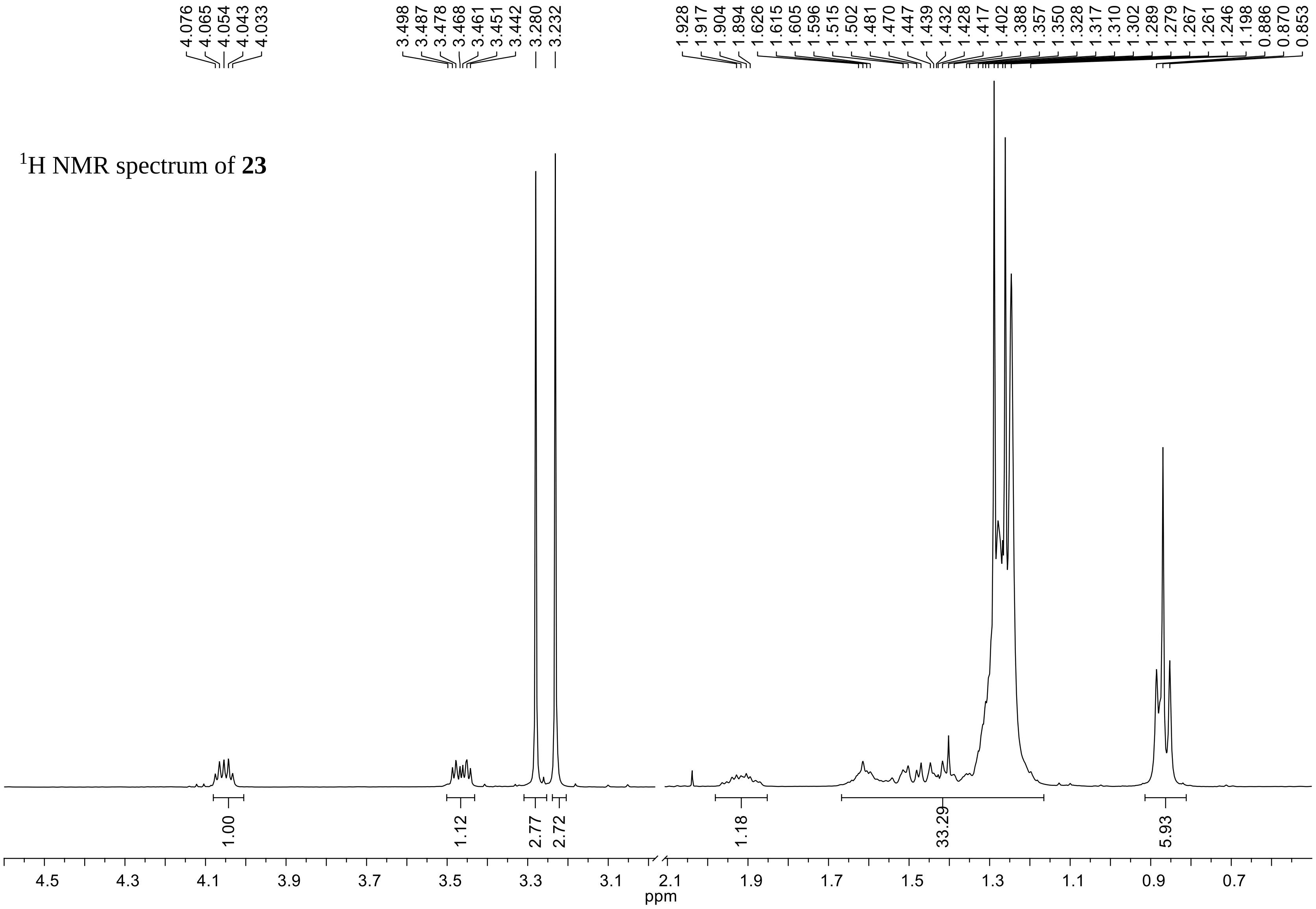

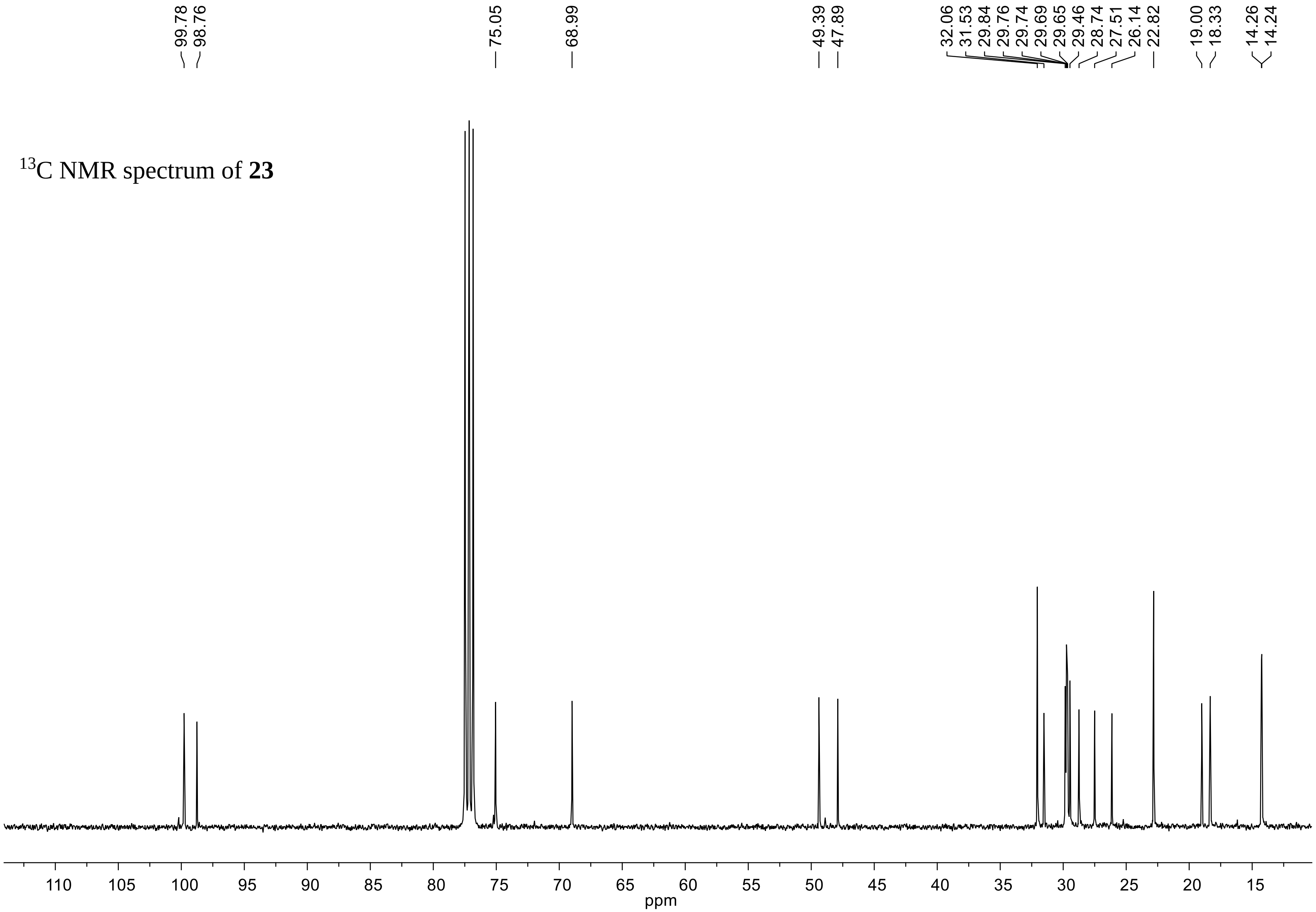

^{13}C NMR spectrum of **23**
99.78
98.76
75.05
68.99
49.39
47.89
32.06
31.53
29.84
29.76
29.74
29.69
29.65
29.46
28.74
27.51
26.14
22.82
19.00
18.33
14.26
14.24
110 105 100 95 90 85 80 75 70 65 60 55 50 45 40 35 30 25 20 15
ppm

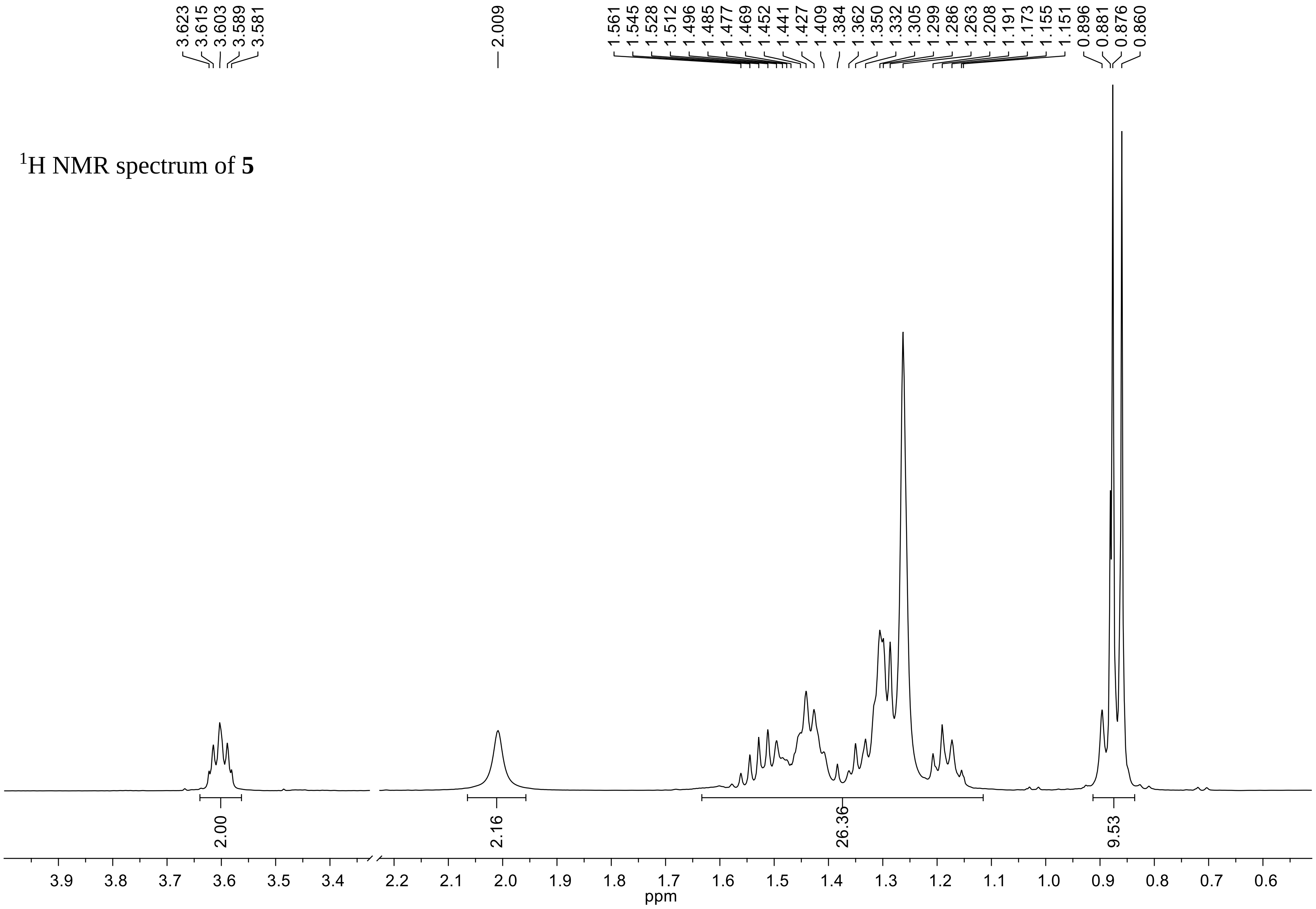

^{1}H NMR spectrum of **5**
3.623
3.615
3.603
3.589
3.581
2.009
1.561
1.545
1.528
1.512
1.496
1.485
1.477
1.469
1.452
1.441
1.427
1.409
1.384
1.362
1.350
1.332
1.305
1.299
1.286
1.263
1.208
1.191
1.173
1.155
1.151
0.896
0.881
0.876
0.860
2.00
2.16
26.36
9.53
ppm

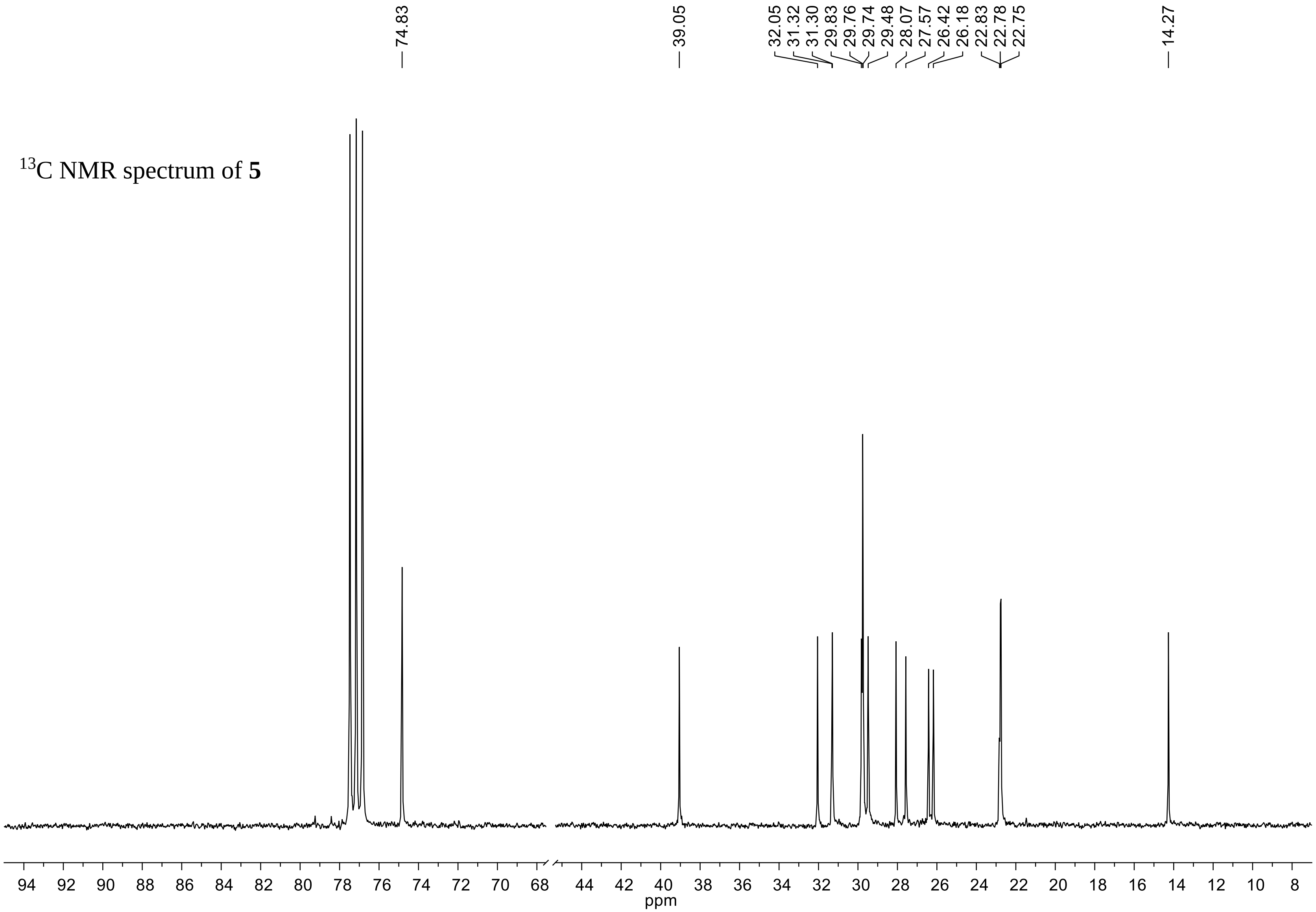
74.83
39.05
32.05
31.32
31.30
29.83
29.76
29.74
29.48
28.07
27.57
26.42
26.18
22.83
22.78
22.75
14.27
ppm

^{1}H NMR spectrum of **6**

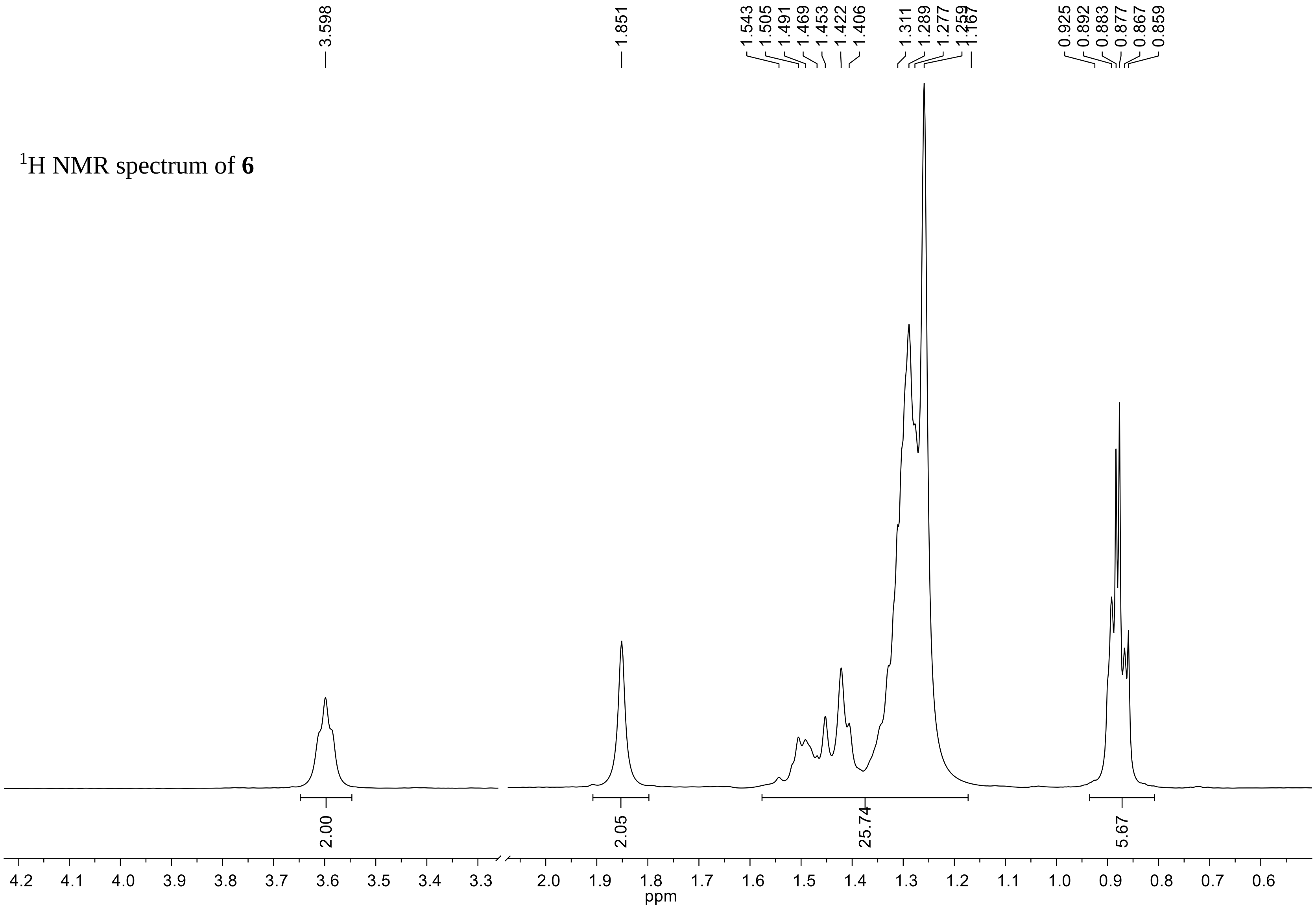

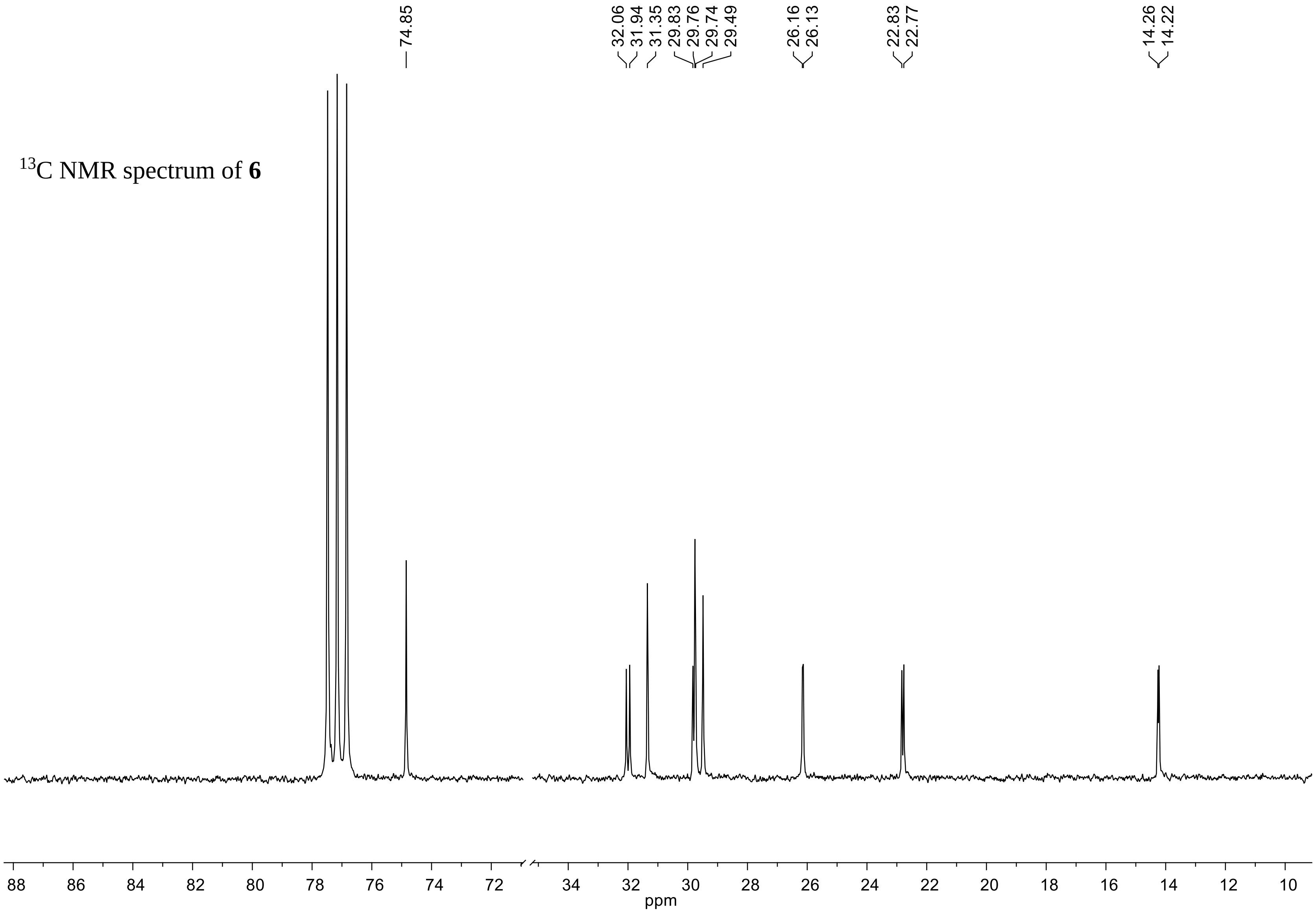
13C NMR spectrum of 6
74.85
32.06
31.94
31.35
29.83
29.76
29.74
29.49
26.16
26.13
22.83
22.77
14.26
14.22
88
86
84
82
80
78
76
74
72
34
32
30
28
26
24
22
20
18
16
14
12
10
ppm

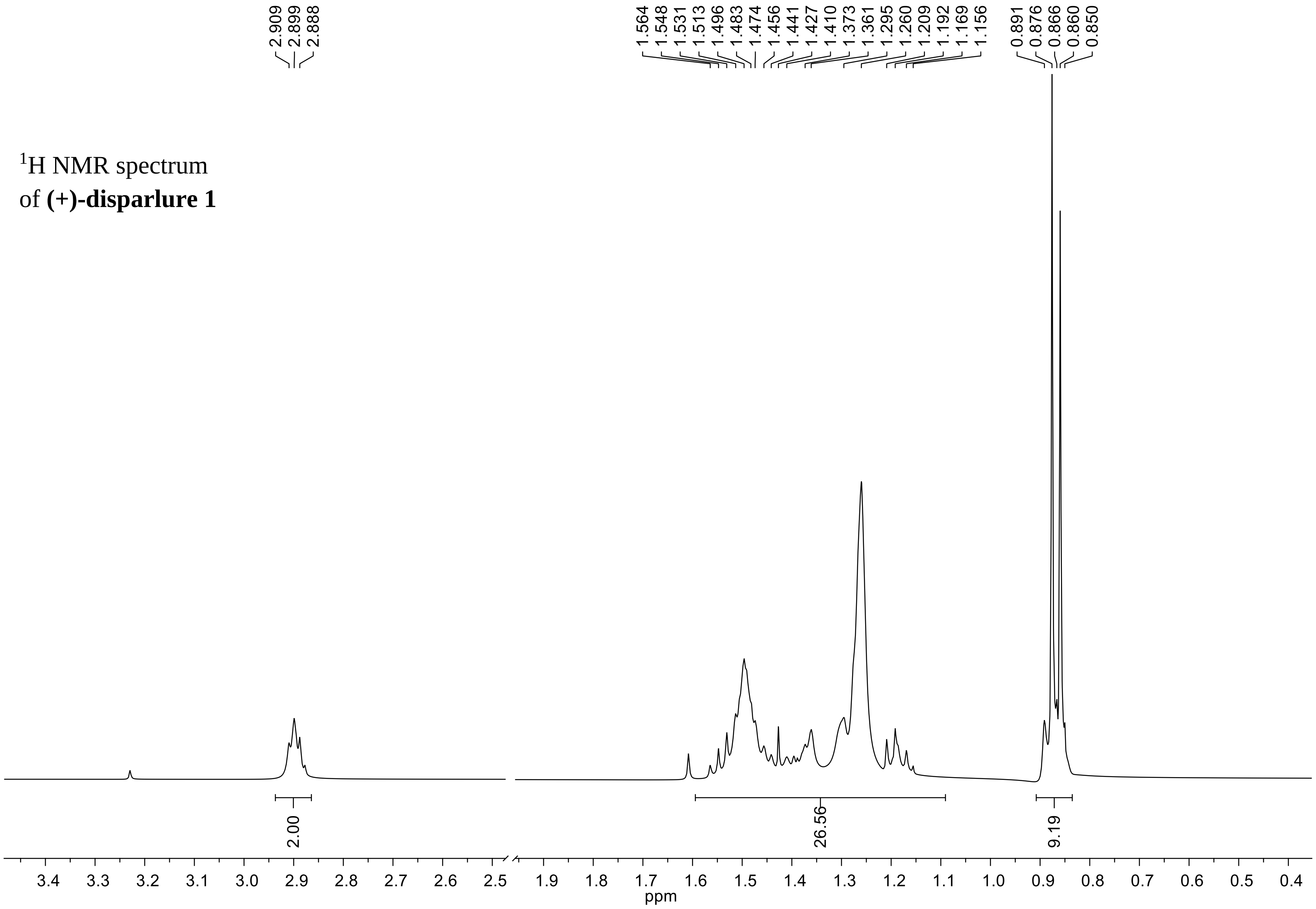

^{1}H NMR spectrum
of **(+)-disparlure 1**

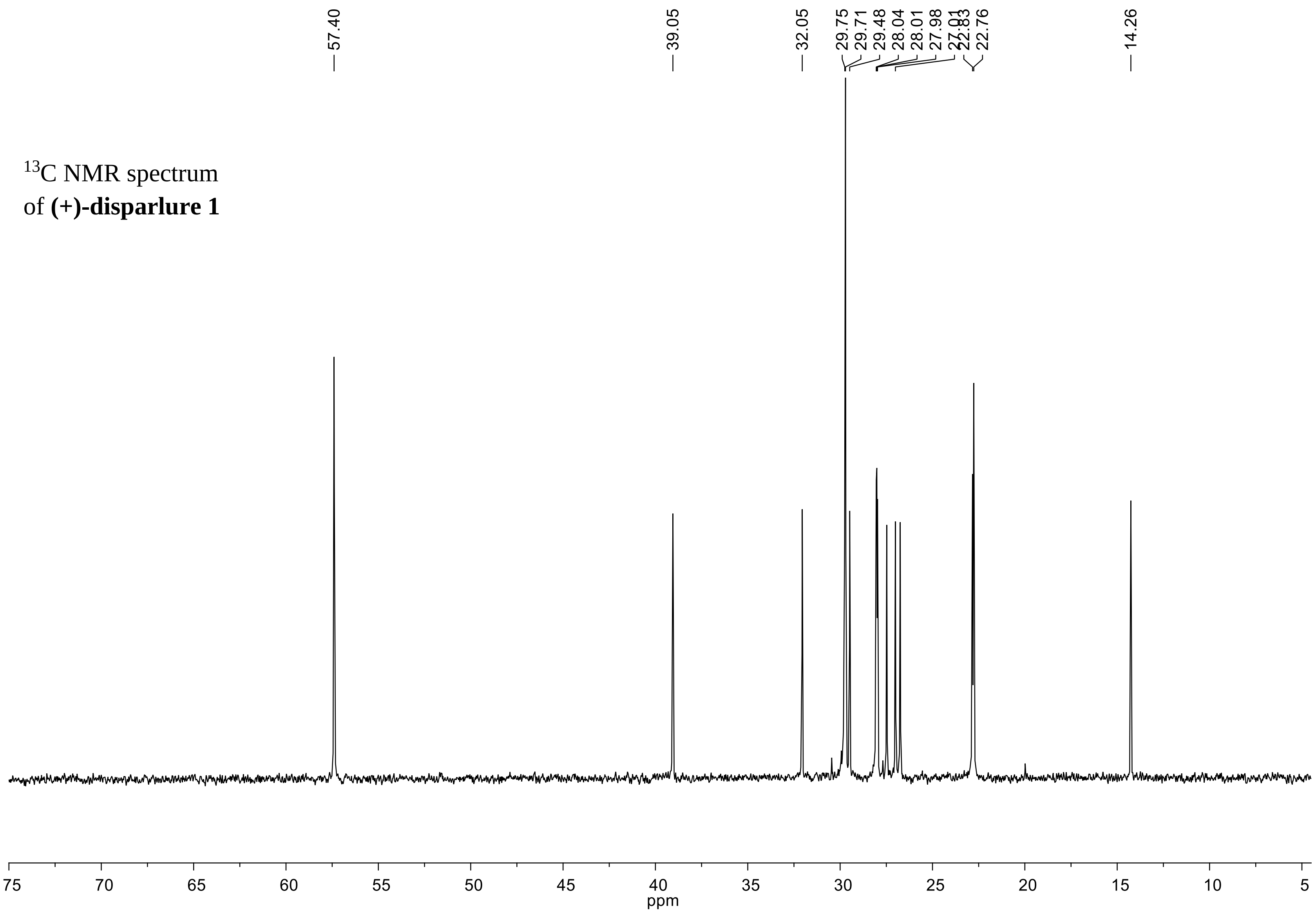

^{13}C NMR spectrum
of **(+)-disparlure 1**
57.40
39.05
32.05
29.75
29.71
29.48
28.04
28.01
27.98
27.01
22.83
22.76
14.26
75
70
65
60
55
50
45
40
35
30
25
20
15
10
5
ppm

^{1}H NMR spectrum
of **(+)-monachalure 2**

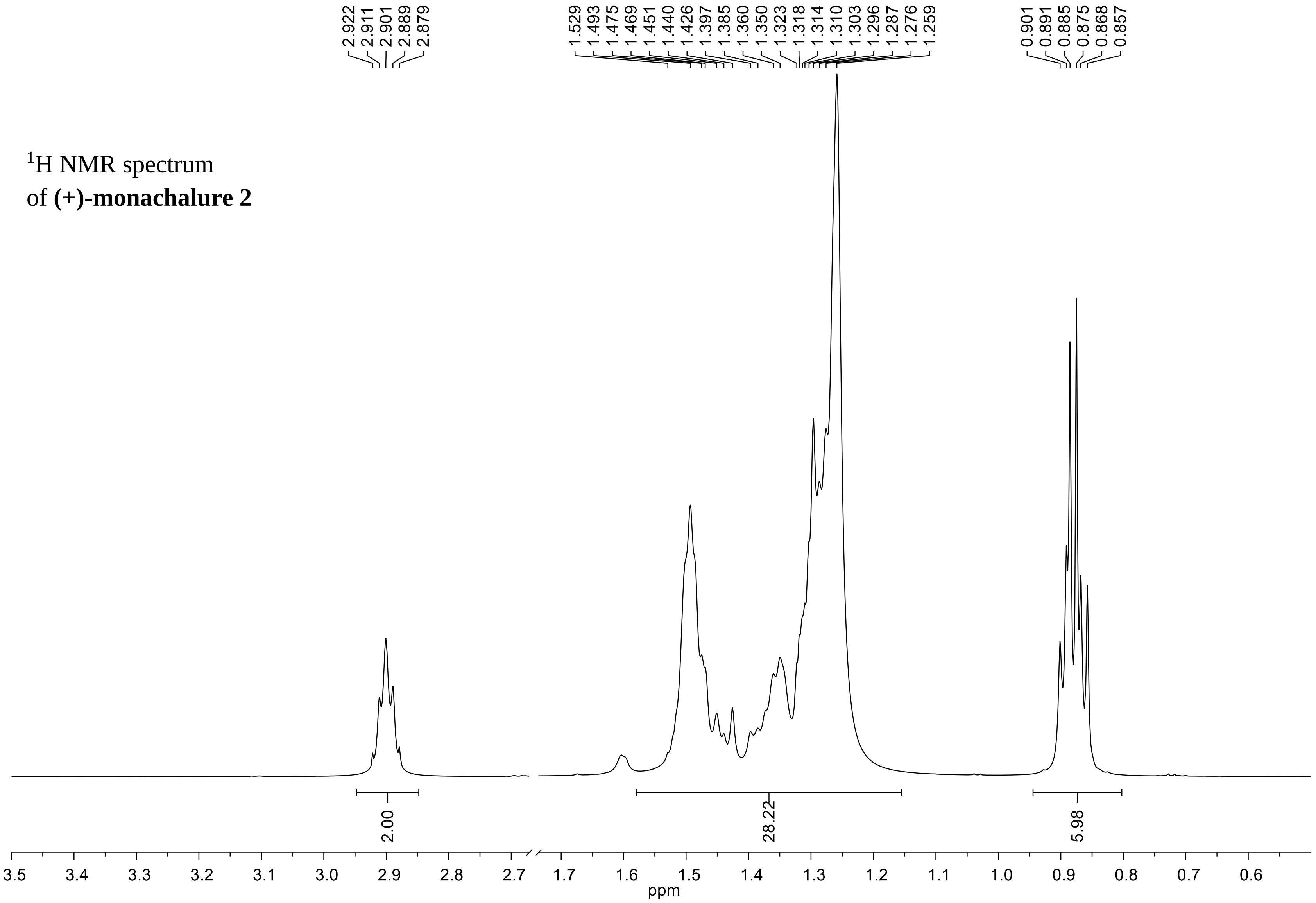

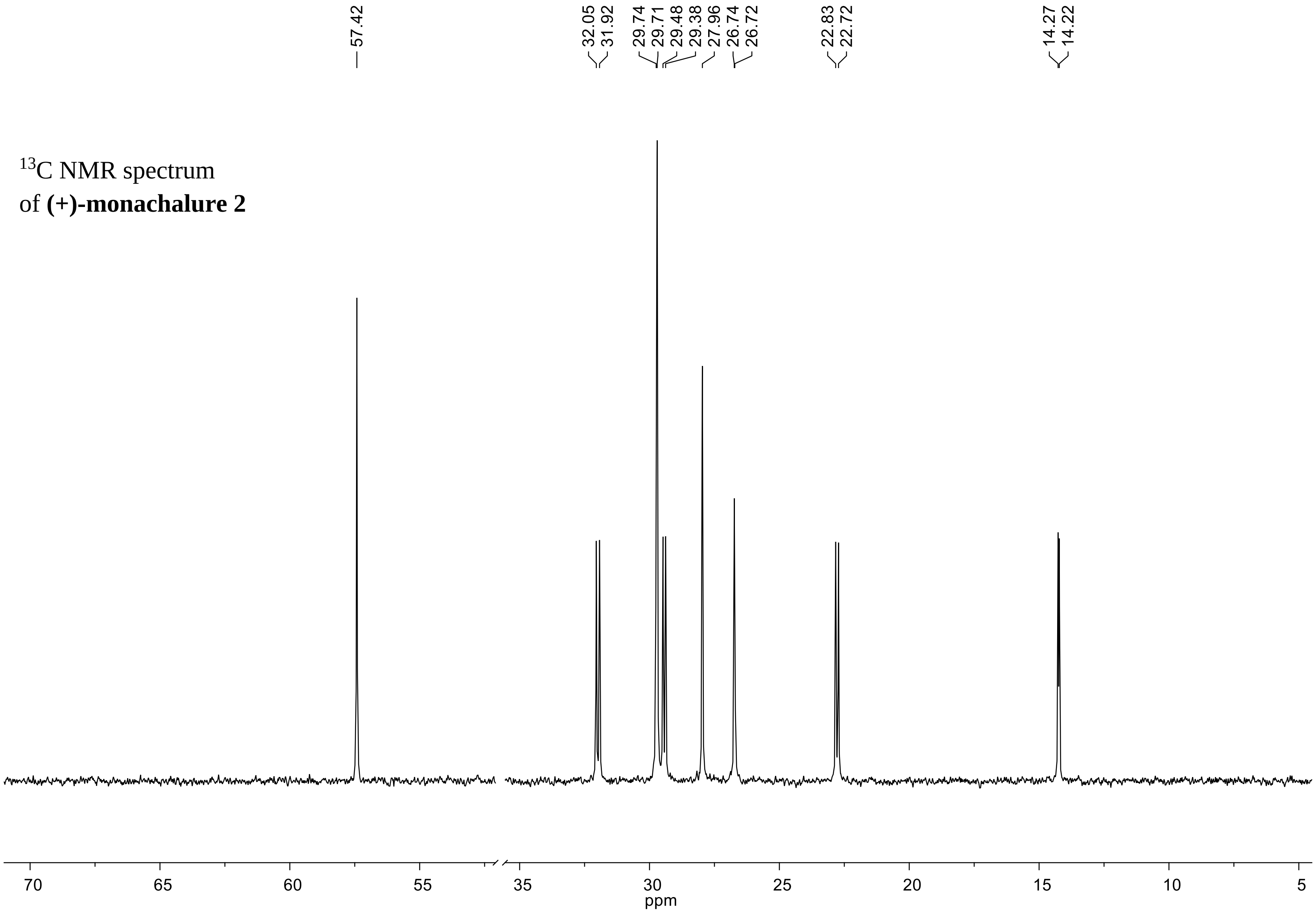
57.42
32.05
31.92
29.74
29.71
29.48
29.38
27.96
26.74
26.72
22.83
22.72
14.27
14.22
^{13}C NMR spectrum
of **(+)-monachalure 2**
70
65
60
55
35
30
25
20
15
10
5
ppm

^{1}H NMR spectrum of **24**

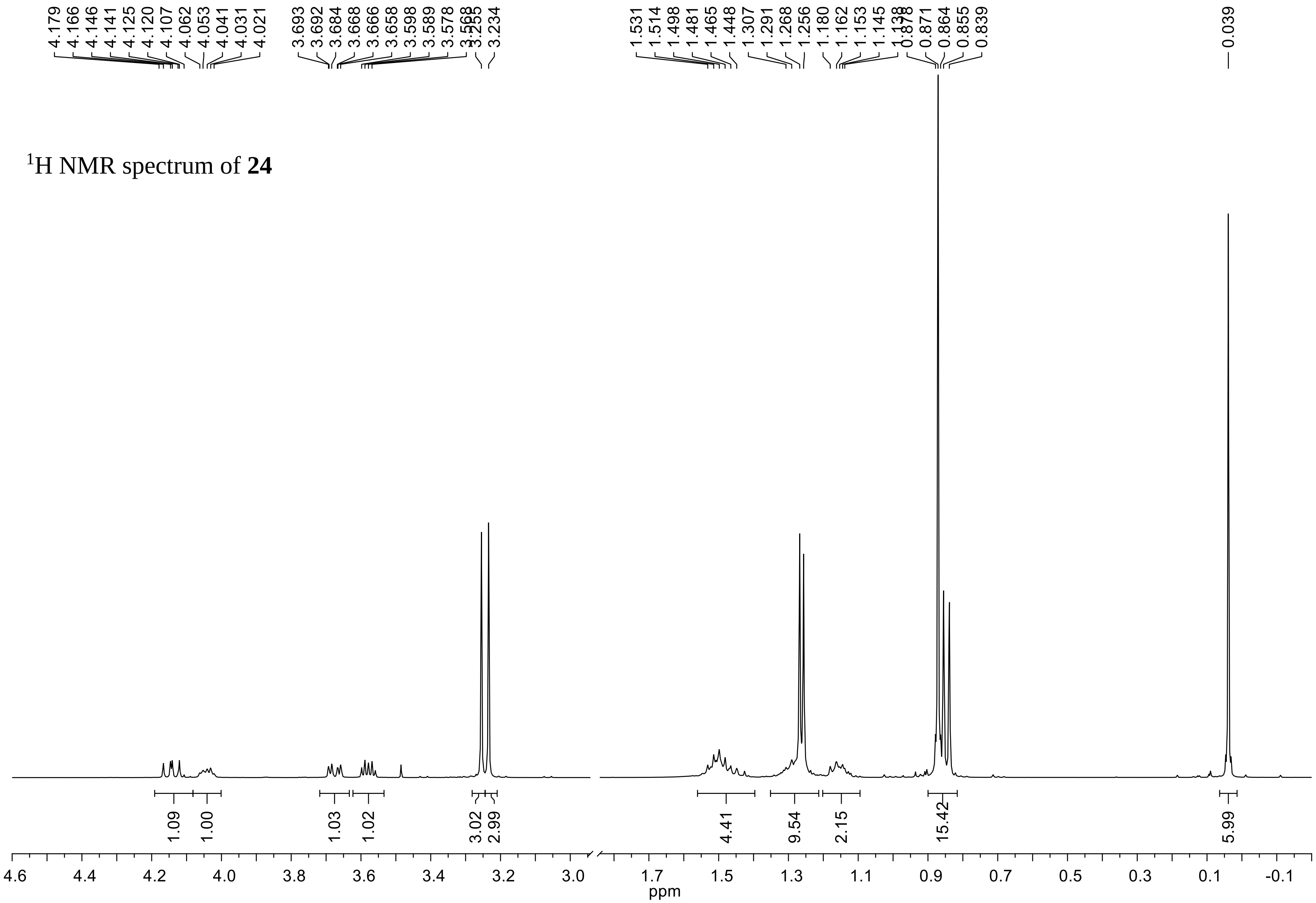

^{13}C NMR spectrum of **24**

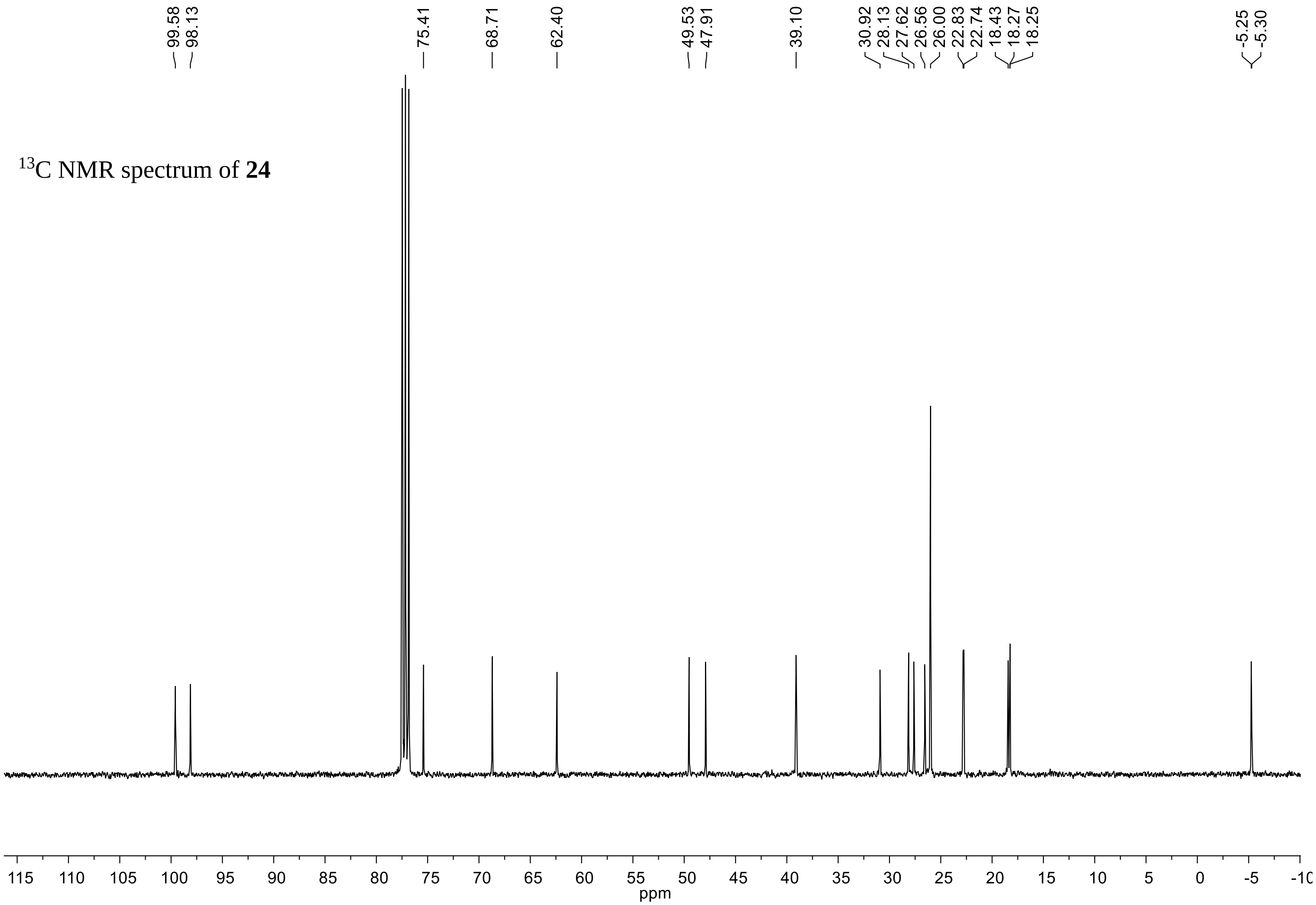

^{1}H NMR spectrum of **25**

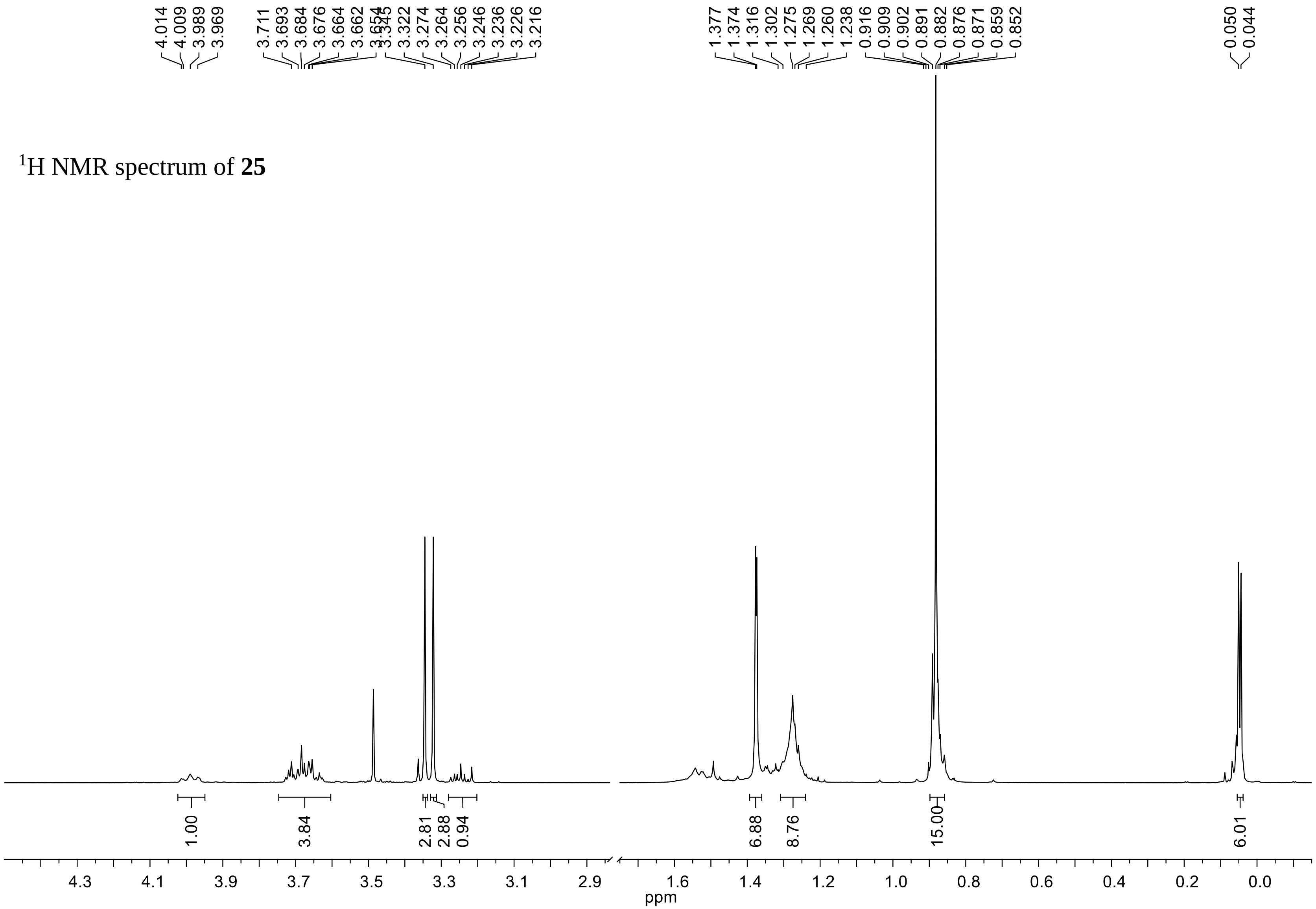

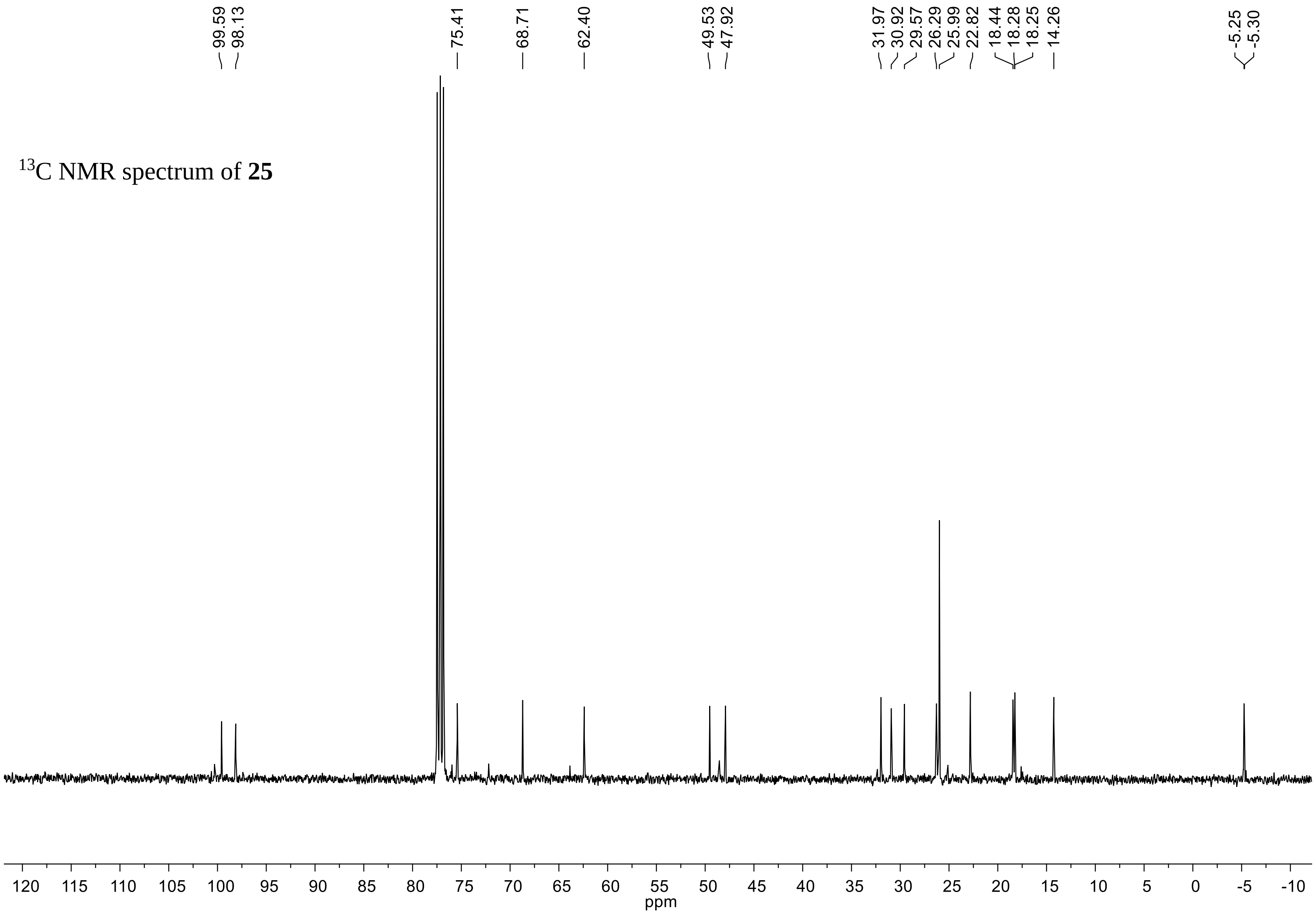

^{13}C NMR spectrum of **25**

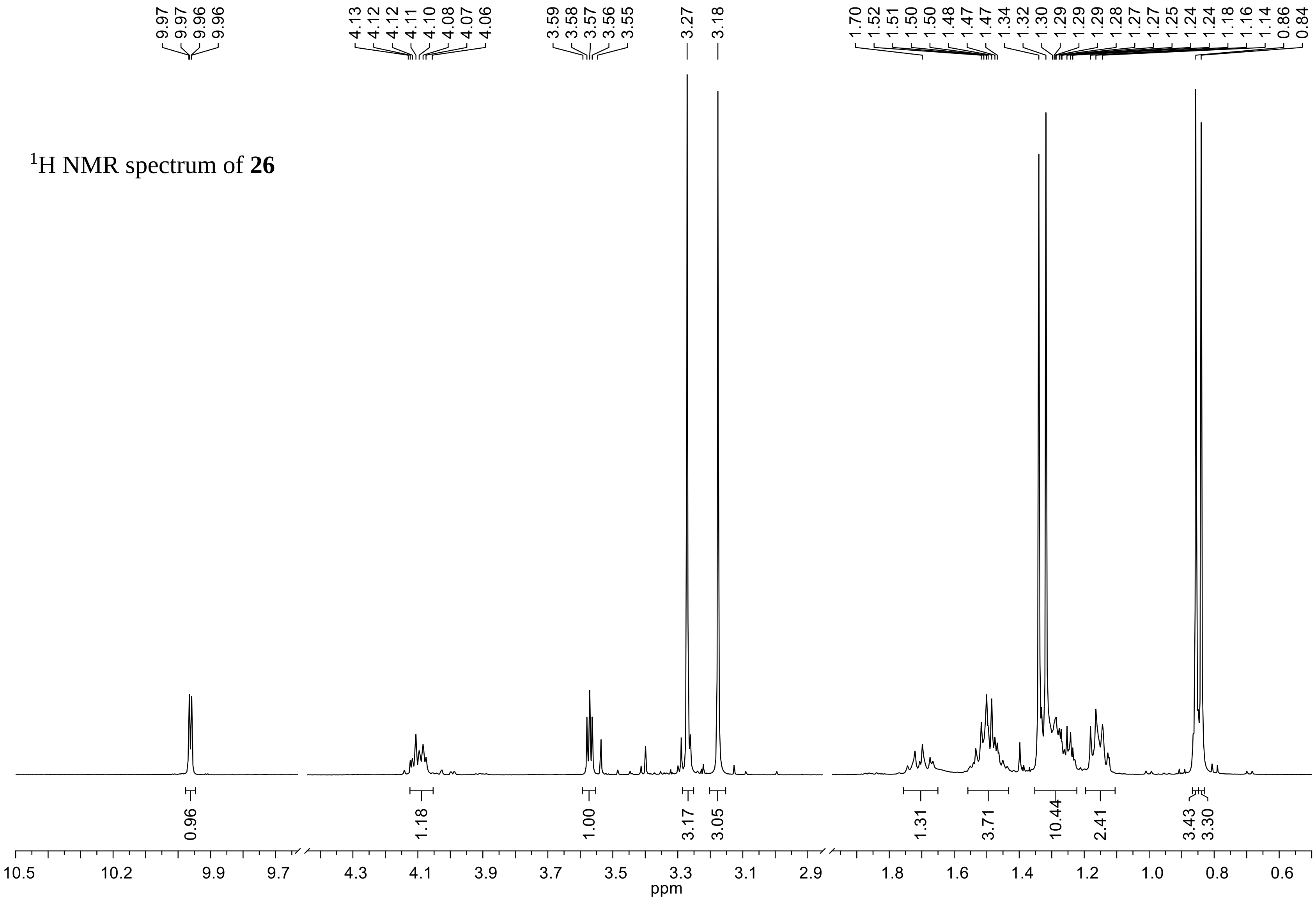

^{1}H NMR spectrum of **26**

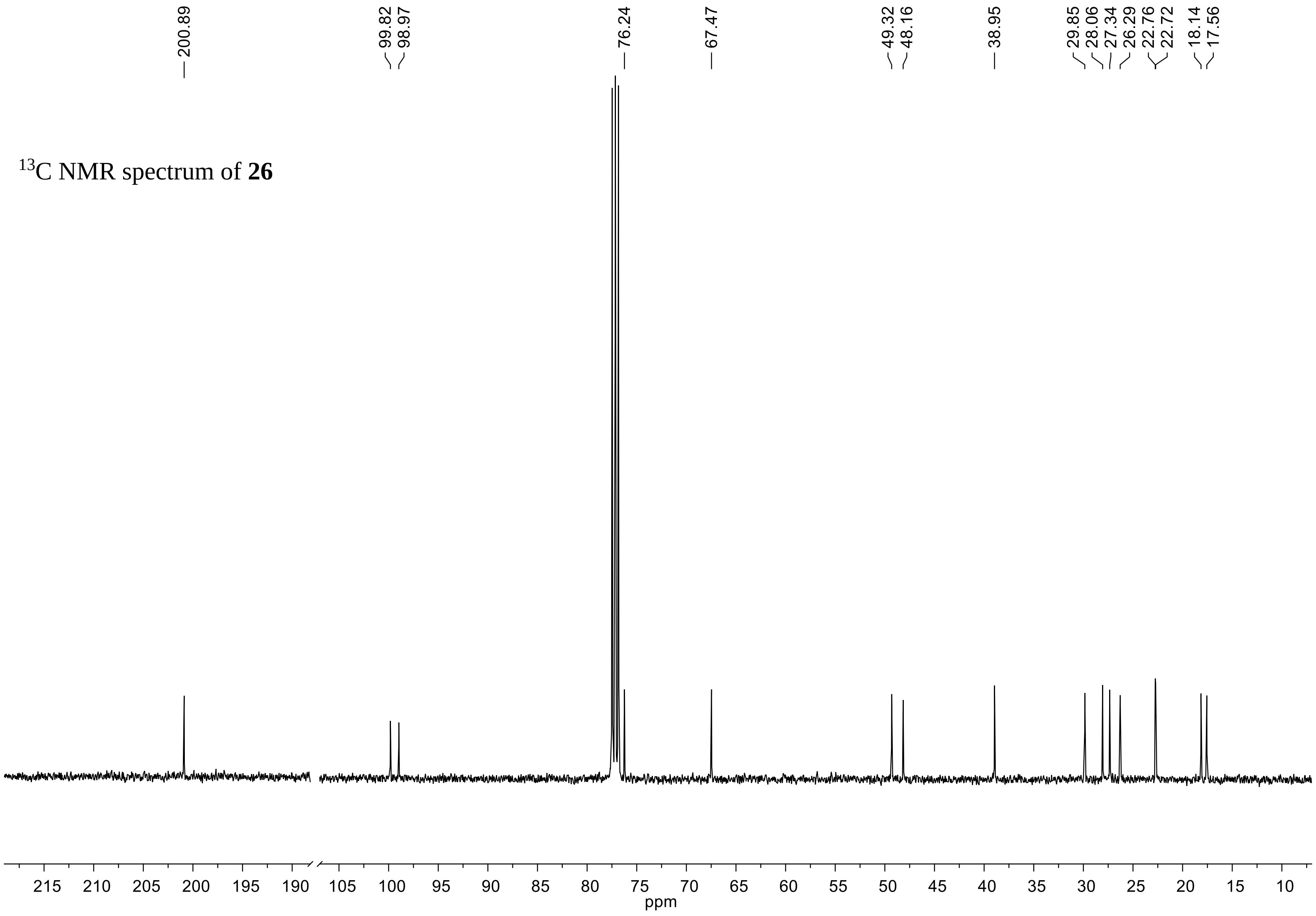

^{13}C NMR spectrum of **26**

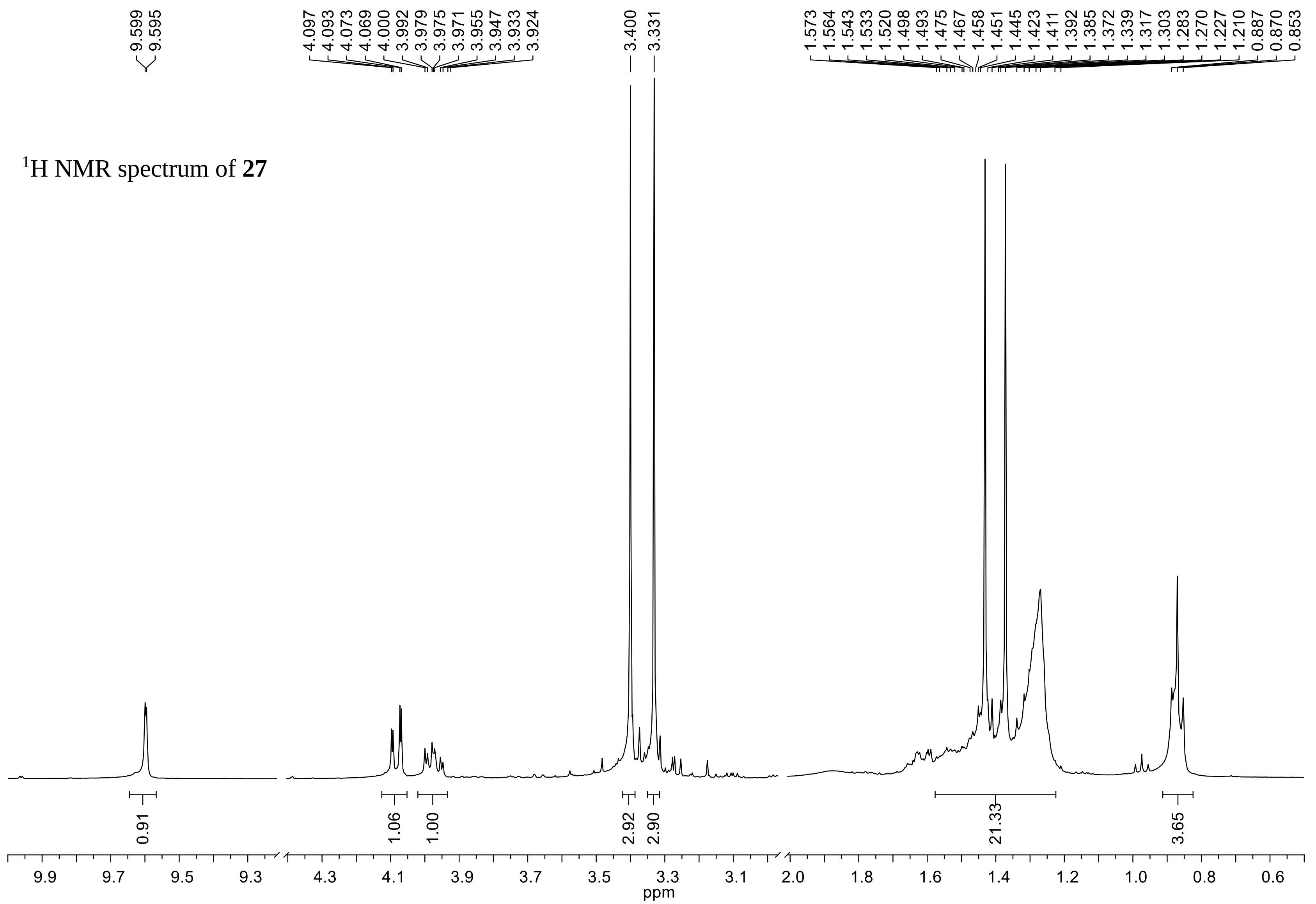

^{1}H NMR spectrum of **27**
9.599
9.595
4.097
4.093
4.073
4.069
4.000
3.992
3.979
3.975
3.971
3.955
3.947
3.933
3.924
3.400
3.331
1.573
1.564
1.543
1.533
1.520
1.498
1.493
1.475
1.467
1.458
1.451
1.445
1.423
1.411
1.392
1.385
1.372
1.339
1.317
1.303
1.283
1.270
1.227
1.210
0.887
0.870
0.853
0.91
1.06
1.00
2.92
2.90
21.33
3.65
9.9
9.7
9.5
9.3
4.3
4.1
3.9
3.7
3.5
3.3
3.1
2.0
1.8
1.6
1.4
1.2
1.0
0.8
0.6
ppm

^{1}H NMR spectrum of **28**

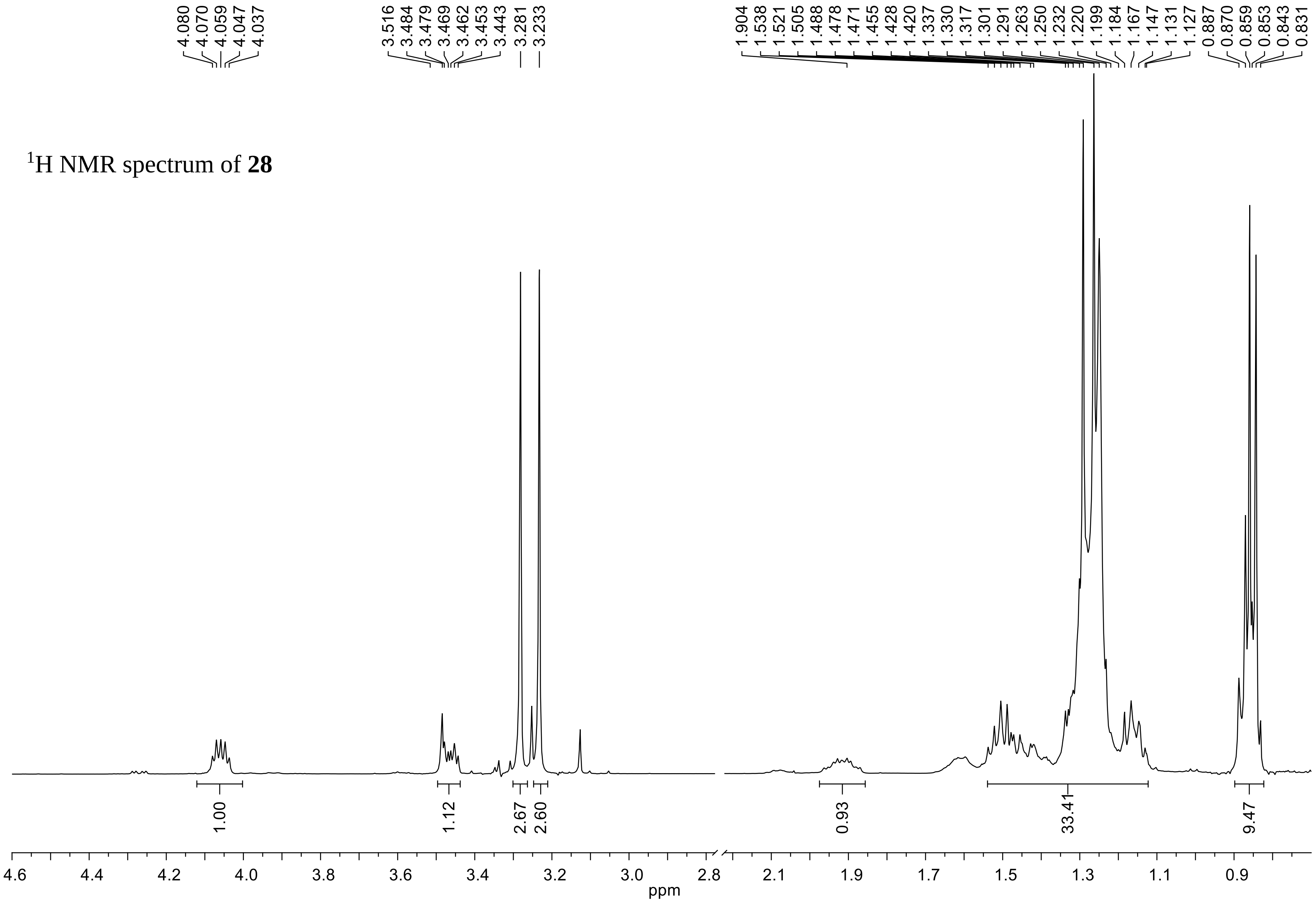

¹³C NMR spectrum of **28**

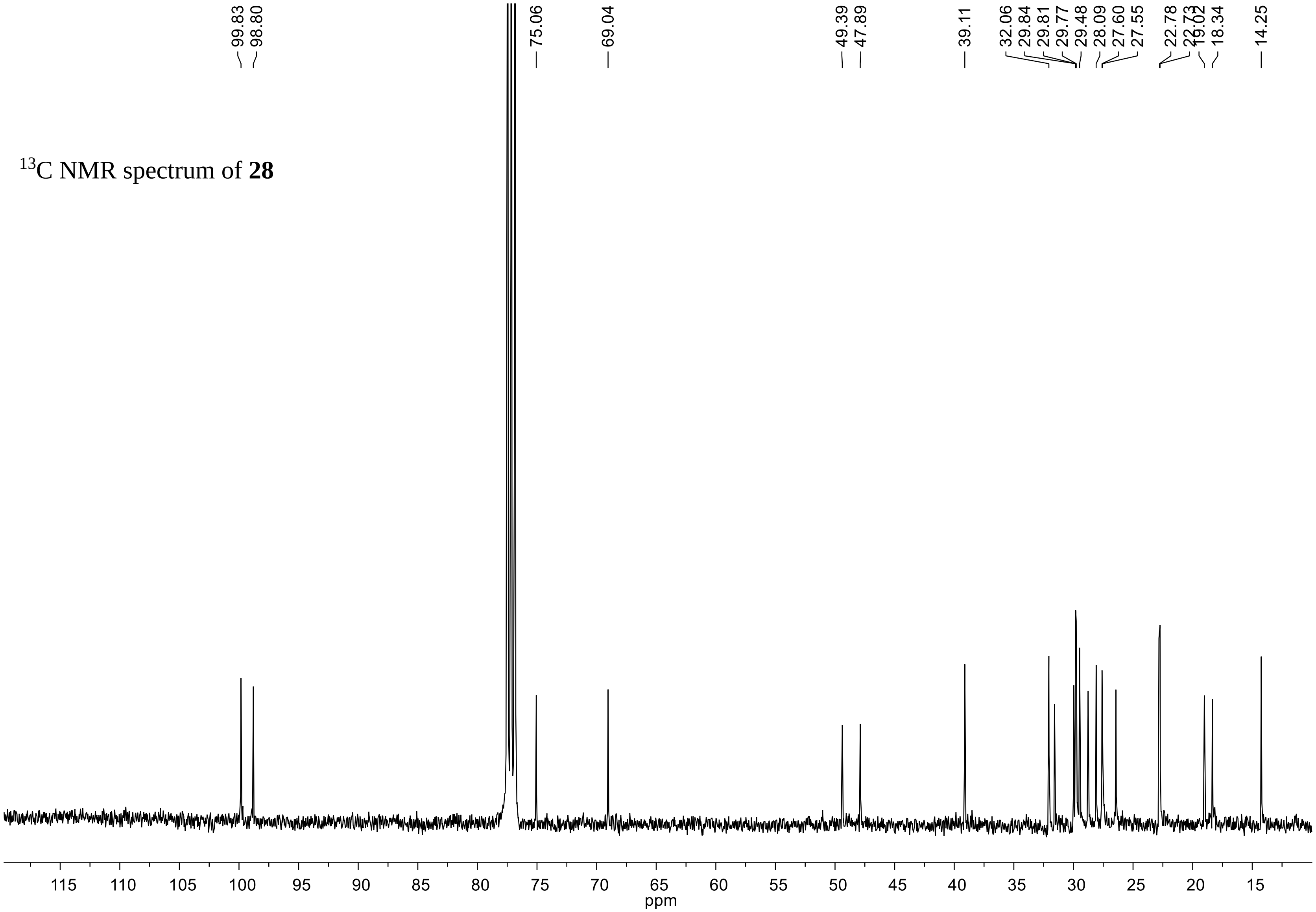

^{1}H NMR spectrum of **29**

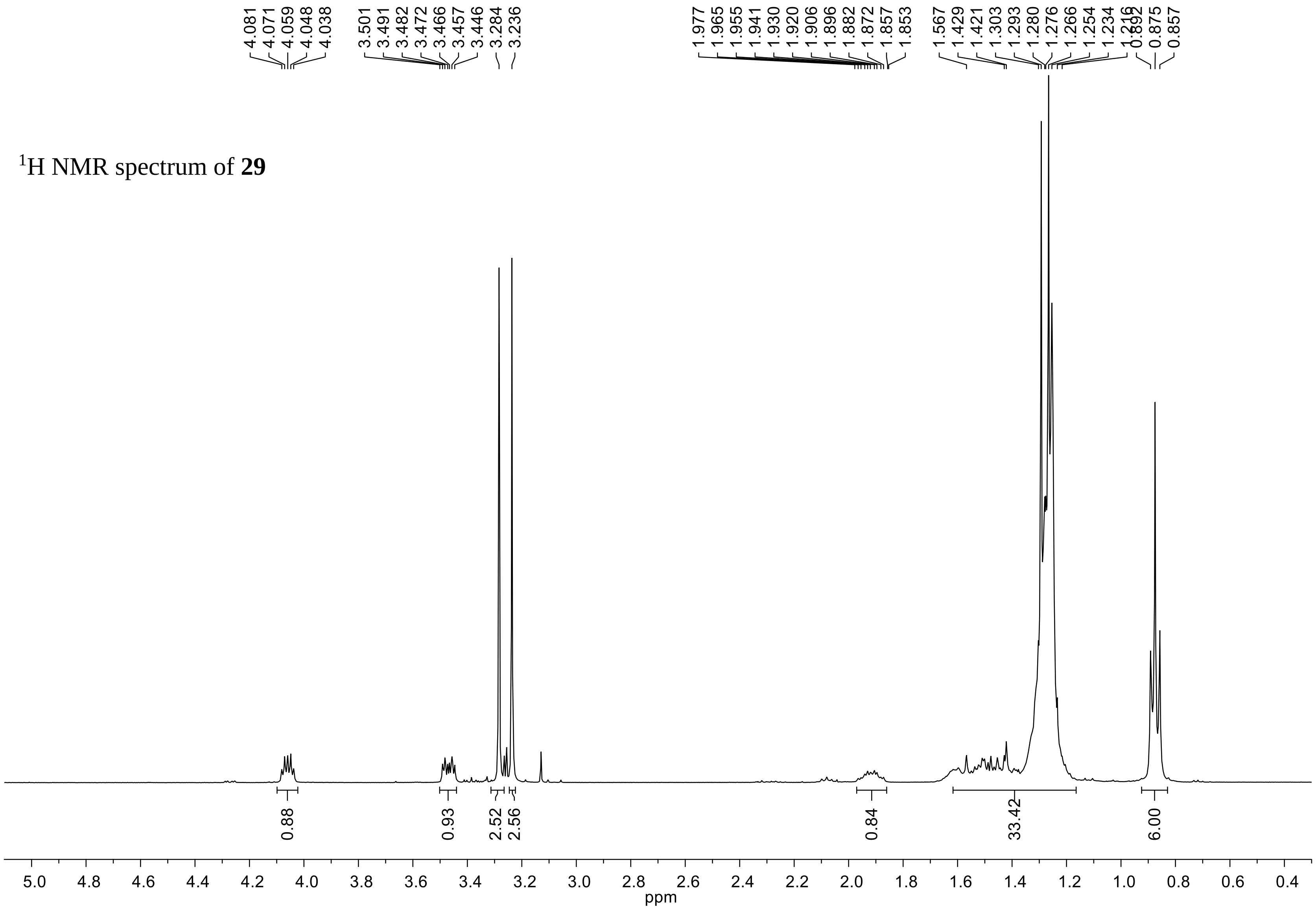

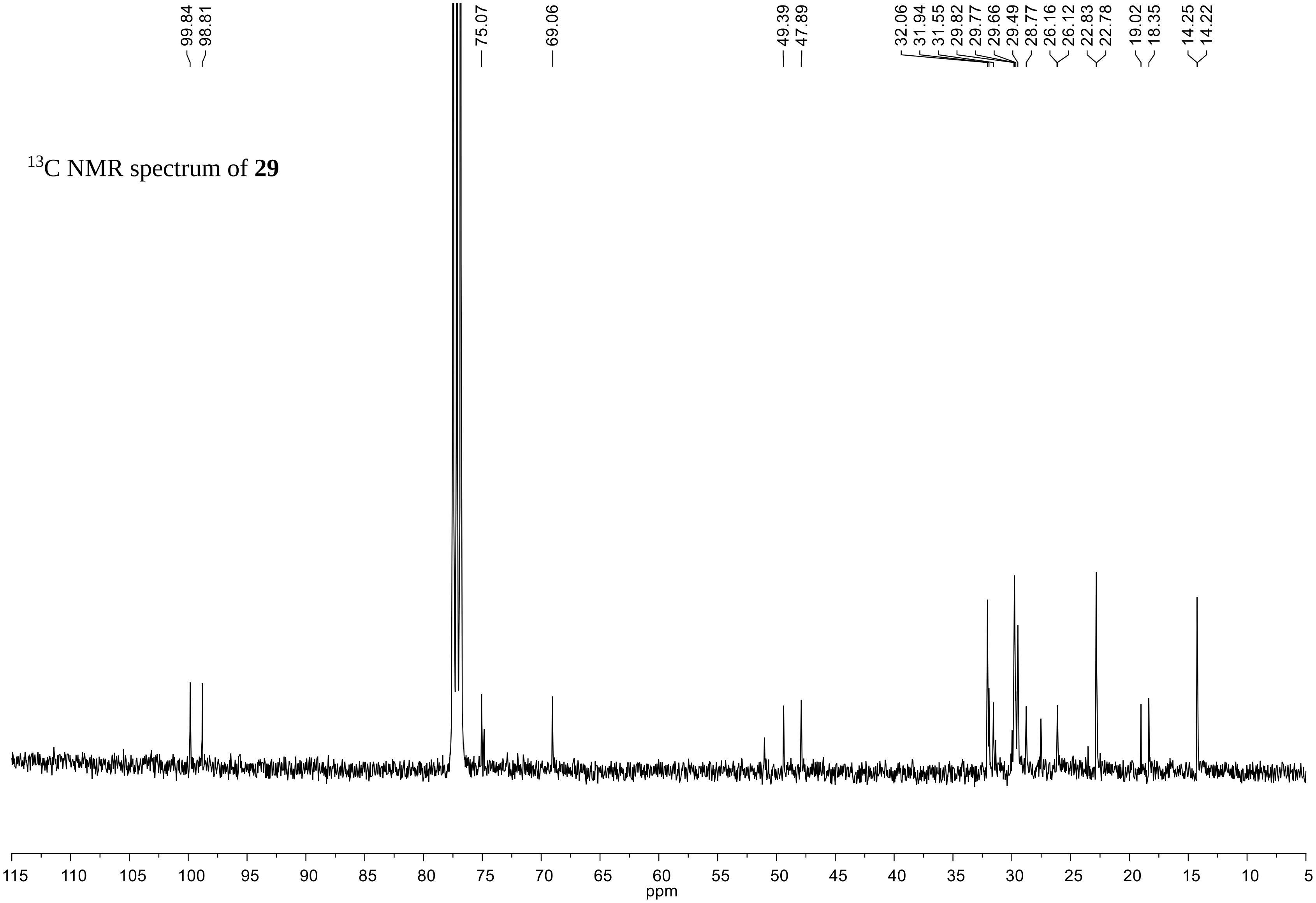

^{13}C NMR spectrum of **29**

^{1}H NMR spectrum of **7**

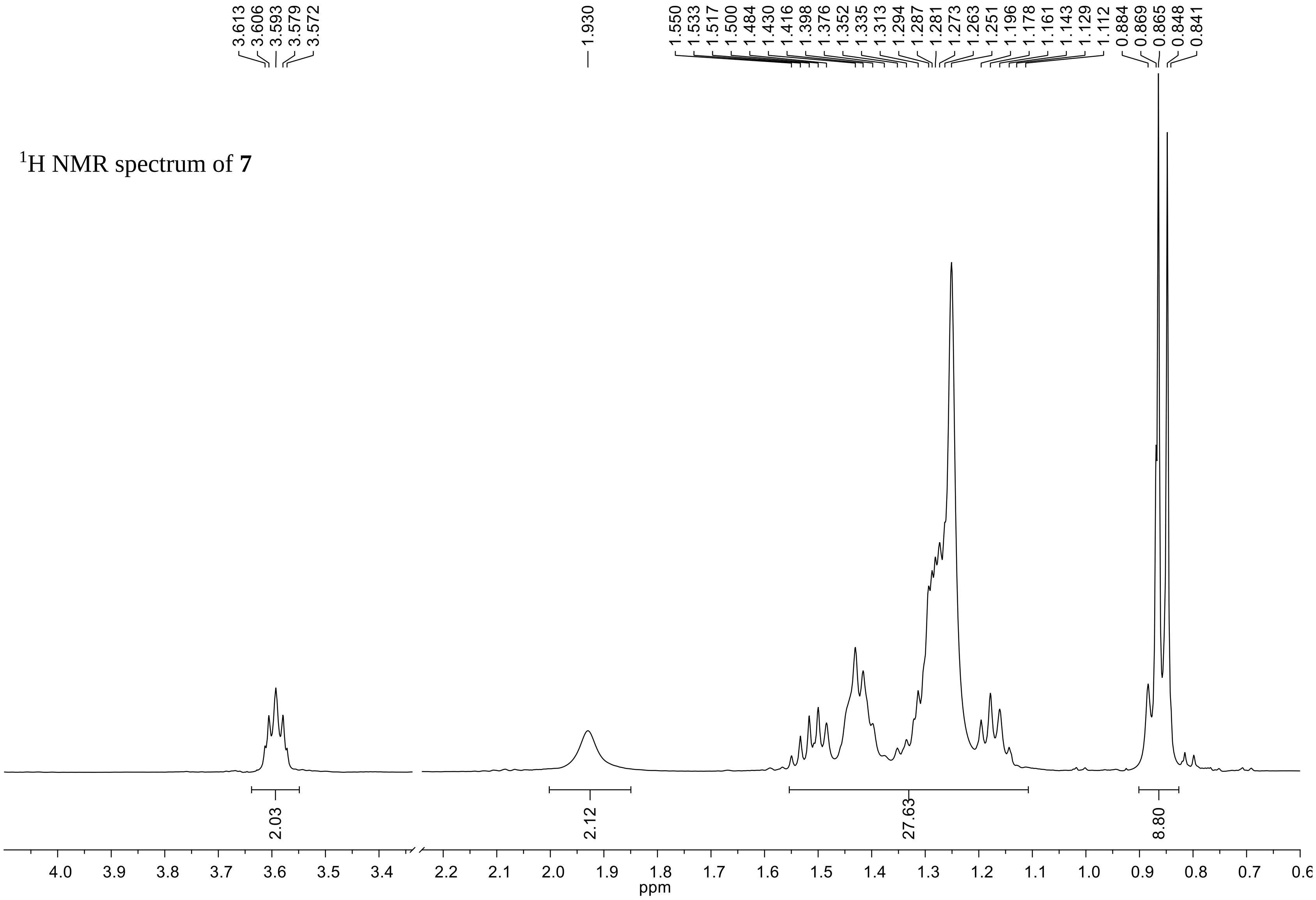

^{13}C NMR spectrum of **7**

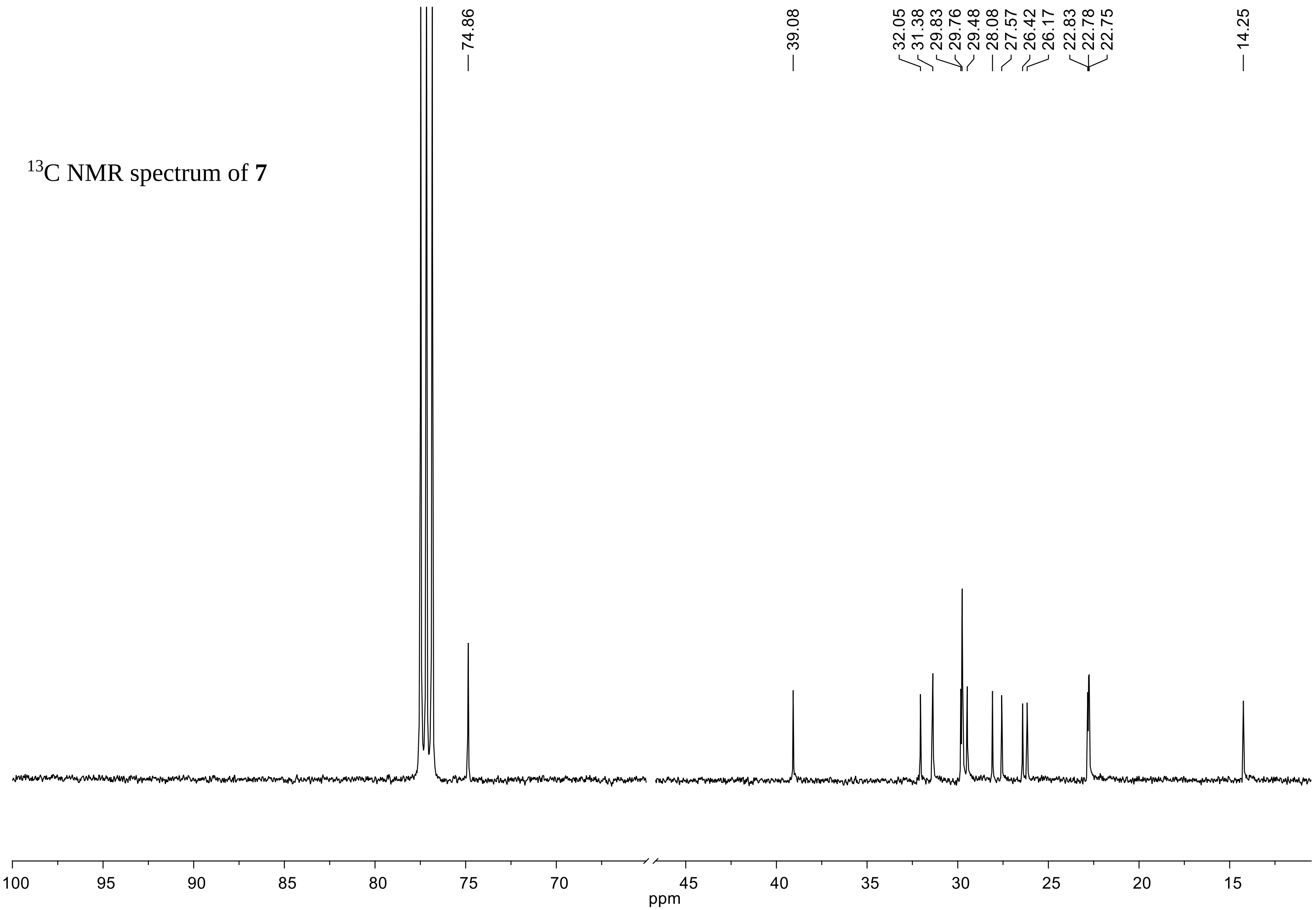

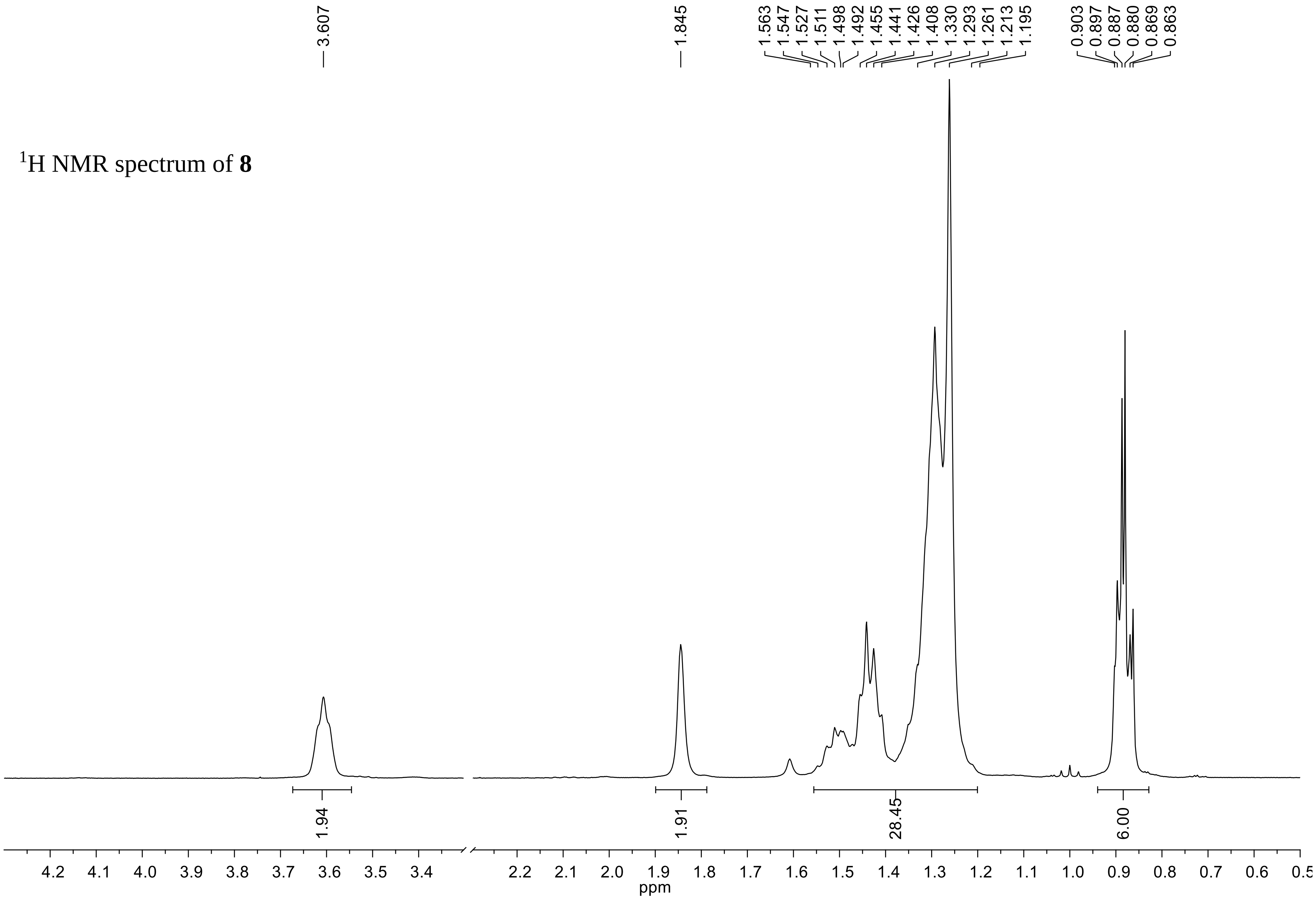

^{1}H NMR spectrum of **8**
3.607
1.845
1.563
1.547
1.527
1.511
1.498
1.492
1.455
1.441
1.426
1.408
1.330
1.293
1.261
1.213
1.195
0.903
0.897
0.887
0.880
0.869
0.863
1.94
1.91
28.45
6.00
4.2
4.1
4.0
3.9
3.8
3.7
3.6
3.5
3.4
2.2
2.1
2.0
1.9
1.8
1.7
1.6
1.5
1.4
1.3
1.2
1.1
1.0
0.9
0.8
0.7
0.6
ppm

13C NMR spectrum of **8**

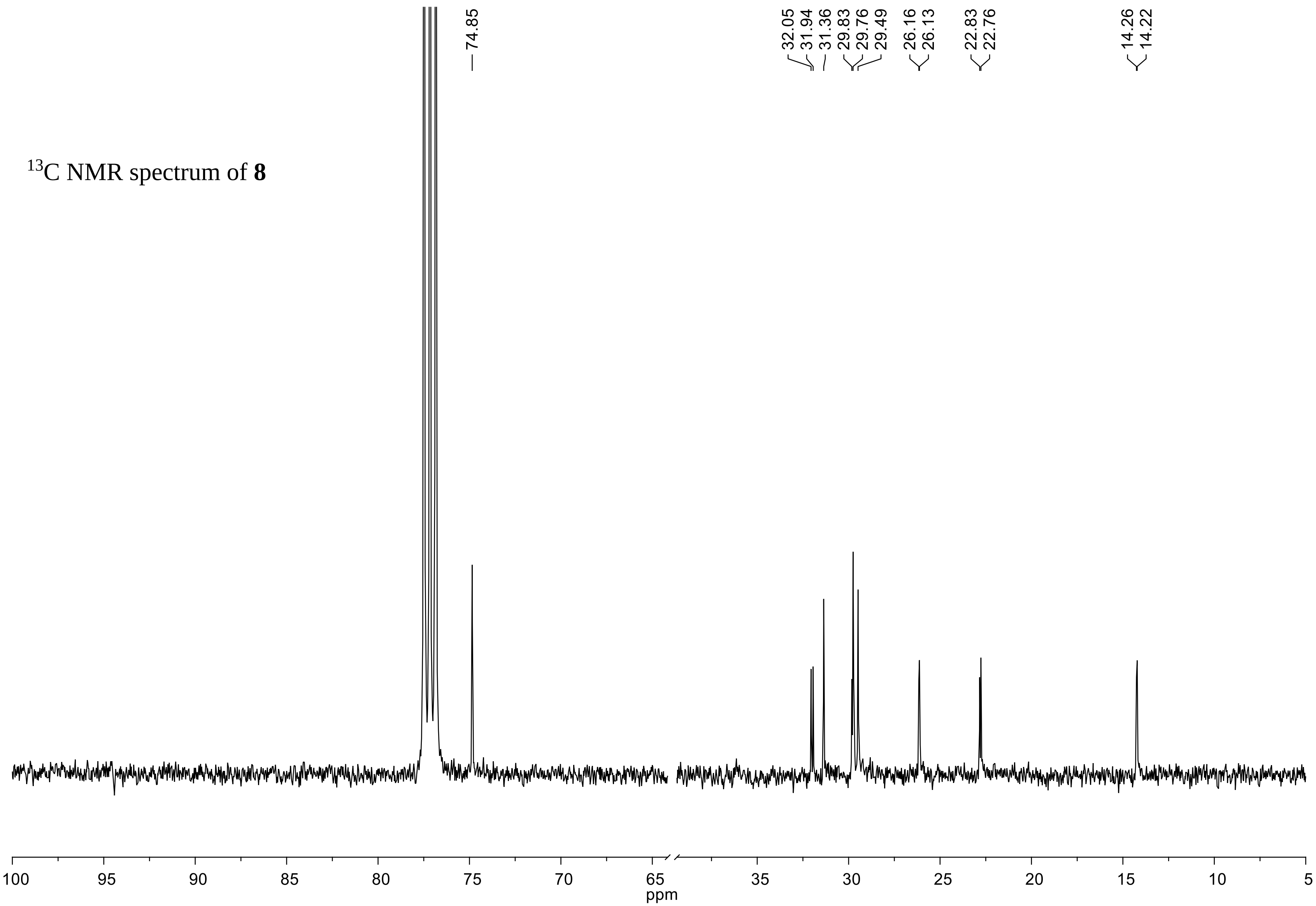

^{1}H NMR spectrum
of **(-)-disparlure 3**

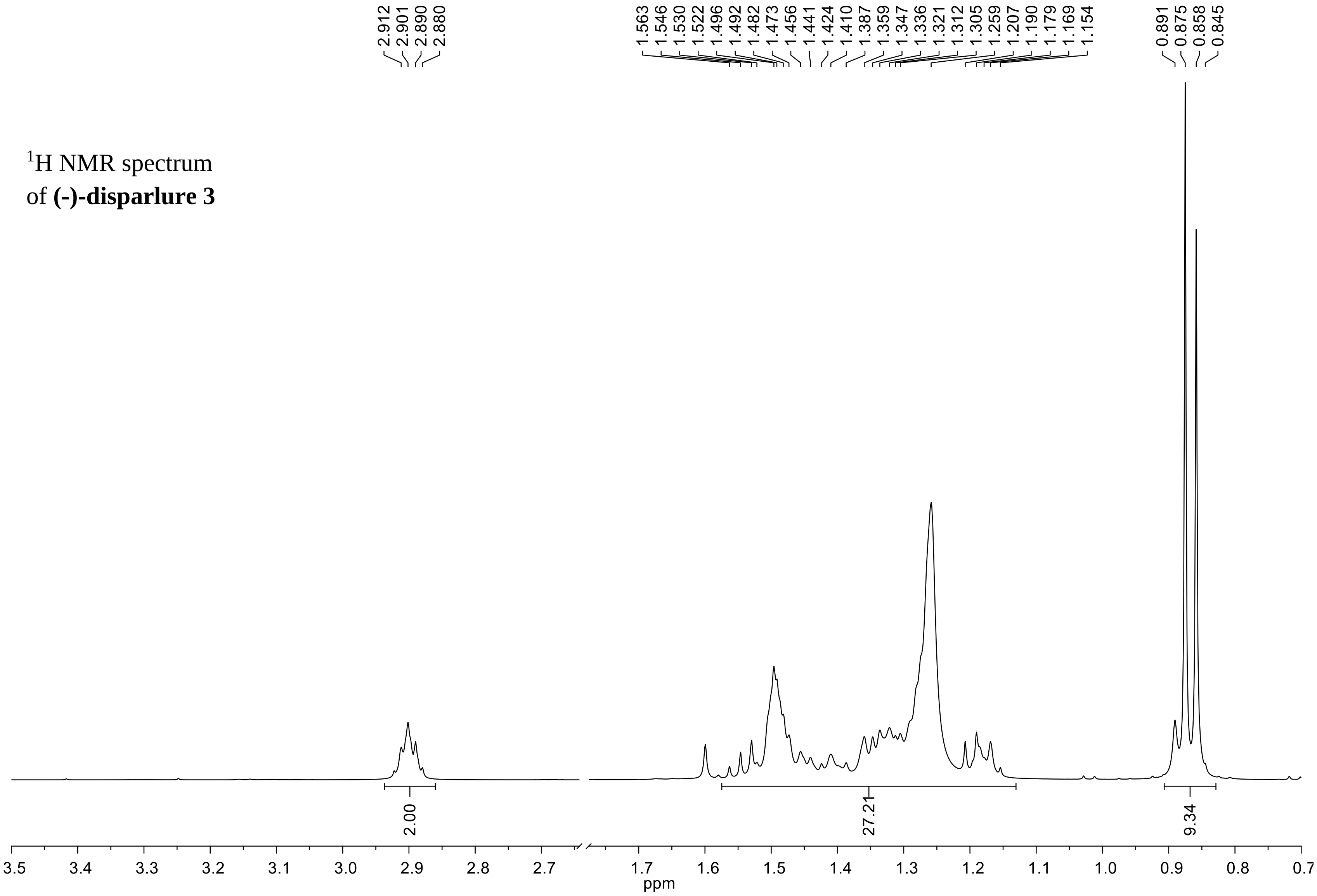

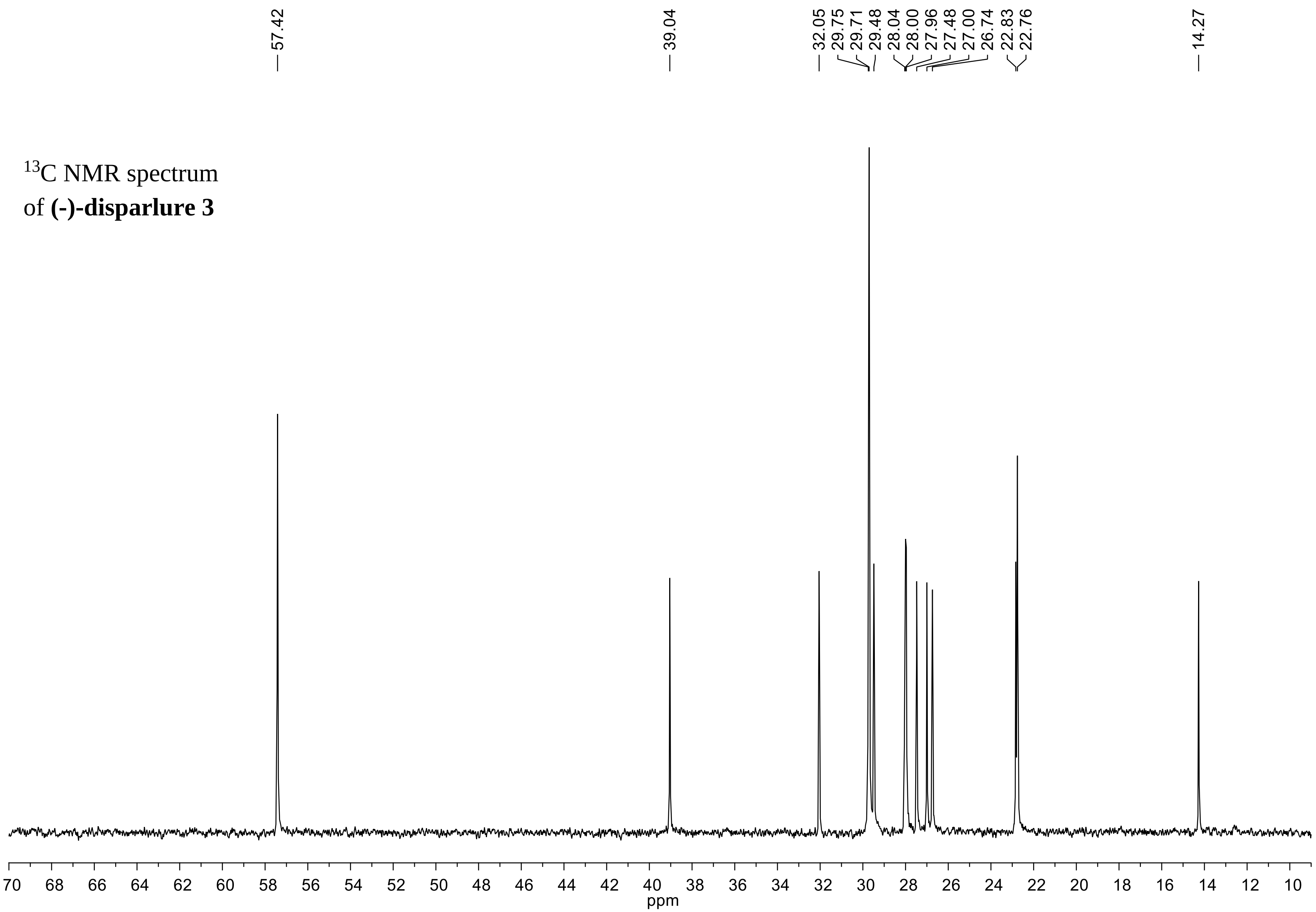

^{13}C NMR spectrum
of **(-)-disparlure 3**

^{1}H NMR spectrum
of **(-)-monachalure 4**

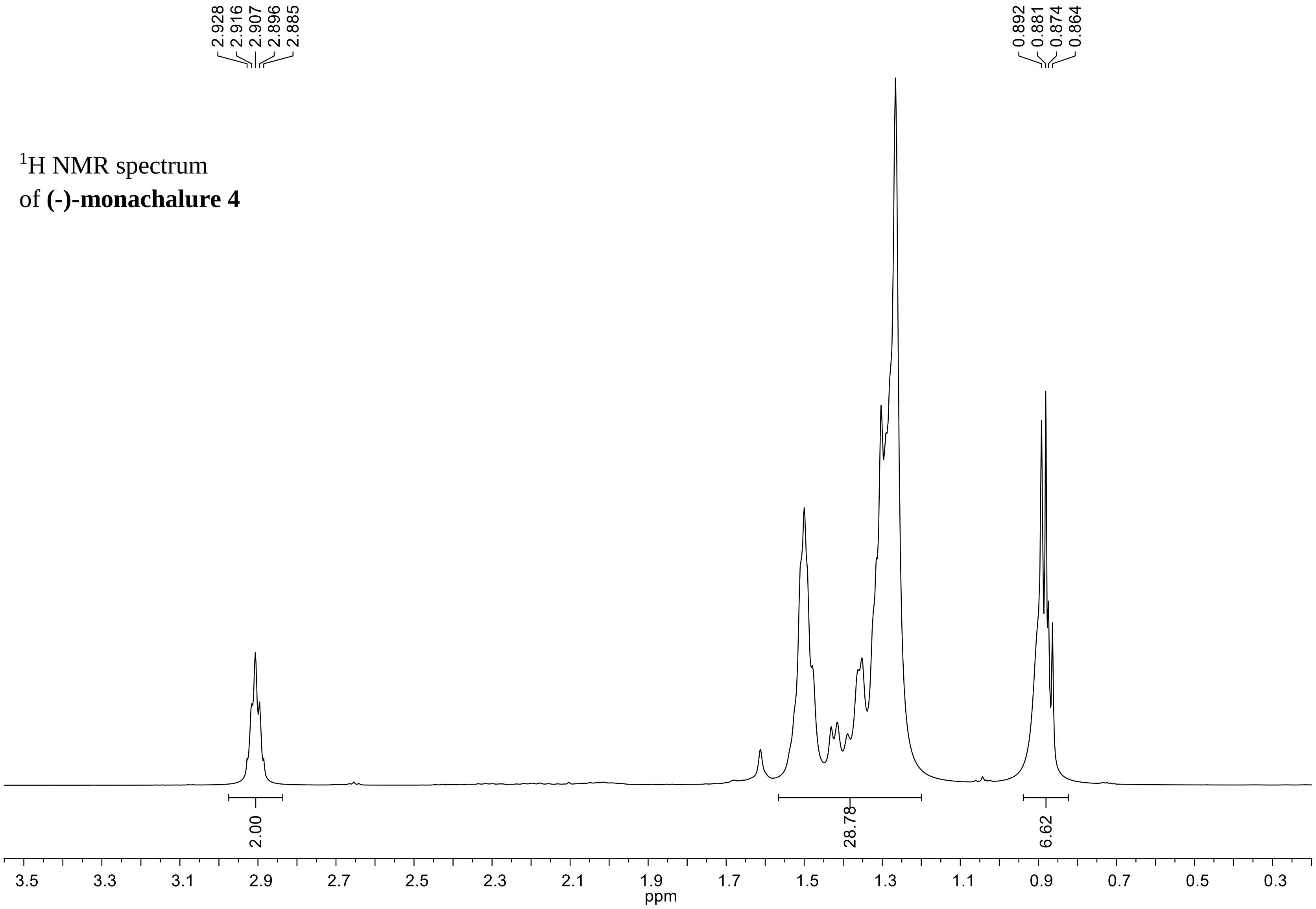

^{13}C NMR spectrum
of **(-)-monachalure 4**

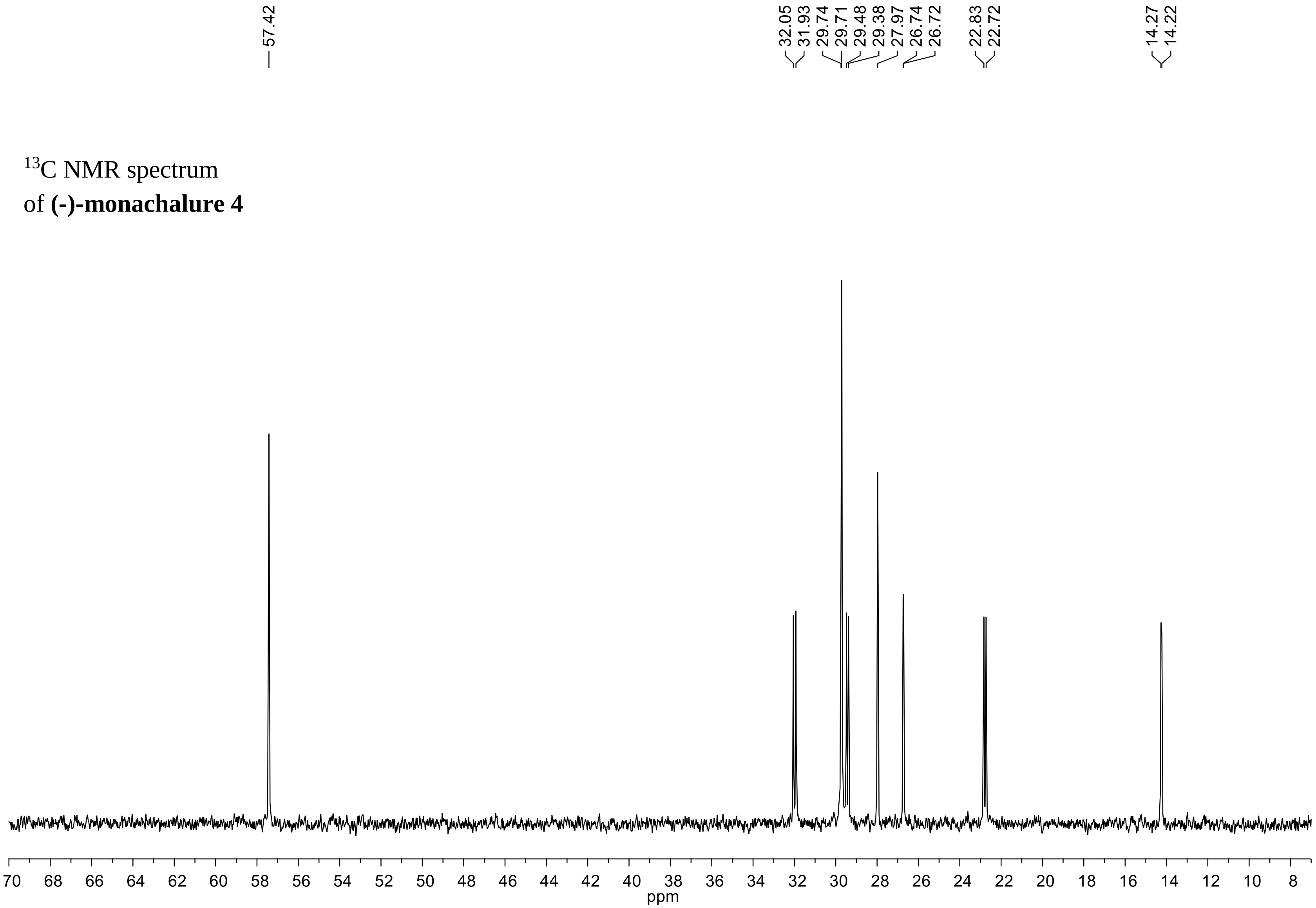

Supplement: File 2 — Copies of 1H NMR and 13C NMR spectra. [file Beilstein_J_Org_Chem-16-616-s002.pdf]
